# Effects of Nickel, Chlorpyrifos and Their Mixture on the *Dictyostelium discoideum* Proteome

**DOI:** 10.3390/ijms131215679

**Published:** 2012-11-23

**Authors:** Lara Boatti, Elisa Robotti, Emilio Marengo, Aldo Viarengo, Francesco Marsano

**Affiliations:** Department of Science & Technological Innovation (DiSIT), The University of Eastern Piedmont Amedeo Avogadro, Alessandria, Novara, Vercelli, Viale Teresa Michel, 11-15121 Alessandria, Italy; E-Mails: lara.boatti@mfn.unipmn.it (L.B.); elisa.robotti@mfn.unipmn.it (E.R.); emilio.marengo@mfn.unipmn.it (E.M.); aldo.viarengo@mfn.unipmn.it (A.V.)

**Keywords:** *Dictyostelium discoideum*, toxicity, nickel, chlorpyrifos, proteomics, mass spectrometry

## Abstract

Mixtures of chemicals can have additive, synergistic or antagonistic interactions. We investigated the effects of the exposure to nickel, the organophosphate insecticide chlorpyrifos at effect concentrations (EC) of 25% and 50% and their binary mixture (Ec25 + EC25) on *Dictyostelium discoideum* amoebae based on lysosomal membrane stability (LMS). We treated *D. discoideum* with these compounds under controlled laboratory conditions and evaluated the changes in protein levels using a two-dimensional gel electrophoresis (2DE) proteomic approach. Nickel treatment at EC25 induced changes in 14 protein spots, 12 of which were down-regulated. Treatment with nickel at EC50 resulted in changes in 15 spots, 10 of which were down-regulated. Treatment with chlorpyrifos at EC25 induced changes in six spots, all of which were down-regulated; treatment with chlorpyrifos at EC50 induced changes in 13 spots, five of which were down-regulated. The mixture corresponding to EC25 of each compound induced changes in 19 spots, 13 of which were down-regulated. The data together reveal that a different protein expression signature exists for each treatment, and that only a few proteins are modulated in multiple different treatments. For a simple binary mixture, the proteomic response does not allow for the identification of each toxicant. The protein spots that showed significant differences were identified by mass spectrometry, which revealed modulations of proteins involved in metal detoxification, stress adaptation, the oxidative stress response and other cellular processes.

## 1. Introduction

Understanding the modes of action of single compounds has been a challenging task. A single compound can elicit agonistic actions on one biological pathway but antagonistic responses on another pathway. Therefore, contradictory results can be obtained depending on the endpoint studied [1,2]. Moreover, environmental pollution rarely exists as a single contaminant, resulting in many chances for multi-compound interactions. These interactions can strongly influence the effects of chemical stressors on organisms. There are considerable gaps in knowledge about the *in vivo* effects of toxic mixtures. Although a number of studies have investigated individual compounds of concern, exposure studies that focus on chemical mixtures are largely missing from the literature.

A number of toxico-genomic studies have invested large efforts towards a better understanding of mixture toxicity, but the complex interplay of toxicants among several biological pathways remains difficult to resolve [3–5]. Nevertheless, single-endpoint mixture studies generate valuable information, specifically when compound-related biomarkers are used (e.g., the loss of acetylcholinesterase activity in relation to organophosphate and carbamate pesticide exposure) [6]. Acquiring in-depth information about the interactions of compounds requires techniques that can provide an overview of several endpoints and pathways simultaneously, such as proteomic approaches. Currently, only a limited number of studies have applied proteomics techniques to the evaluation of mixture toxicity responses.

The application of recent “omics”-technologies in (eco)toxicology depends on the assumption that all toxicologically relevant effects are accompanied by alterations in multiple genes or proteins. This application is particularly promising because classical chemical analyses can only quantify substances that are known in advance and are present in relatively high concentrations in the environment, but they cannot be used to assess environmental and biological conditions with respect to chemical bioavailability and the effects of toxicants *in vivo*. Therefore, the best “analyzer” of these complex effects on organisms is the exposed organism itself [7]. *Dictyostelium discoideum* has been recognized as a model organism that can live on humus and decaying leaves, so it is widely exposed to toxicants in soils and could be a suitable biomonitoring organism for soil pollutants.

Nickel (Ni) is of great environmental concern; moreover, within a cell, its chemical form may be altered, and it exerts long-term harmful effects [8]. It can be found naturally in metalliferous soils, but it is often found in the environment as a result of industrial discharges from electroplating, smelting, mining and refining operations, and other industrial emissions [9]. Compared to other divalent metals, nickel toxicity has been studied mainly in water species [10–12]. A recent study monitored the expression of genes involved in the specific molecular response to nickel in a nickel-tolerant fungus [13]. Although several toxicological studies have investigated the effects of Ni in various organisms, the underlying molecular mechanisms by which Ni causes cellular damage are poorly understood [14]. Moreover, the cellular fate of nickel is interesting for phytoremediation applications, but the core mechanism of the molecules involved and the physiological conditions required, including soil absorption, neutralization and toxicity, remain elusive [15].

Chlorpyrifos (CHP) is a broad-spectrum organophosphate pesticide with many urban and agricultural pest control applications. CHP forms the active ingredient in several insecticides and is among the most widely used insect control products [16]. Poisoning from CHP could affect the central nervous system, cardiovascular system, and respiratory system; it also acts as a skin and eye irritant. The major and well-known toxic effects of CHP involve the nervous system, as CHP is an inhibitor of the enzyme acetylcholine esterase (AChE) [17]; other possible toxic effects on environmentally important organisms are largely unknown. *D. discoideum* do not have a nervous system, and given the evidence of the activity of CHP against AChE [18], this organism could be an appropriate model to study the underlying effects of CHP and other neurotoxic compounds on the proteome under conditions where other organisms would suffer from rapid mortality.

Numerous potential molecular targets for CHP (in addition to AChE) have been identified, including effects on macromolecule synthesis (DNA, RNA, and proteins) and oxidative stress and effects on microtubules [19–24].

Further information concerning the effects of nickel and CHP and their mixture on the cellular proteome is crucial for understanding the molecular mechanisms involved in their toxic effects and allows for further studies in biomonitoring, phytoremediation and toxicological sciences.

The aim of the present study is to characterize the toxic effects and the proteomic responses in the model organism *Dictyostelium discoideum* to CHP and nickel. Additionally, we compared the responses to the individual compounds with those of their binary equitoxic mixture using lysosomal membrane stability (LMS). The final objective was to evaluate the variation in the protein expression signature (PES) when cells are exposed to different concentrations of single compounds or a binary equitoxic mixture.

## 2. Results

### 2.1. Experimental Design and Physiological Responses in *D. discoideum* Cells Exposed to Nickel, CHP and Their Mixture

#### 2.1.1. Mortality Rate

To evaluate the effect of the toxicants on cells, *D. discoideum* cells were exposed to increasing concentrations of NiCl_2_ (0–10.0 mM) and the organophosphate pesticide CHP (0–50 μM) for 3 h under laboratory conditions.

**Figure 1:**
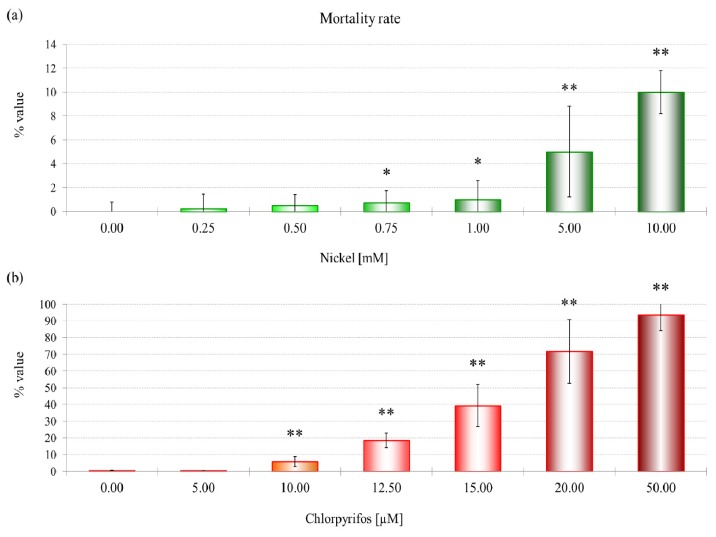
The effects of nickel (**a**) and chlorpyrifos (**b**) on the mortality rate of *Dictyostelium discoideum* amoebae. A statistical significance of *p* ≤ 0.05 is indicated as *, and a statistical significance of *p* ≤ 0.01 is indicated as ** (Mann-Whitney test).

The first significant effects on mortality in *D. discoideum* (LOEC, low effect concentration) were observed after treatment with 0.75 mM NiCl_2_ (Figure 1a), while a significant increase in this parameter was induced after treatment with 10 μM CHP (Figure 1b).

#### 2.1.2. Lysosomal Membrane Stability

**Figure 2:**
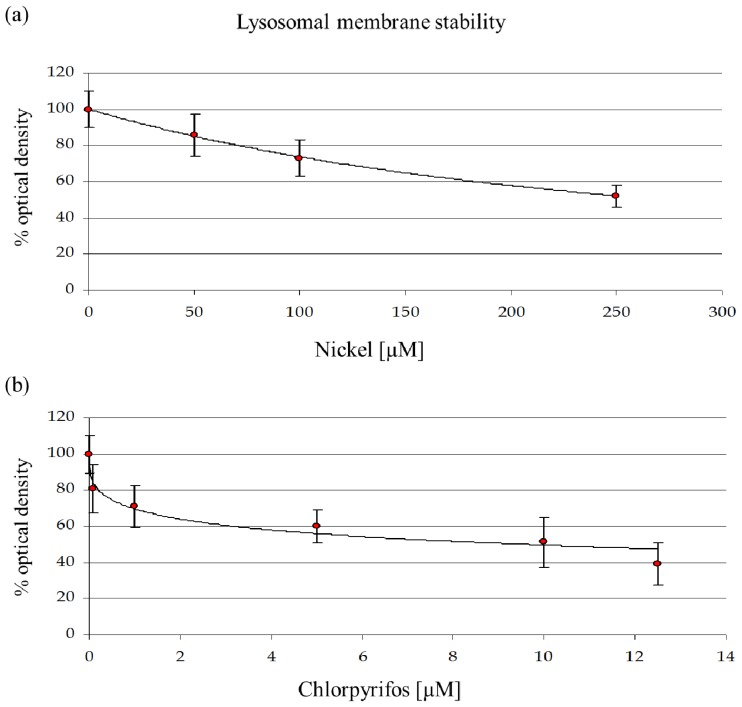
Effects of nickel (**a**) and chlorpyrifos (**b**) on lysosomal membrane stability in *D. discoideum* cells.

After estimating the LOEC concentrations on *D. discoideum*, we used the parameter LMS to calculate toxicity endpoints (EC value) through a log-logistic regression, as described previously [25]. The destabilization of lysosomal membranes represents one of the most sensitive stress response markers in eukaryotic cells, one that is often associated with increased autophagy, catabolism of macromolecules, and cellular injury [26,27]. LMS was used to assess the toxicity of single compound treatments as well as their mixture. From the data plotted in Figure 2, we inferred two equitoxic doses for the single chemicals (EC25 and EC50) and one mixture at a nominal value of 1.0 TU, corresponding to EC50. According to the concentration addiction (CA) model [28], the toxic unit of the mixture is obtained through the combination of NiCl_2_ EC25 and CHP EC25.

#### 2.1.3. Endocytic Rate

**Figure 3:**
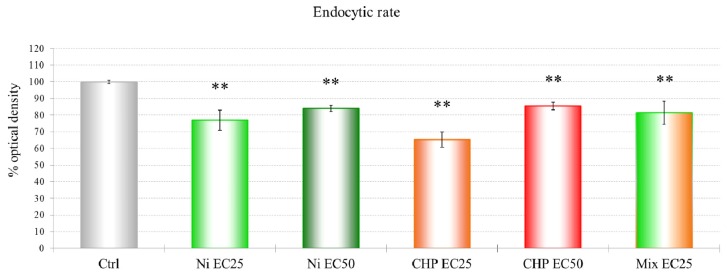
Changes in the endocytic rate of *D. discoideum* due to the various compound treatments. A statistical significance of *p* ≤ 0.01 is indicated as ** (Mann-Whitney test).

Treatments of the single compounds at EC25 and EC50 and their mixture result in a negative effect on the rate of endocytosis of *D. discoideum* (Figure 3).

### 2.2. Effects of the Toxicants on the *D. discoideum* Proteome

#### 2.2.1. Nickel

**Figure 4:**
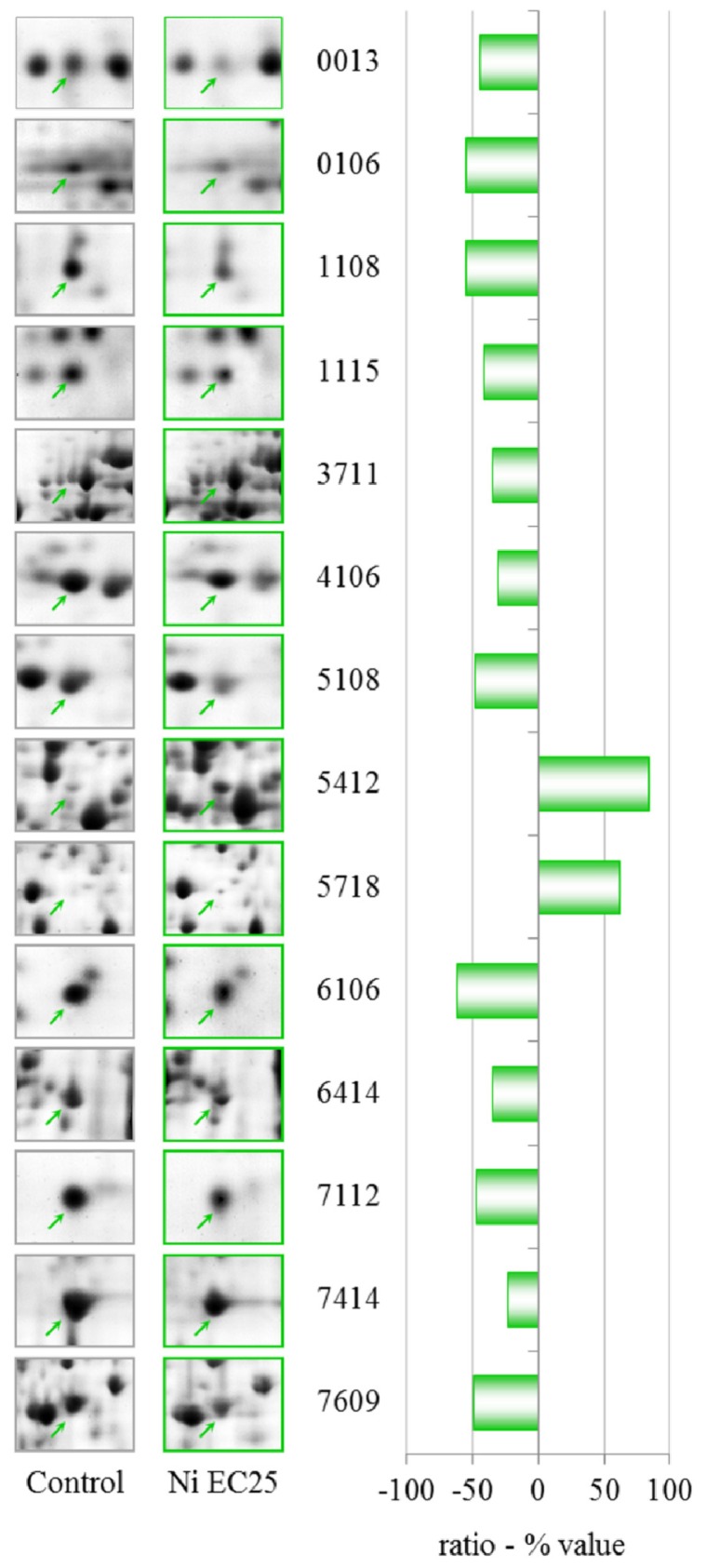
Two-dimensional gel electrophoresis (2-DE) gel portion of protein spots that are differentially expressed after treatment with 114.6 μM NiCl_2_ (EC25). The graph shows the ratio (% value) obtained by the PDQuest software (*p* ≤ 0.05, derived from the Mann-Whitney U-test). Protein identification details can be found in Table 1.

Treatment with 114.6 μM NiCl_2_ (EC25) induced changes in 14 protein spots, two of which were up-regulated (Figure 4, Table 1). One down-regulated spot contained a mixture of dihydropteridine reductase, peroxiredoxin and an unknown protein, which prevents any quantitative evaluation and will not be discussed further.

**Figure 5:**
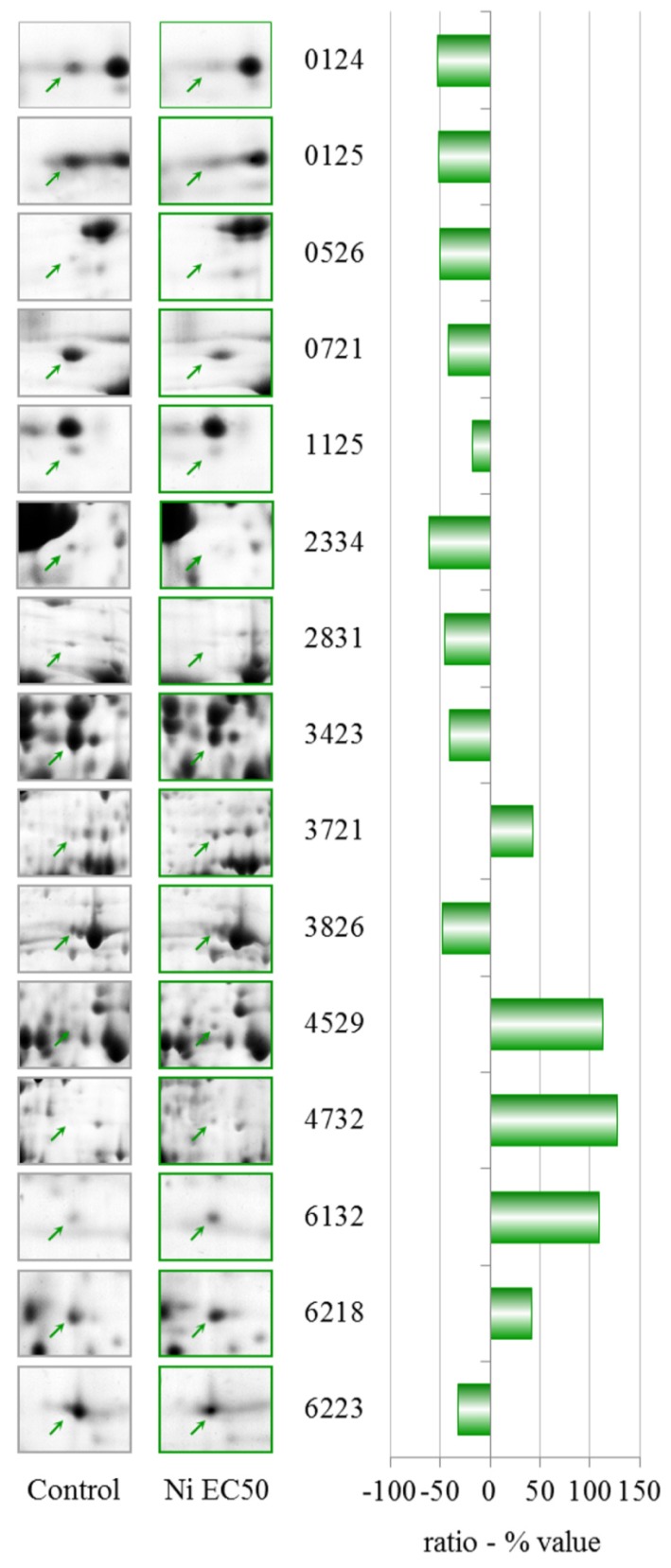
2-DE gel portion of protein spots that were differentially expressed after treatment with 249.5 μM NiCl_2_ (EC50). The graph shows the ratio (% value) obtained using the PDQuest software (*p* ≤ 0.05, derived from the Mann-Whitney U-test). Protein identification details can be found in Table 2.

NiCl_2_ treatment at EC50 (249.5 μM) showed a completely different PES with respect to the PES observed after the EC25 treatment, and the proteins with altered levels after the EC25 treatment returned to control levels after the EC50 treatment, showing a non-linear dose-response in protein levels. After treatment with NiCl_2_ at EC50, we observed the differential expression of 15 spots, five of which were up-regulated (Table 2, Figure 5).

For one up-regulated spot that contained a combination of glutamate-ammonia ligase and prolyl oligopeptidase and two down-regulated spots that contained either *N*-acyl-l-amino-acid amidohydrolase and 26S proteasome non-ATPase regulatory subunit 6 or RepC-binding protein A and calreticulin, the quantitative changes in individual protein levels cannot be assigned to single proteins; therefore, they will not be discussed further.

#### 2.2.2. Chlorpyrifos

**Figure 6:**
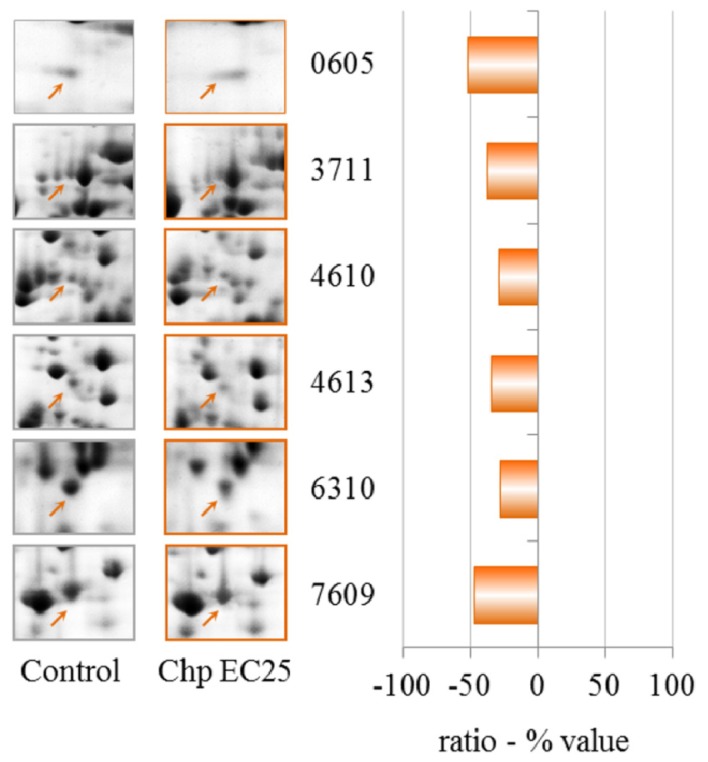
2-DE gel portion of protein spots that were differentially expressed after treatment with 0.7 μM CHP (EC25). The graph shows the ratio (% value) obtained by the PDQuest software (*p* ≤ 0.05, derived from the Mann-Whitney U-test). Protein identification details can be found in Table 3.

The exposure of *D. discoideum* cells to 0.7 μM CHP (EC25) induced the down-regulation of six spots, as shown in Table 3 and Figure 6. Interestingly, cyclase-associated protein and glutamate ammonia ligase were also down-regulated by NiCl_2_ at the same equitoxic concentration (EC25).

**Figure 7:**
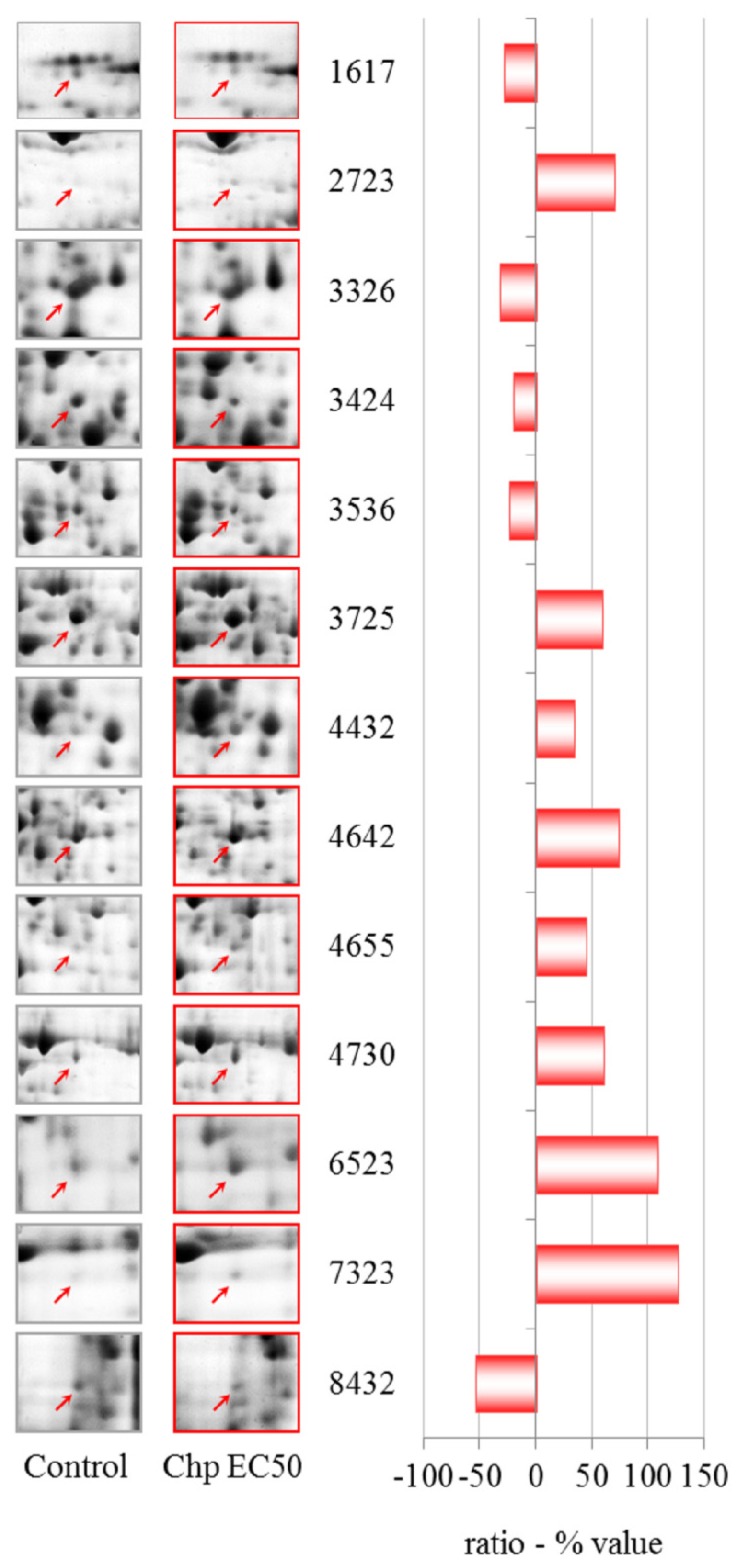
2-DE gel portion of protein spots that were differentially expressed after treatment with 9.6 μM CHP (EC50). The graph shows the ratio (% value) obtained by the PDQuest software (*p* ≤ 0.05, derived from the Mann-Whitney U-test). Protein identification details can be found in Table 4.

The proteomic analysis after treatment with CHP at EC50 (9.6 μM) (Table 4, Figure 7) revealed that additional and different proteins were modulated with respect to the EC25 treatment. Only the down-regulation of aldehyde dehydrogenase was common to the PESs of the two treatments, and there was a non-linear trend in the proteomics data for this compound. One spot appeared to be up-regulated, but after it was analyzed several times by mass spectrometry, we could not identify its constituent protein. The spot containing succinate dehydrogenase also contained an unknown protein that is likely to hamper any quantitative evaluation.

#### 2.2.3. Mixture

**Figure 8:**
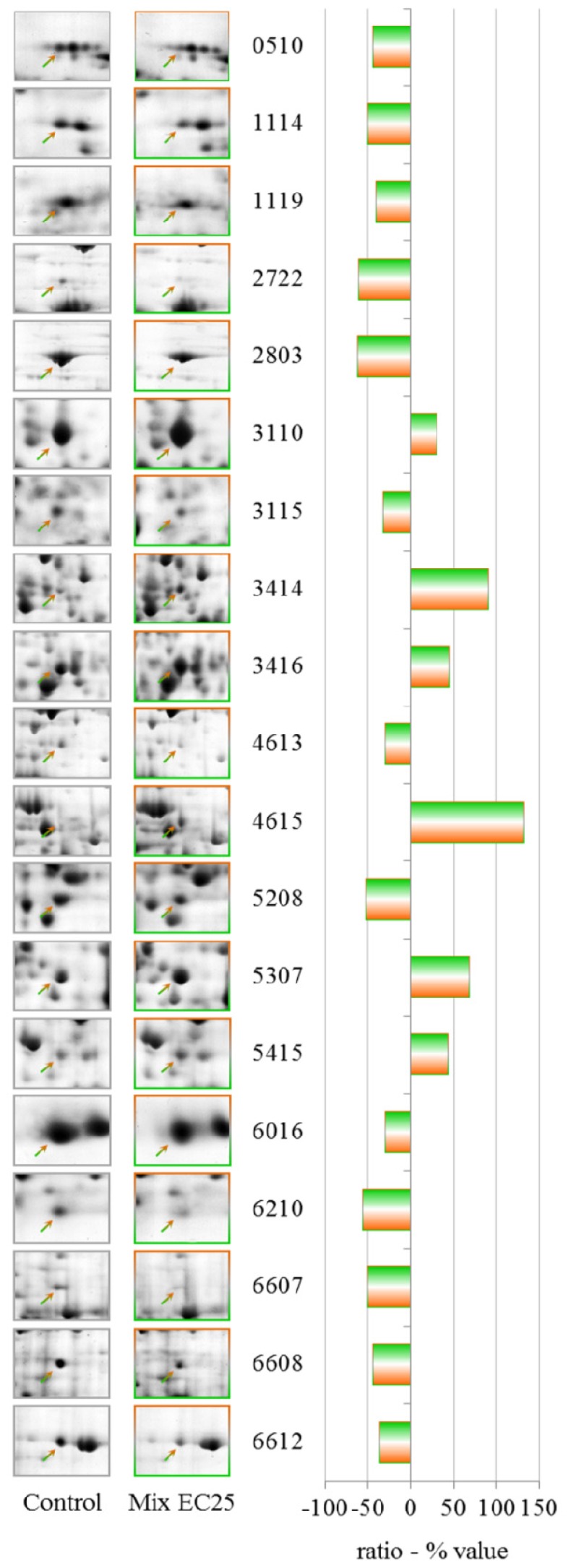
2-DE gel portion of protein spots that were differentially expressed after treatment with the mixture (NiCl_2_ EC25 + CHP EC25). The graph shows the ratio (% value) obtained by the PDQuest software (*p* ≤ 0.05, derived from the Mann-Whitney U-test). Protein identification details can be found in Table 5.

The effects on the proteome after treatment with the equitoxic mixture indicated some overlap in the PES analyses for the single compounds for different proteins (Table 5, Figure 8). In Table 6, we reported the common proteins that change in some of the various treatments but with different trends. Interestingly, the levels of two different isoforms of *S*-adenosyl-methionine synthetase change in different treatments. The spots that changed in density after treatment with the mixture did not show comigrating proteins; in one case, we were unable to identify a down-regulated protein.

## 3. Discussion

The mortality and lysosomal membrane destabilization caused by CHP and NiCl_2_ treatments in amoebae were evaluated to determine appropriate concentrations of both toxicants for further analyses. To evaluate the effects on mortality, various concentrations of CHP (0–50 μM) and NiCl_2_ (0–10 mM) were tested. LMS data were evaluated for CHP from 0 to 12.5 μM and for NiCl_2_ form 0 to 250 μM, which allowed us to evaluate the sublethal treatments that induce toxic or adaptive effects. For the single compounds, two experimental levels based on LMS data were tested and represented realistic (NiCl_2_ 114.6 μM; CHP 0.7 μM) and extreme (NiCl_2_ 249.5 μM; CHP 9.6 μM) scenarios. Moreover, a mixture of the two substances at equitoxic concentrations was assessed to obtain information about their interaction at the physiological and molecular levels.

The differentially expressed proteins were analyzed by Blast2GO for molecular processes and biological functions and the results can be found as Supplemental File (Blast2GO Analysis).

### 3.1. Nickel

NiCl_2_ is not extremely toxic to *D. discoideum*, probably because of an energy-dependent nickel export that exists in wild-type and mutant strains, but its role in heavy metal resistance has not been confirmed [29]. The mortality rate and lysosomal membrane destabilization data (Figures 1 and 3) revealed that the cytotoxicity increased with an increase in the NiCl_2_ concentration. Although the first effects on mortality were observed only after treatment with 0.75 mM NiCl_2_, at the subcellular level, LMS and proteomic data show effects at 10% of that concentration.

A concentration of 114.6 μM NiCl_2_, which corresponds to an EC25 for lysosomal membrane destabilization, was chosen for subsequent experiments. The sublethal effects at this concentration were evident, which might indicate that differential protein expression already occurs at this concentration. Moreover, after the sublethal treatments, amoebae could maintain a normal phenotype, which allowed us to extract sufficient amounts of proteins for 2DE experiments. A more toxic concentration of 249.5 μM of NiCl_2_, corresponding to EC50 for LSM, was chosen for comparison.

The data in Table 1 show that clear effects on the proteome can be observed after treatment with NiCl_2_ at EC25. Two proteins were up-regulated, succinate dehydrogenase and an isoform of *S*-adenosylmethionine synthetase (gi|66803080). The activity of succinate dehydrogenase has been previously observed to be inhibited by cadmium, with the possibility of interfering with energy transport [30]. *S*-Adenosylmethionine synthetase (SAM-S) catalyzes the biosynthesis of S-adenosylmethionine from Met and ATP. In plants, the expression of SAM-S is significantly altered in response to salt and drought [31,32]. In yeast, the activity of this enzyme is strongly inhibited by heavy metal ions (Cu^2+^ and Ag^2+^) and ethylenediaminetetraacetic acid (EDTA). Moreover, the expression levels of SAM-S were markedly up-regulated in response to arsenate in rice roots [33]. Unexpectedly, a different isoform of *S*-adenosyl-methionine synthetase (gi|60463691) was down-regulated.

Cyclase-associated proteins (CAPs) are widely distributed and highly conserved proteins that regulate actin remodeling, vesicle trafficking and development in response to cellular signals [34]. In *D. discoideum*, CAPs are essential for the functions of the endo-lysosomal system [35]. This protein was down-regulated after treatment with CHP at EC25, and both NiCl_2_ and CHP EC25 treatments had the clearest effects on endocytosis. The effects on endocytosis are probably linked to the down-regulation of a subunit of the actin-related protein 2/3 (ARP2/3) complex, which plays a major role in actin cytoskeletal regulation.

Glutamate-ammonia ligase was recently identified as a nickel-binding protein by mass spectrometry, and its activity is inhibited by nickel [36].

Rho GDP dissociation inhibitor (RhoGDI) plays an essential role in the control of a variety of cellular functions through its interactions with Rho family GTPases. RhoGDI is frequently over-expressed in human tumors and chemo-resistant cancer cell lines [37]. The observed down-regulation due to NiCl_2_ could be of great interest and should be studied further.

The down-regulation of LIM-type zinc finger-containing protein after NiCl_2_ treatment is not surprising because low concentrations of transition metal have been shown to interfere with DNA transcription and repair. Various effects of heavy metals on zinc finger-containing proteins have been identified, including the displacement of zinc by cadmium or cobalt, the formation of mixed complexes, incomplete coordination of toxic metal ions, and the oxidation of cysteine residues within the metal-binding domain [38].

The down-regulation of 60S acidic ribosomal protein P2, a ribosomal protein-like and elongation factor 1β, suggests a modulation in protein synthesis caused by the presence of nickel.

After comparing the results of the cytotoxicity tests and protein expression data, it can be seen that when the cells were treated with NiCl_2_ concentrations ranging from EC25 to EC50, the cytotoxic effects were linear, but different altered proteins were identified (Tables 1 and 2). These data indicate that NiCl_2_ toxicity cannot be quantitatively assessed by following the expression of single proteins in such a wide concentration range.

NiCl_2_ at EC50 induces different patterns of up-regulation, including an increase in the level of peroxiredoxin. The enzymes in this family consist of sulfhydryl-dependent peroxidases that exhibit high reactivity with hydrogen peroxide and play major roles in peroxide defense, redox cell signaling and metal responses [39,40]. Peroxiredoxin has recently been found to have an increased expression in nickel-exposed workers [41]. Nickel causes increased levels of endogenous cellular hydrogen peroxide and its short-lived reactive oxygen species [42–44].

Up-regulation of actin binding protein along with a modulation of the ARP2/3 complex and CAPs substantiates the involvement of the cytoskeleton in the cellular response to nickel. This up-regulation of cytoskeletal proteins in the EC50 treatment could explain the partial recovery of endocytic function. Accordingly, the up-regulation of AAA-ATPase domain-containing protein and a putative epimerase should be investigated further. There is little information about these two proteins, but a member of the AAA-ATPase family has recently been described as a novel anti-apoptotic factor in lung adenocarcinoma cells [45].

We observed the down-regulation of different proteins after treatment with NiCl_2_ at EC50. Major vault protein (MVP), which is the main component of the vault complex, is over-expressed in many multidrug-resistant cancer cell lines, suggesting a possible role for MVP in cell signaling and survival [46,47]. Vault complexes are implicated in the cellular response to environmental toxins, but we observed a reduction in the amount of this protein; thus, it is possible that NiCl_2_ does not activate this response pathway.

Glucose-regulated protein 94 is a member of the endoplasmic reticulum (ER) chaperone family. In *D. discoideum*, it is a constitutively expressed protein that is over-expressed in certain abnormal cellular conditions, including the depletion of glucose and calcium, low oxygen levels and low pH [48]. We observed down-regulation of this protein after treatment with NiCl_2_ at EC50.

We observed the down-regulation of phosphoglycerate kinase after treatment with NiCl_2_ at EC50. The role of phosphoglycerate kinase in glycolysis is very important for the production of ATP through substrate level phosphorylation. Regulation of this protein is thereby controlled by the energy level of the cell.

Phosphoribosyl pyrophosphate synthetase (PRPS), which was also down-regulated, plays a key role in the biosynthesis of several amino acids and pyrimidines. PRPS is required to convert ribose 5-phosphate into phosphoribosyl pyrophosphate. The latter is a central compound for cellular metabolism and may be considered as a link between carbon and nitrogen metabolism.

NiCl_2_ induces the down-regulation of the cytosolic glycoprotein FP21 of *D. discoideum*, which is an orthologue of human and mouse *S*-phase kinase-associated protein 1. This protein is an essential component of the SCF (SKP1-CUL1-F-box protein) ubiquitin ligase complex, which mediates the ubiquitination of proteins involved in cell cycle progression, signal transduction and transcription.

We observed the down-regulation of the nascent polypeptide-associated complex alpha subunit. This protein prevents short, recently synthesized ribosome-associated polypeptides from inappropriate interactions with cytosolic proteins.

### 3.2. Chlorpyrifos

The data show a toxic effect of CHP in *D. discoideum*, an organism that possesses no nervous system. CHP at 9.6 μM already induces the first lethal effects and 50% lysosomal membrane destabilization. Moreover, this compound exerts negative effects on endocytosis.

The proteomic data reveal that CHP at EC25 induces the down-regulation but not up-regulation of proteins. Cyclase-associated proteins are multifunctional proteins that contain several structural domains. Recent studies have suggested important roles for these proteins in linking cell signaling with actin polymerization and highlight their roles in vesicle trafficking and development, as discussed for NiCl_2_ at EC25.

We observed the down-regulation of the 26S proteasome ATPase 1 subunit. The ubiquitin/26S proteasome pathway is implicated in numerous diseases, including cancer and neurodegenerative diseases [49]. The 26S proteasome regulatory complex has intrinsic ATPase activity that seems to play an essential role in its function. Presumably, one role of the ATPase is to supply energy continuously for the selective degradation of target proteins by the active 26S proteasome.

In bacteria, yeast and plants, glutamate-ammonia ligase (glutamine synthetase) expression is tightly regulated by the metabolic status of the cell [50]. A previous study in freshwater crabs showed that glutamine synthetase activity increased after long-term organophosphate exposure [51]. We observed the down-regulation of this enzyme, indicating that this protein is involved in the response to acute exposure to CHP in *D. discoideum*.

Isocitrate dehydrogenase (ICDH) catalyzes the oxidative decarboxylation of isocitrate. Eukaryotes possess two distinct types of ICDH: an NAD-specific enzyme (EC 1.1.1.41), which is present only in the mitochondria, and an NADP-specific enzyme (EC 1.1.1.42), which is found in both the cytoplasm and the mitochondria. The reduced activity of liver mitochondrial ICDH has been correlated with organophosphate toxicity [52,53]. There is also evidence correlating the activities of ICDH and the maintenance of the cellular redox state, suggesting that ICDH plays an important role in cellular defenses against oxidative stress [54].

Aldehyde dehydrogenase catalyzes the oxidation of various aldehydes, using NAD+ as a cofactor [55]. The enzyme is inhibited by dithiocarbamate fungicides and has previously been described as a suitable detector for these pesticides in the environment [56,57]. This enzyme is down-regulated by treatment with CHP at EC25.

Increased transcription of ZPR1-type zinc finger-containing protein has been observed in potato tuber periderm after heat stress [58]. The deficiency of the zinc finger protein ZPR1 causes neuro-degeneration and defects in transcription in the mouse [59] and cell cycle progression in HeLa cells [60]. After treatment with CHP at EC25, we observed the down-regulation of this protein.

The EC50 treatment induces variations in the levels of different protein spots; two of these spots, which were up-regulated, contained two comigrating proteins. In one spot, we identified a tetratricopeptide-like helical domain-containing protein and stress-induced phosphoprotein 1. The other spot contained succinate dehydrogenase and an unknown protein. These spots, which contained comigrating proteins, give no quantitative information because it is impossible to establish which individual protein has altered levels.

The only protein that was down-regulated in both CHP treatments was aldehyde dehydrogenase, which is expected because this protein was previously found to act as a detector of pesticides in the environment [56,57].

Among the up-regulated proteins, there is a putative iron regulatory protein (IRP) that acts as a key regulator of cellular iron homoeostasis as a result of the translational control of the expression of a number of iron metabolism-related genes. It is likely that various agents and conditions affect IRP activity, thereby modulating iron and oxygen radical levels in different circumstances [61].

Previous studies have demonstrated that the up-regulation of transketolase (TKT) might play a critical role in maintaining a reducing environment in the mouse cornea [62]. Moreover, in the cyanobacterium Anabaena, UV-B upregulated transketolase and protein down-regulation were observed with Cd treatment compared to a control treatment [63]. The up-regulation that was observed in the present study suggests a role for this protein in the response to CHP.

Another protein that was up-regulated by CHP at EC 50 is annexin VII. Annexins belong to a large family of glycoproteins that bind both Ca^2+^ and negatively charged phospholipids and are considered important components of calcium signaling pathways [64,65]. Various studies have shown that annexins play important roles in the cell during the response to oxidative stress [66–68]. The peroxidase activity that is exhibited by annexins is further exemplified by studies in plants [69,70].

β-alanine synthase is an enzyme that catalyzes the final step of pyrimidine catabolism and is required for the biosynthesis of β-alanine in animals. In mammals, β-alanine is a natural antioxidant that is produced by the degradation of uracil or carnosin, which is a polypeptide that is formed by β-alanine and l-lysine. Moreover, β-alanine is necessary for the production of CoA, which is required for fatty acid metabolism. The treatment of *D. discoideum* cells with 9.6 μM CHP induced an increase in ROS production (Figure S1) with a simultaneous up-regulation of β-alanine synthase, which is the enzyme required for the production of β-alanine. Moreover, as described previously, we found the down-regulation of aldehyde dehydrogenase and *S*-adenosylmethionine synthetase (SAMS), both of which are needed in other mechanisms of β-alanine metabolism. We could hypothesize that the attempt by *D. discoideum* to modulate a specific pathway for β-alanine production is probably an alternative defense mechanism in response to the oxidative stress that is induced by pesticide treatment.

The expression levels of *S*-adenosylmethionine synthetase (SAMS) and a SAM-dependent methyltransferase are markedly up-regulated in response to arsenate in plants [33]. Conversely, we observed the down-regulation of these proteins after CHP treatment at EC50. This difference requires further investigation.

We observed an increase in phosphopyruvate hydratase (also known as enolase), which is a metalloenzyme that is required for the penultimate step of glycolysis. Increased expression of enolase has recently been reported in stressed plants [71].

The 60S ribosomal protein L4 is a component of the large ribosomal subunit. Yang *et al.* (2005) have show that this protein is required for rRNA processing in mammalian cells, and that decreasing the level of this peptide inhibits the production of several rRNAs [72].

The exact physiological role of protein phosphatase 2C-like domain containing protein, which we found to be down-regulated after CHP at EC50 treatment, is unclear.

### 3.3. Mixture

The results have demonstrated that each treatment results in different PESs, and the mixture gives a unique PES, making it difficult to apply PES to the toxicological evaluation of complex environmental matrixes. However, the data show that some proteins that have altered levels after the single toxicant treatments are involved in the cellular response to the mixture. The prominent protein in this class is cyclase-associated protein, which is down-regulated in both single treatments at EC25. Aldehyde dehydrogenase is down-regulated after treatment with CHP at EC25 but up-regulated by treatment with the mixture, along with aldehyde reductase, indicating an attempt by the cell to counteract the formation of lipid peroxide-derived aldehydes that are produced downstream of ROS [73].

The down-regulation of G β-like protein indicates an involvement of G proteins in the response to the mixture. G proteins represent one of the most prevalent signaling systems in mammalian cells, regulating systems as diverse as sensory perception, cell growth and hormonal regulation.

Phosphoglycerate kinase catalyzes the reversible transfer of a phosphate group from ATP to 3-phosphoglycerate, producing ADP and 1,3-bisphosphoglycerate. This protein is down-regulated by CHP at EC50 but is up-regulated by the mixture.

*S*-adenosyl-methionine synthetase levels are altered by both NiCl_2_ EC25 and CHP EC50, and the protein was strongly up-regulated after treatment with the mixture; a putative δ-24-sterol methyltransferase that uses *S*-adenosyl-methionine as substrate was also up-regulated by the mixture.

Different cytoskeletal proteins, including actin binding protein, myosin II heavy chain, and clathrin heavy chain, were down-regulated by the mixtures, indicating the involvement of the cytoskeleton in the response to toxic substances, despite the fact that treatment with the mixture showed a partial recovery of the endocytic rate.

Isocitrate dehydrogenase (NAD+) levels were down-regulated by the mixture and by CHP at EC25. Aconitase (mitochondrial) has been established as a sensitive target of ROS, and the levels of this enzyme were down-regulated after treatment with the mixture.

ATP citrate synthase catalyzes the first reaction in the Krebs cycle, the conversion of oxaloacetate and acetyl-coenzyme A into citrate and coenzyme A. This reaction is important for energy generation and for carbon assimilation and was down-regulated by the mixture.

We observed the up-regulation of 3-phosphoglycerate dehydrogenase, which catalyzes the transition of 3-phosphoglycerate to 3-phosphohydroxypyruvate. This reaction is the first and rate-limiting step in the phosphorylated pathway of serine biosynthesis; serine is a constituent of proteins and a precursor of several metabolites, including cysteine, glycine and choline.

We observed the down-regulation of the pyruvate dehydrogenase E1 beta subunit. The pyruvate dehydrogenase complex is a nuclear-encoded mitochondrial multi-enzyme complex that catalyzes the overall conversion of pyruvate to acetyl-CoA and CO_2_ and provides a primary link between glycolysis and the tricarboxylic acid (TCA) cycle.

Another down-regulated protein was glucosamine-6-phosphate isomerase, which catalyzes the conversion of d-glucosamine 6-phosphate (GlcN6P) to d-fructose 6-phosphate and ammonia. This reaction is the last step in the pathway for *N*-acetylglucosamine utilization in bacteria or fungi.

Another interesting protein that was strongly up-regulated by the mixture was a putative transport protein that should be studied further to clarify its involvement in the response to the toxic mixture.

Based on these results, it is possible to investigate the molecular mechanisms of cytotoxicity on a proteomic scale by using a proteomic approach, despite the fact that each treatment has a different PES. In particular, this approach, beyond the simultaneous quantitative observation of proteins that are known to be stress responsive in a single experiment, allows us to identify novel proteins that have never previously been observed to change in level after toxic treatments.

## 4. Experimental Section

### 4.1. *Dictyostelium* Growth and Treatments

The social amoeba *Dictyostelium discoideum* (strain AX-2) was grown axenically in AX-2 medium as described previously [74]. The cells were grown under laboratory conditions on a Gallenkamp orbital incubator (Sanyo Gallenkamp, Plc. Loughborough, UK) at 150 rpm and at 21 °C [75]. To synchronize the cell cycle, the amoebae in logarithmic phase (2–4 × 10^6^ cells/mL) were stored overnight at 4 °C [76]. The cell treatment was performed in diluted medium for 6 h as described previously [77]. Chemicals of analytical grade were purchased from Sigma-Aldrich if not stated otherwise.

Nickel was administered as a chloride salt (NiCl_2_) from a concentrated stock solution (1000×), CHP was diluted in dimethylsulphoxide (DMSO) and added at the desired concentration from a concentrated stock solution (1000×). To exclude any effects of the DMSO vehicle, the same solvent volume was added to reference cells and NiCl_2_-exposed cells at the same dilution.

To evaluate the first significant effects on mortality in *D. discoideum* (LOEC), the cells were exposed to increasing doses of NiCl_2_ (0–10.0 mM) and CHP (0–50 μM).

After estimating the LOEC concentrations on the *D. discoideum* cells, we exposed the cells to increasing doses of NiCl_2_ (0–250 μM) and CHP (0–12.5 μM) to calculate toxicity endpoints (EC) using the parameter of lysosomal membrane stability (LMS).

For the proteomics studies and endocytic rate studies, *D. discoideum* cells were treated with two different equitoxic doses of the single chemicals—0.5 (EC25) and 1.0 (EC50) TU, and one mixture at a nominal 1.0 TU. According to the CA model [28], these toxic levels are obtained through the combination of 2 × 0.5 TU. The cells were exposed to 114.6 and 249.5 μM NiCl_2_ and 0.7 and 9.6 μM CHP, which correspond to EC25 and EC50, respectively.

### 4.2. Mortality Rate, Lysosomal Membrane Stability and Endocytic Rate

Biomarker assays were performed as described previously [77]. Briefly, the mortality rate was assessed using the DNA-binding dye SYBR Green™ (Sigma-Aldrich, Milan, Italy), and observations of the cells were performed at 460× magnification on a Zeiss Axiovert 100M. For the evaluation of LMS, the retention times of neutral red (NRRT) dye in the lysosomes after exposure to several doses of pollutants were monitored.

Slides were observed at 1449× magnification in an inverted camera-equipped microscope (Zeiss Axiovert 100 M) that was controlled remotely via AxioVision software (Zeiss, Germany). For the endocytic rate, an aliquot of 40 μL cell suspension was incubated for 15 min with Fluorescent Bioparticles (*Escherichia coli* K-12 strain; Abs/Em maxim = 505/513 nm; Molecular Probes, Eugene, US). After incubation, *D. discoideum* cells were fixed in 3.7% paraformaldehyde and observed with a microscope at 1.449× magnification.

The images that were obtained from LMS and endocytosis were analyzed using an image analysis system (Scion Image freeware v 1.62) (Scion Corp., Frederick, MD, USA) that allowed the quantification of lysosomal Neutral Red (NR) and the quantification of fluorescent bacteria in the cells. The data were expressed as a percentage of the optical density with respect to the control condition. Data analysis was conducted using Winks SDA (ver. 6.0.5) statistics software (Texasoft, Duncanville, TX, USA). A non-parametric Mann-Whitney test was used to compare the control and treated samples. A statistical significance of *p* ≤ 0.05 is indicated as *, and a statistical significance of *p* ≤ 0.01 is indicated as **.

### 4.3. Cell Lysis and Protein Extraction

After treatments with several doses of nickel, CHP and their mixture, *D. discoideum* at a culture concentration of 45 × 10^6^ cells were washed twice with PAS, centrifuged at 500× *g*, precipitated and lysed in buffer (0.3% SDS, 200 mM DTT, 30 mM Tris, Protease Inhibitor Cocktail, P2714, Sigma-Aldrich, St Louis, MO, USAS) as described previously [74]. Protein precipitation was performed in 80% (*v*/*v*) cold acetone in MilliQ water and centrifuged at 5000× *g* for 10 min.

After discarding the supernatant, the protein pellet was solubilized in a typical buffer for first-dimension isoelectric focusing (IEF) (7 M urea, 2 M thiourea, 4% *w*/*v* CHAPS, and 50 mM DTT).

### 4.4. 2-DE Gel Electrophoresis

The proteins were extracted from seven biological replicates for each treatment and were separated by 2-DE gel electrophoresis (2-DE) according to a method described previously [74].

For IEF, immobilized strip gels (pH 3–10 linear, 13 cm) (GE Healthcare Europe GmbH) were used to separate 800 μg of proteins. IEF was conducted at 20 °C on an IPGphor unit (GE Healthcare Europe GmbH).

For the second dimension, the focused proteins on the strips were reduced in an SDS equilibration buffer (50 mM Tris-HCl pH 8.8, 6 M Urea, 30% (*v*/*v*) glycerol, 2% (*w*/*v*) SDS) containing 10 mg/mL DTT for 15 min and subsequently alkylated with 25 mg/mL iodoacetamide for 10 min.

SDS-PAGE on 12% gels was performed in a Protean II XL 2-D cell (Bio-Rad Laboratories). The gels were fixed in a solution containing 40% (*v*/*v*) methanol and 10% (*v*/*v*) acetic acid in MilliQ water and stained with Colloidal Coomassie brilliant blue G250 (Bio-Rad Laboratories) according to Neuhoff *et al.*[78]. Excess stain was removed by rinsing the gels with a solution containing methanol and acetic acid in MilliQ water.

### 4.5. Image Analysis

The gels (3–10 pH) were scanned using a densitometer GS-710 (Bio-Rad Laboratories) and analyzed using PDQuestTM Software version 7.3.1 (Bio-Rad Laboratories), following the typical analysis workflow. The PDQuest matching summary is provided in Supplemental Table 2, and a typical 2DE map is presented in Supplemental Figure 2.

The relative abundance of each spot in the control and treated gels, obtained from seven biological replicates for each treatment, was performed by a software program using the Mann-Whitney U-test, and the differences were considered significant at *p* ≤ 0.05.

### 4.6. Automated ProteinSspot Picking and In-Gel Digestion

The differentially expressed protein spots were transferred from gels to 96-well plates using a Proteome Works Spot Cutter (Bio-Rad Laboratories) (Richmond, CA, USA) that was equipped with a 1 mm diameter needle. For high-throughput identification by nanoHPLC-ESI-QTOF, the proteins were digested by trypsin (Roche Diagnostics, Monza, Italy) and extracted with Multiprobe II (Perkin Elmer, Wellesley, MA, USA) as described previously [74].

### 4.7. Protein Identification by Nano-LC/Q-TOF MS/MS

The peptides samples that were obtained by in-gel digestion were vacuum dried and stored at −20°C until nano-high performance liquid chromatography (HPLC) ESI-Q-TOF MS/MS analysis.

Mascot.dll v 1.4804.0.22 (Matrix Science/AB Sciex) was used to generate Mascot (.mgf) files with peak lists from the Analyst QS 2.0 (.wiff) files; the default parameters were used.

The MS/MS spectra that were obtained by spot samples were analyzed as Mascot generic files against all entries in the public NCBInr database using the online Mascot program [79] without a taxonomy filter.

The principal parameter settings for the Mascot search were as follows: database NCBInr (version 2012.04.07; 17751536 sequences) [80]; enzyme trypsin; allow up to one missed cleavage; variable modifications carbamidomethyl (C), oxidation (M), deamidated (NQ); tolerances peptide ± 0.25 Da and MS/MS ± 0.25 Da; instrument ESI-QUAD-TOF; default charge state was set to 2+, 3+, and 4+. Only proteins with at least two peptides identified with significant ion scores (*p* ≤ 0.05) were considered as identified. Protein identification details are shown in Table S1.

## 5. Conclusions

A large number of studies have focused on acute pollutant toxicity [81]. The ability of *D. discoideum* to eliminate copper ions has been described previously [82]. *D. discoideum* has been proposed as a model organism in toxicology [83]. However, there is little information about the effects of toxic mixtures on the *D. discoideum* proteome.

We elucidated the proteomic responses of *D. discoideum* that were exposed to NiCl_2_, CHP and their mixture. It is likely that the direct use of PES requires much more data and more sensitive techniques to resolve the molecular mechanisms of the cellular responses to be routinely applicable to toxicity evaluations in environmental matrixes. There are obvious difficulties in understanding the biological complexity of the molecular mechanisms of the cell, and the technical procedures of the present study could be used to study the interactions between other mixtures and in other organisms. They could also be used to develop new methods to evaluate the toxicity of individual toxicants and their mixtures. The comparisons of the protein fingerprints of an equitoxic binary mixture and its single constituent compounds allows us to individuate additional molecular mechanisms that are switched on, which can be studied in the future with more specific approaches (e.g., the involvement of the cytoskeleton in toxic responses). Our study not only detected known pollutant-modulated proteins such as peroxiredoxins, aldehyde reductase and dehydrogenase, and *S*-adenosyl-methionine synthetase, which were observed previously [84–86], but also revealed significant changes in the levels of unknown proteins, such as a putative transport protein (gi|166240434), that were strongly induced by the mixture but were never previously studied during a toxic response.

## Supplementary Information











## Figures and Tables

**Table 1:** Nickel EC25—identities of proteins with altered levels. Additional data can be found in Table S1.

Spot n°	Protein ID	gi	DDB	Ratio (% value)
0013	60S acidic ribosomal protein P2	gi|133059	DDB0191484	−45.3
0106	LIM-type zinc finger-containing protein	gi|7107412	DDB0201567	−55.7
1108	Rho GDP-dissociation inhibitor	gi|66803106	DDB0216235	−55.5
1115	Actin-related protein 2/3 complex, subunit 5	gi|66806101	DDB0191138	−41.5
3711	Glutamate-ammonia ligase	gi|60469746	DDB0231551	−35.6
4106	Peroxiredoxin	gi|28828367	DDB0231647	−31.2
	Dihydropteridine reductase	gi|66823097	DDB0237752	
5108	Hypothetical protein DDBDRAFT_0206195	gi|66815899	DDB0206195	−48.5
	Peroxiredoxin	gi|66821043	DDB0231647	
5412	*S*-adenosylmethionine synthetase	gi|66803080	DDB0230070	83.8
5718	Succinate dehydrogenase	gi|66813780	DDB0214886	61.2
6106	Similar to ribosomal protein	gi|66821505	DDB0167610	−62.4
6414	*S*-adenosyl-methionine synthetase	gi|60463691	DDB0230070	−35.4
7112	Hypothetical protein DDB_0233902	gi|60475381	DDB0233902	−47.7
7414	Elongation factor 1 β	gi|10801150	DDB0191174	−24.0
7609	Cyclase-associated protein	gi|66805581	DDB0191139	−49.5

**Table 2:** Nickel EC50—identities of proteins with altered levels. Additional data can be found in Table S1.

Spot n°	Protein ID	gi	DDB	Ratio (% value)
0124	Putative polypeptide-associated complex alpha subunit	gi|66812950	DDB0233328	−52.8
0125	Cytosolic glycoprotein FP21	gi|66826197	DDB0191107	−52.0
0526	RepC-binding protein A	gi|4336714	DDB0191177	−50.1
	Calreticulin	gi|66810606	DDB0191384	
0721	Glucose-regulated protein 94	gi|66814268	DDB0215015	−42.0
1125	Nascent polypeptide-associated complex alpha subunit	gi|66812950	DDB0233328	−18.1
2334	*N*-acyl-l-amino-acid amidohydrolase	gi|66825457	DDB0201767	−61.1
	26S proteasome non-ATPase regulatory subunit 6	gi|66826503	DDB0232985	
2831	Hypothetical protein DDBDRAFT_0192196	gi|66799895	DDB0192196	−44.8
3423	Phosphoglycerate kinase	gi|22711882	DDB0191349	−40.4
3721	Glutamate-ammonia ligase	gi|66815105	DDB0231551	42.3
	Prolyl oligopeptidase	gi|4584573	DDB0185041	
3826	Major vault protein	gi|66825911	DDB0191259	−47.3
4529	Actin binding protein	gi|66827977	DDB0191115	112.9
4732	AAA ATPase domain-containing protein	gi|66805423	DDB0231600	127.5
6132	Peroxiredoxin	gi|66808689	DDB0238212	109.2
6218	Putative epimerase	gi|66820416	DDB0167407	41.9
6223	Phosphoribosyl pyrophosphate synthetase	gi|66809487	DDB0237882	−32.1

**Table 3:** Chlorpyrifos EC25—identities of proteins with altered levels. Additional data can be found in Table S1.

Spot n°	Protein ID	gi	DDB	Ratio (% value)
3711	Glutamate-ammonia ligase	gi|60469746	DDB0231551	−52.2
7609	Cyclase-associated protein	gi|66805581	DDB0191139	−37.4
4613	26S proteasome ATPase 1 subunit	gi|66826743	DDB0232964	−28.4
6310	Isocitrate dehydrogenase (NAD^+^)	gi|66799989	DDB0231294	−34.5
4610	Aldehyde dehydrogenase	gi|66818493	DDB0231504	−28.1
0605	ZPR1-type zinc finger-containing protein	gi|66825227	DDB0304584	−47.0

**Table 4:** Chlorpyrifos EC50—identities of proteins with altered levels. Additional data can be found in Table S1.

Spot n°	Protein ID	gi	DDB	Ratio (% value)
1617	Protein phosphatase 2C-like domain-containing protein	gi|66809891	DDB0233767	−29.1
2723	not identified	/	/	69.4
3326	Putative SAM-dependent methyltransferase	gi|60475385	DDB0233903	−32.1
3424	*S*-adenosylmethionine synthetase	gi|66803080	DDB0230070	−20.6
3536	Aldehyde dehydrogenase	gi|66818493	DDB0231504	−24.5
3725	Tetratricopeptide-like helical domain-containing protein (TPR) stress-induced phosphoprotein 1	gi|66801325	DDB0237783	59.1
4432	β-Alanine synthase	gi|66821393	DDB0185221	33.7
4642	Transketolase	gi|66822027	DDB0266926	73.6
4655	Succinate dehydrogenase (ubiquinone)	gi|66813780	DDB0214886	44.7
	Hypothetical protein DDBDRAFT_0218901	gi|66807283	DDB0218901	
4730	Putative iron regulatory protein	gi|66815641	DDB0229908	60.5
6523	Annexin VII	gi|66825303	DDB0191502	108.4
7323	Phosphopyruvate hydratase	gi|66811048	DDB0231355	125.8
8432	60S ribosomal protein L4	gi|66817212	DDB0231241	−54.4

**Table 5:** Mixture EC25—identities of proteins with altered levels. Additional data can be found in Supplemental Table 1.

Spot n°	Protein ID	gi	DDB	Ratio (% value)
0510	Hypothetical protein DDBDRAFT_0187751	gi|66806459	DDB0187751	−44.4
1114	Pyruvate dehydrogenase E1 beta subunit	gi|66818919	DDB0229442	−50.8
1119	Putative delta-24-sterol methyltransferase	gi|60464861	DDB0237965	−41.5
2722	Clathrin heavy chain	gi|66818048	DDB0185029	−60.9
2803	Myosin II heavy chain	gi|134047850	DDB0191444	−62.2
3110	Aldehyde reductase	gi|38637654	DDB0215363	30.1
3115	not identified	/	/	−32.9
3414	Aldehyde dehydrogenase	gi|66818493	DDB0231504	90.3
3416	Phosphoglycerate kinase	gi|22711882	DDB0191349	44.8
4613	Glucosamine-6-phosphate isomerase	gi|66808101	DDB0234127	−30.6
4615	Putative transport protein	gi|166240434	DDB0235163	132.4
5208	Isocitrate dehydrogenase (NAD^+^)	gi|60462281	DDB0231294	−52.7
5307	*S*-adenosyl-methionine synthetase	gi|60463691	DDB0230070	68.0
5415	3-phosphoglycerate dehydrogenase	gi|66813238	DDB0230052	43.3
6016	Actin binding protein	gi|60468343	DDB0215335	−30.0
6210	G β-like protein	gi|66820452	DDB0185122	−56.7
6607	Cyclase-associated protein	gi|66805581	DDB0191139	−50.8
6608	ATP citrate synthase	gi|66816585	DDB0235360	−44.7
6612	Aconitase, mitochondrial	gi|60470010	DDB0230168	−37.5

**Table 6:** Common proteins with altered expression levels after the various treatments. The variations in protein levels are expressed as percentages.

gi	Protein ID	Chlorpyrifos		Nickel		Mixture
	
		EC25	EC50	EC25	EC50	
gi|66805581	cyclase-associated protein	−37.4	/	−49.5	/	−50.8
gi|60469746	glutamate-ammonia ligase	−52.2	/	−35.6	/	/
gi|66818493	aldehyde dehydrogenase	−28.1	−24.5	/	/	90.3
gi|60463691	*S*-adenosyl-methionine synthetase	/	/	−35.4	/	68.0
gi|66803080	*S*-adenosylmethionine synthetase	/	−20.6	83.8	/	/
gi|22711882	phosphoglycerate kinase	/	/	/	−40.4	44.8
gi|60462281	isocitrate dehydrogenase (NAD+)	−34.5	/	/	/	−52.7
