# Supplementary material for: Effects of Nickel, Chlorpyrifos and Their Mixture on the Dictyostelium discoideum Proteome
Source: Int J Mol Sci. 2012 Nov 23;13(12):15679–705. doi: 10.3390/ijms131215679 (PMC3546656; doi:10.3390/ijms131215679)
Supplement: Supplementary file 1 [file ijms-13-15679-s001.pdf]

Supplemental Figure 1. Detection of intracellular Reactive Oxigen Species (ROS) production.

A statical significance of p value ≤ 0.05 is indicated as * (Mann-Whitney test).

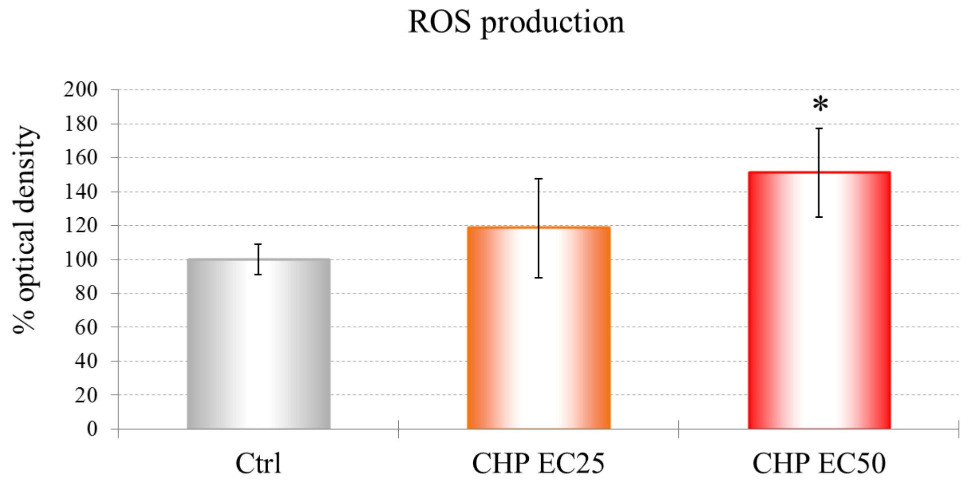

Methods

The detection of Intracellular ROS Production was evaluated with the method previously described by Marsano et al. (2010). Briefly, 40 μL of amoebae culture was pipetted onto microscope slides, in a wet chamber at room temperature. The slides were washed with PAS solution to eliminate culture medium that is highly fluorescent. The cells were treated for 30 min at room temperature with a solution of PAS containing 3 μM of dihydrorhodamine (DHR 123). DHR 123 reacted with intracellular ROS to the fluorescent rhodamine 123.

The excess of reagent was washed with PAS solution. Differences in fluorescence between treated and control samples were evaluated by an inverted fluorescence microscope equipped with a FITC filter. The images obtained were analyzed using an image analysis system (Scion Image freeware v 1.62).

Data analysis was conducted used Winks SDA (ver. 6.0.5) statistics software (Texasoft, Duncanville, TX). A non parametric test Mann-Whitney was used to compare control and treated samples. A p value ≤0.05 was consider statistically significant.

Reference

Marsano F., Boatti L., Ranzato E., Cavaletto M., Magnelli V., Dondero F., Viarengo A. *Effects of mercury on Dictyostelium discoideum: proteomics reveals the molecular mechanisms of physiological adaptation and toxicity*. J Proteome Res. 2010 Jun 4;9(6):2839-54 – DOI: 10.1021/pr900914t
