# Supplementary material for: Effects of Nickel, Chlorpyrifos and Their Mixture on the Dictyostelium discoideum Proteome
Source: Int J Mol Sci. 2012 Nov 23;13(12):15679–705. doi: 10.3390/ijms131215679 (PMC3546656; doi:10.3390/ijms131215679)

Supplemental Figure 2. Typical 2-DE gel of the proteins extracted from *Dictyostelium discoideum* amoebae. This pattern was highly reproducible, similar results being obtained in 7 different biological replicates for each treatment (see also Supplemental Table 2).

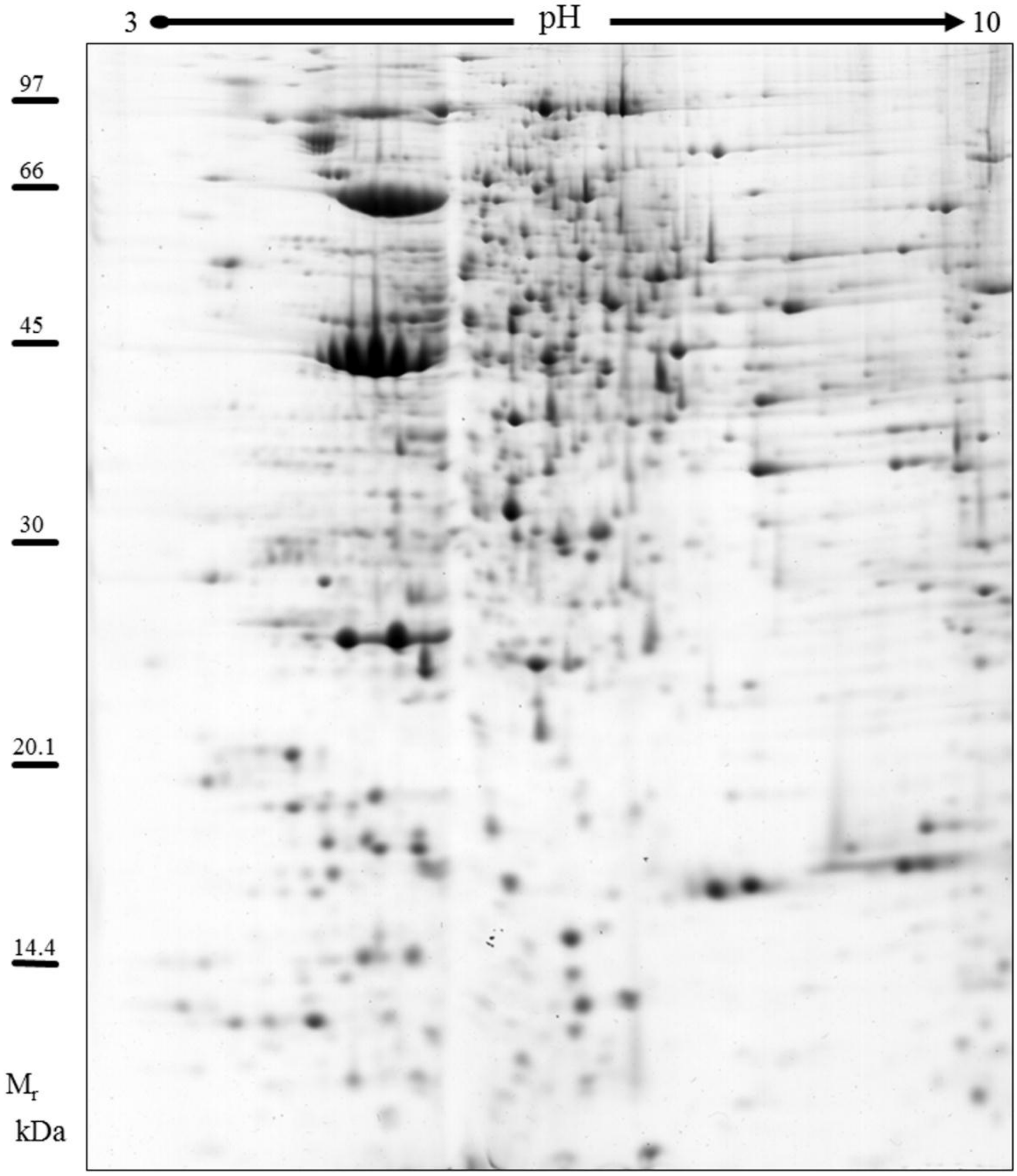

Supplement: Supplementary file 2 [file ijms-13-15679-s002.pdf]
