# Supplementary material for: Effects of Nickel, Chlorpyrifos and Their Mixture on the Dictyostelium discoideum Proteome
Source: Int J Mol Sci. 2012 Nov 23;13(12):15679–705. doi: 10.3390/ijms131215679 (PMC3546656; doi:10.3390/ijms131215679)

Supplemental information - Blast2GO analysis

Methods

The fasta sequences of the proteins were downloaded from http://www.ncbi.nlm.nih.gov using the GI code.

Data analysis was performed with Blast2GO using standard parameters. To obtain the pie charts of biological process and molecular function distribution - under the different experimental conditions - the proteins were classified with GO terms and two different cut-off levels – 2, 3 were applied.

The data obtained with cut-off level = 3 are very poorly informative. We reported only the pie charts obtained with cut-off level 2.

All the proteins comigrating in a single spot were considered for the Blast2GO analysis.

Results

Nickel EC25 – proteins up regulated – 2 proteins

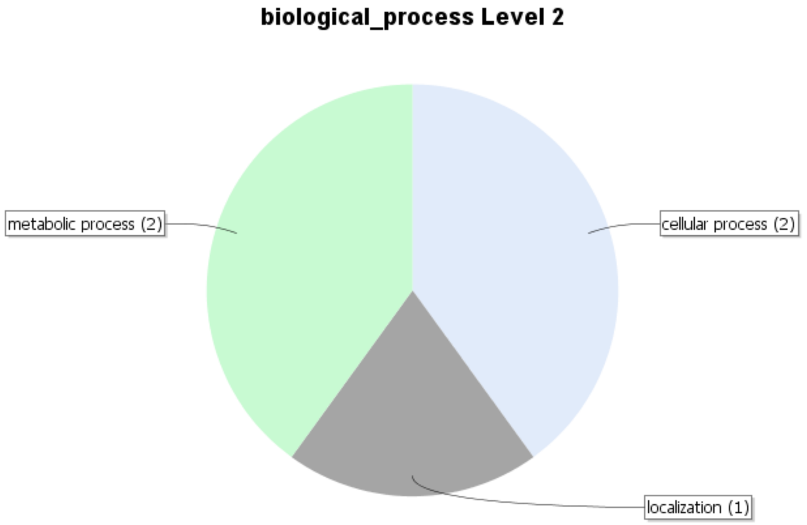

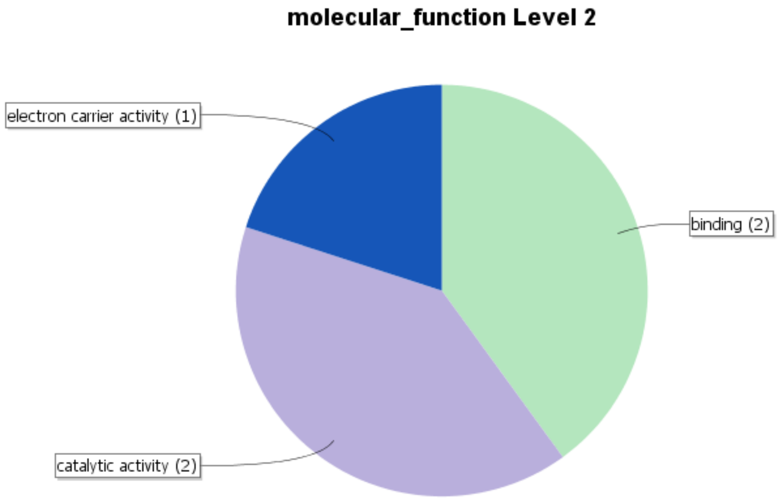

Nickel EC25 – proteins down regulated – 14 proteins

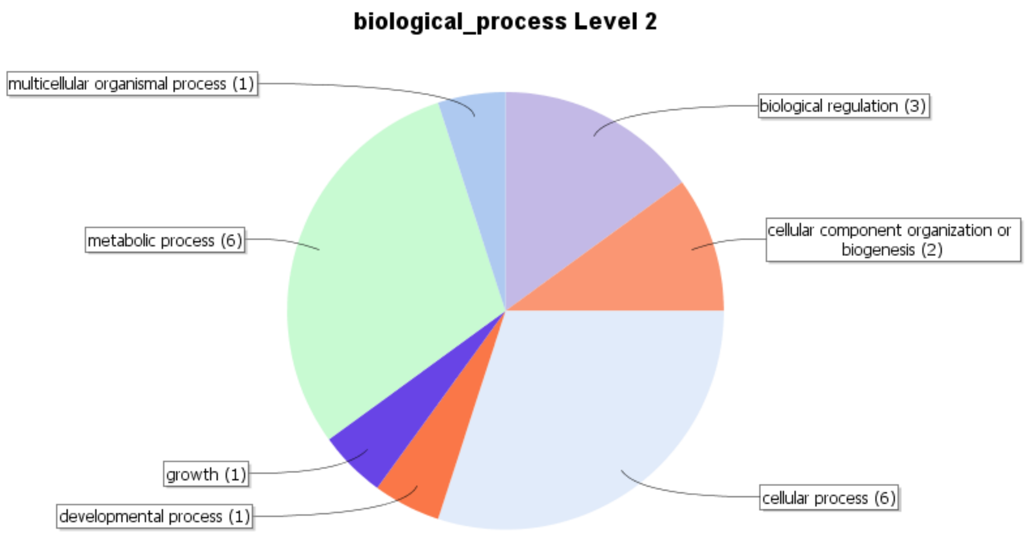

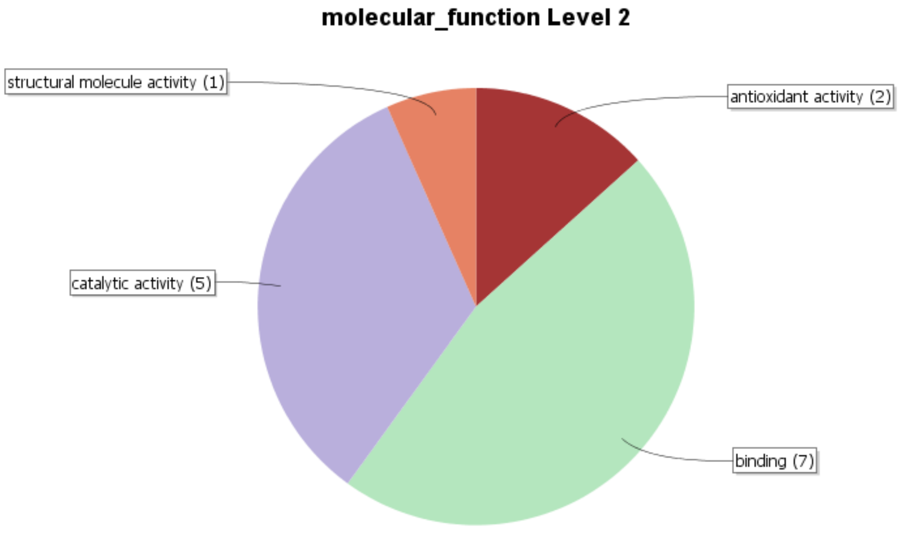

Nickel EC50 – proteins up regulated – 6 proteins

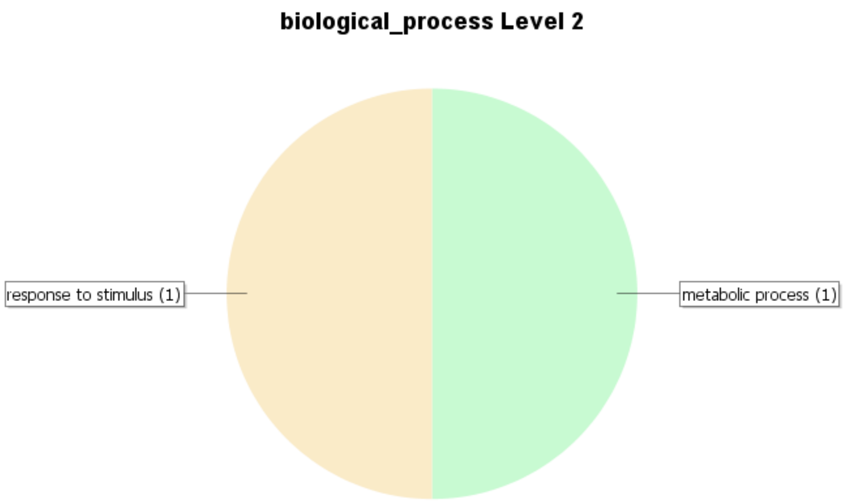

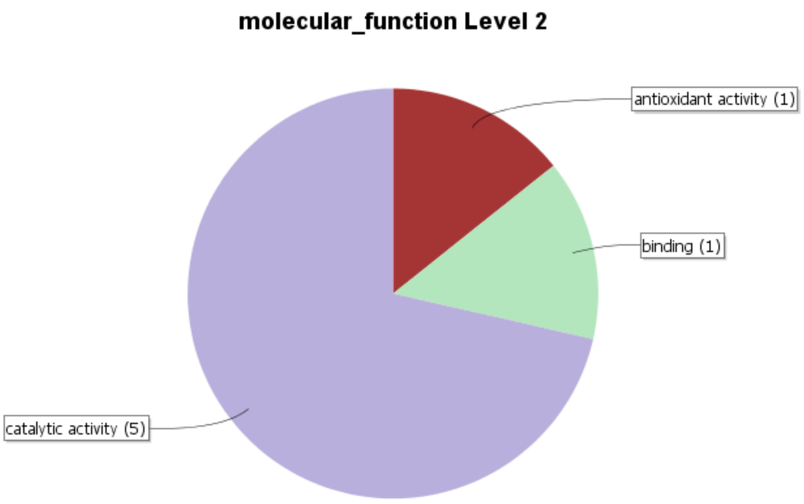

Nickel EC50 – proteins down regulated – 12 proteins

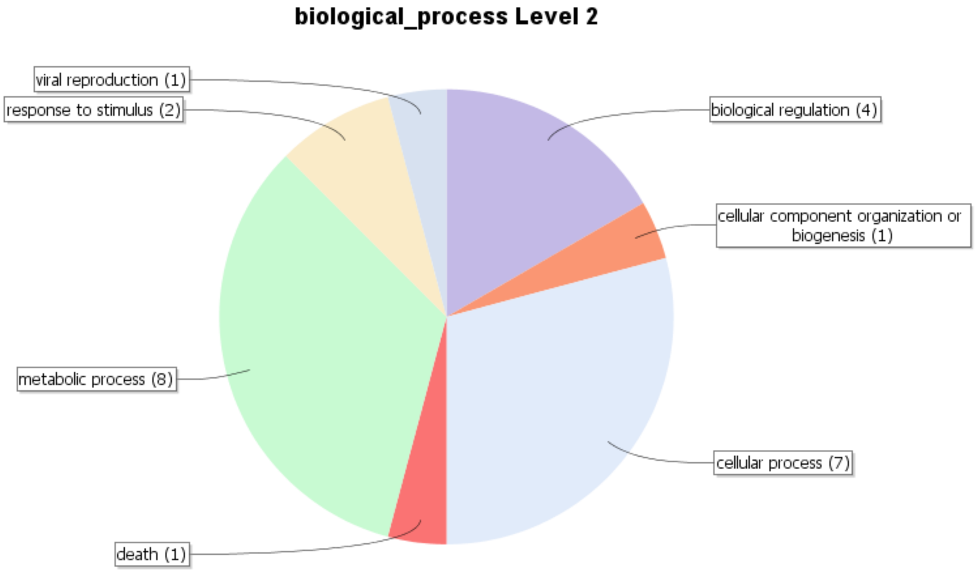

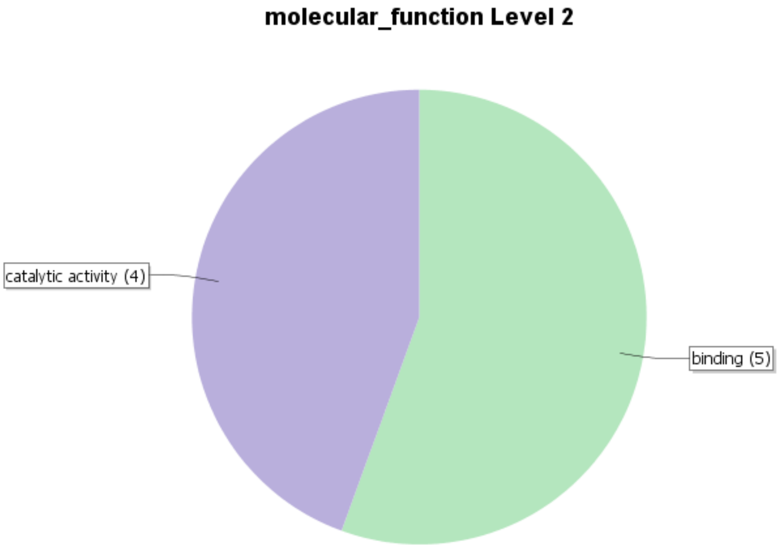

Chlorpyrifos EC25 – proteins down regulated – 6 proteins

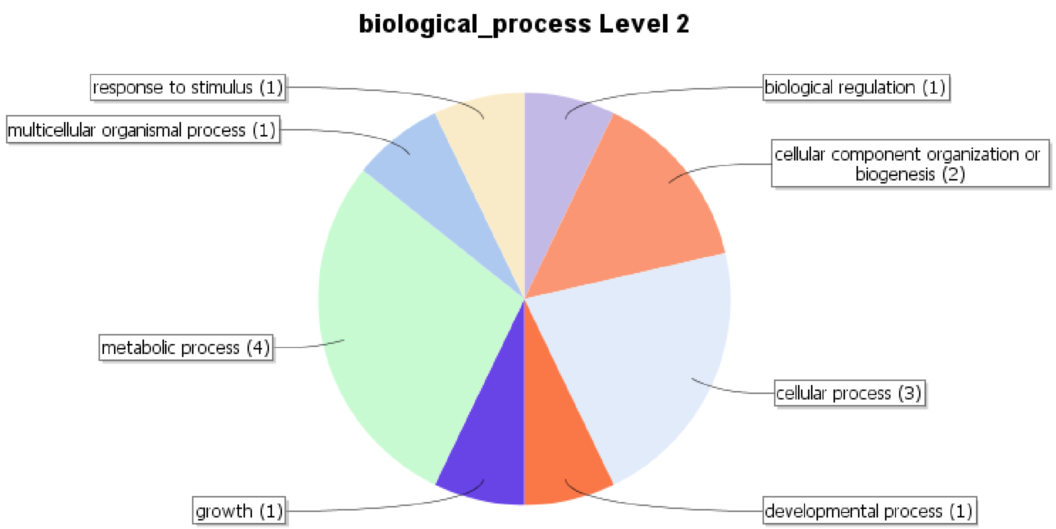

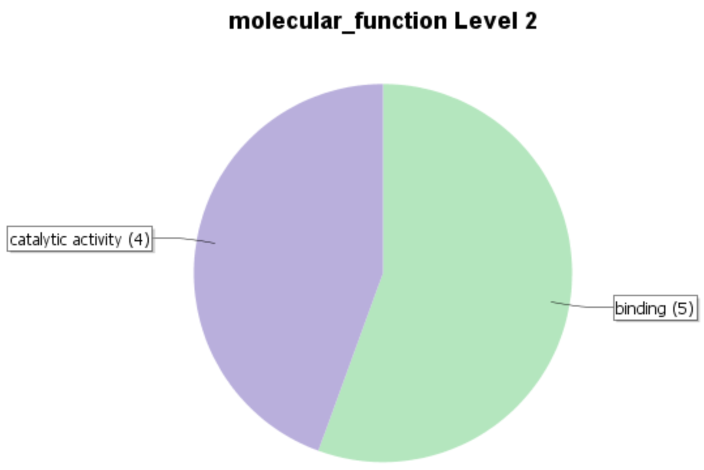

Chlorpyrifos EC50 – proteins up regulated – 8 proteins

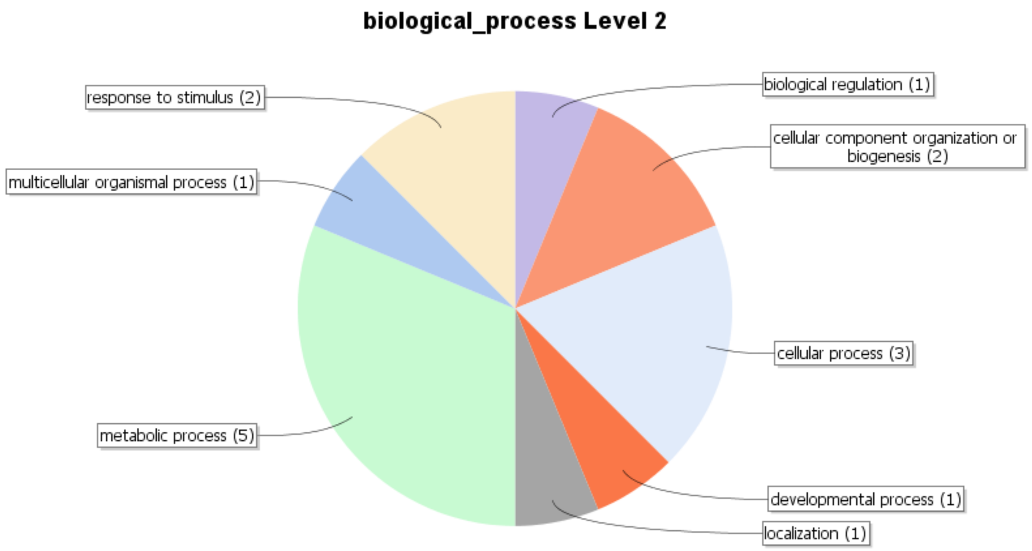

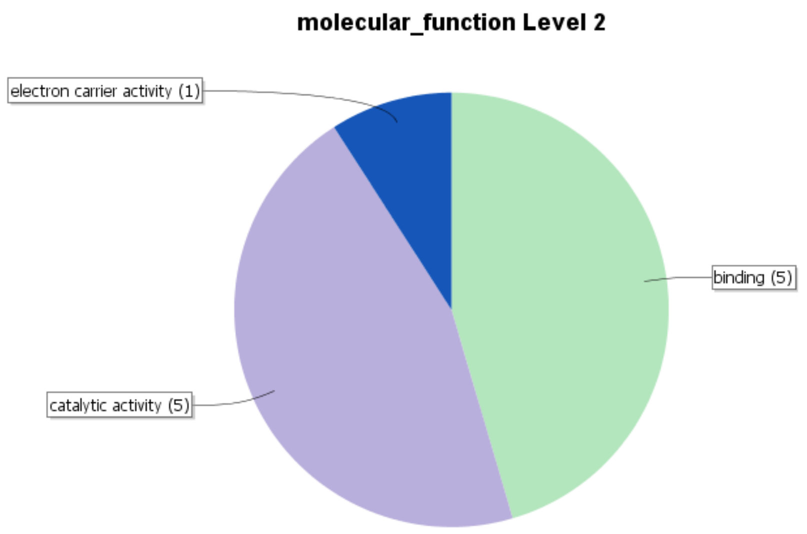

Chlorpyrifos EC50 – proteins down regulated – 5 proteins

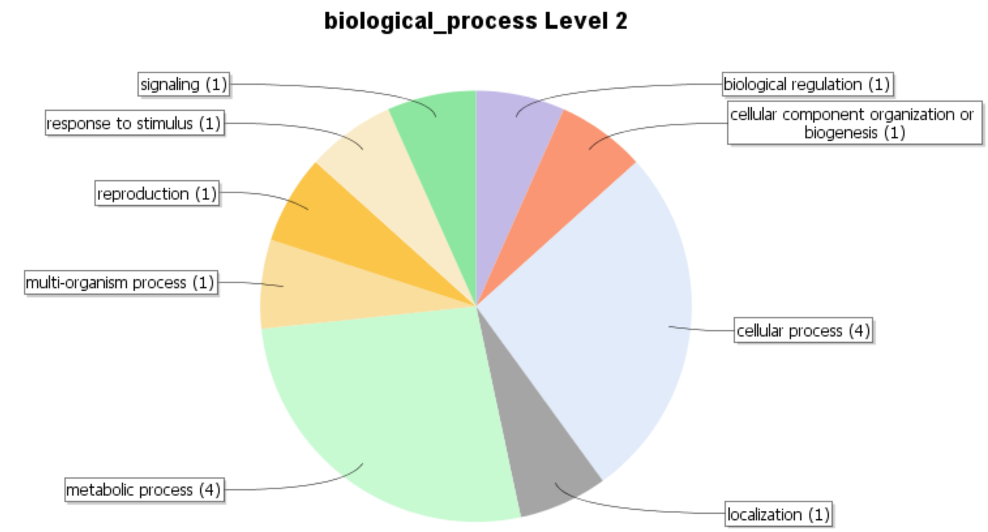

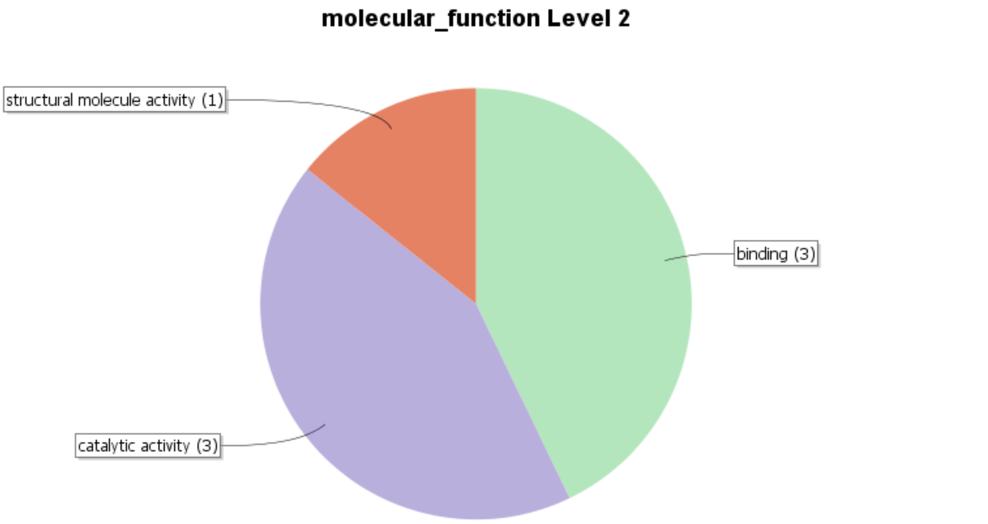

Mixture - proteins up regulated – 6 proteins

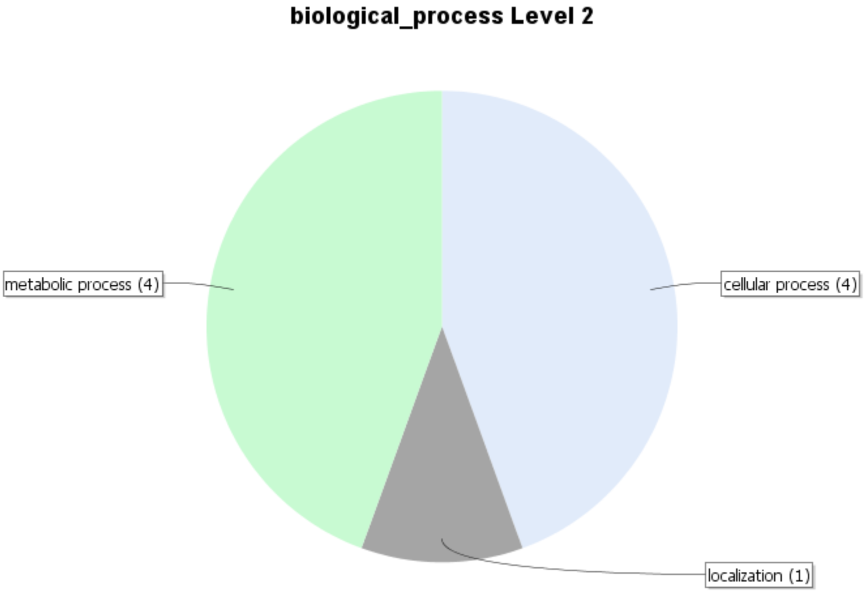

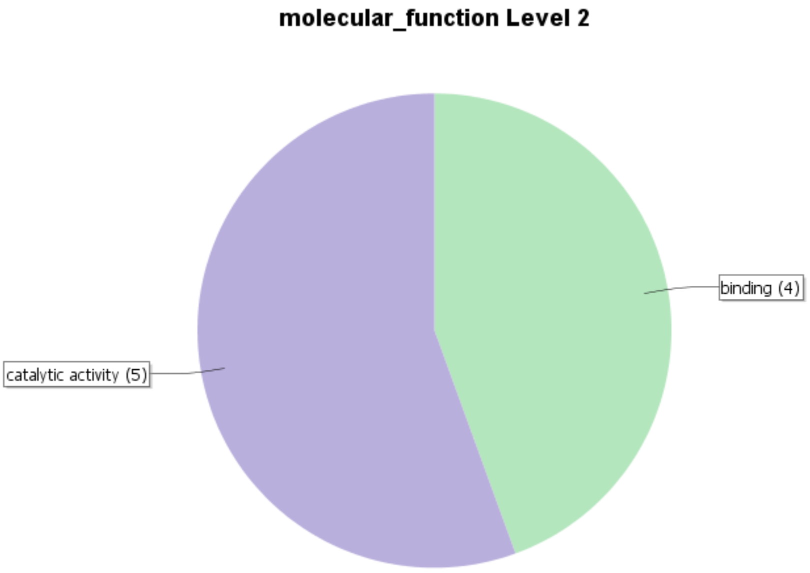

Mixture - proteins down regulated – 12 proteins

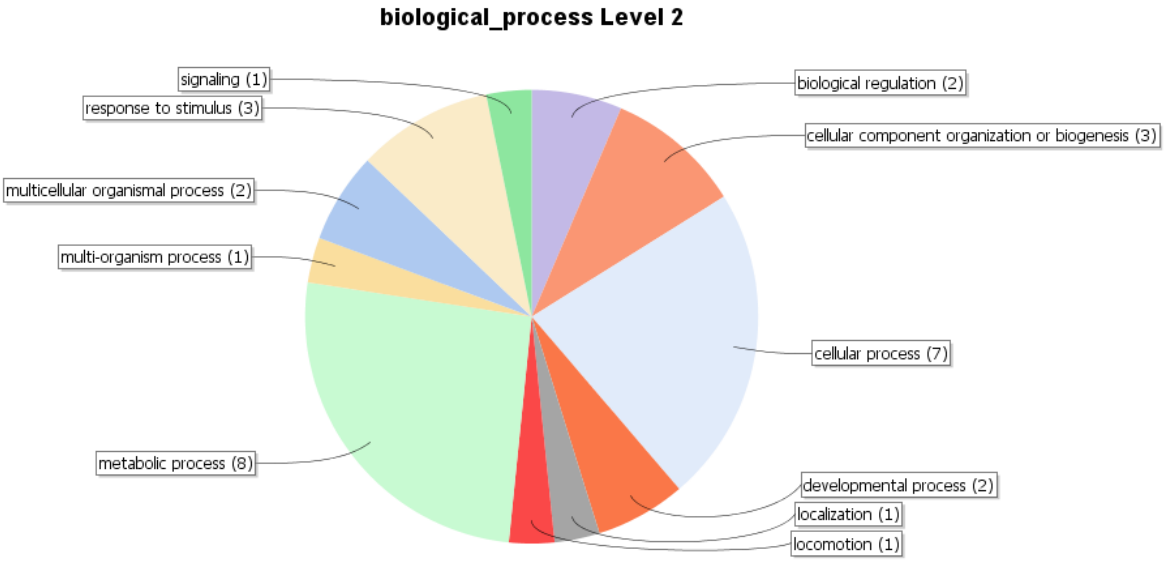

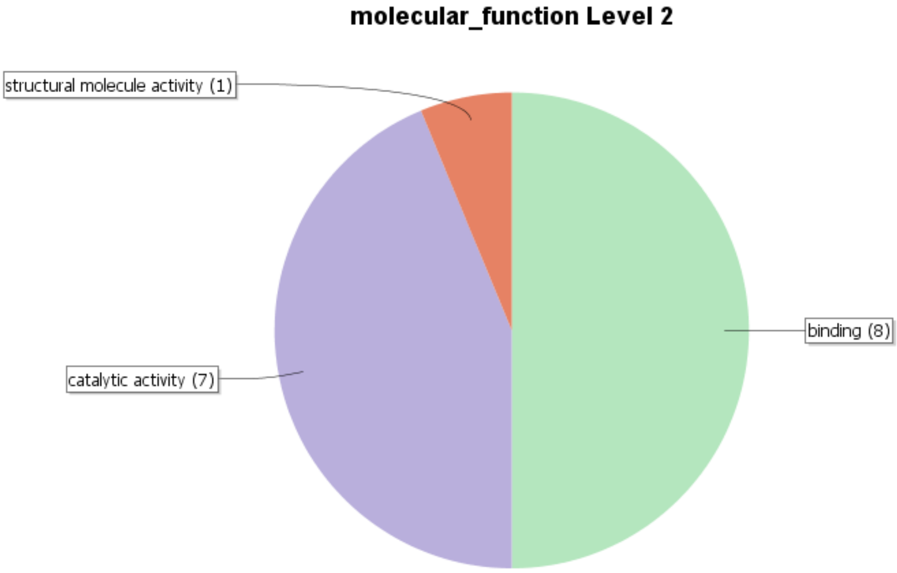

Supplement: Supplementary file 3 [file ijms-13-15679-s003.pdf]
