# Supplementary material for: Effects of Nickel, Chlorpyrifos and Their Mixture on the Dictyostelium discoideum Proteome
Source: Int J Mol Sci. 2012 Nov 23;13(12):15679–705. doi: 10.3390/ijms131215679 (PMC3546656; doi:10.3390/ijms131215679)
Supplement: Supplementary file 4 [file ijms-13-15679-s004.pdf]

Supplemental table 1 - Details about identified proteins

Treatment	Spot n°	ID by www.ncbi.nlm.nih.gov		ID by www.dictybase.org			UniPROT ID	MW(Da)/pI expected	MW(Da)/pI observed	n° matches	n° sequences	% coverage	mass					peptide				
		gi|	accession number	DDB	protein ID	gene name							observed	Mr(expt)	charge	Mr(calc)	delta	miss	score	ion score (identity)	sequence	modifications
Ni EC25	0013	133059	P22683	DDB0191484	60S acidic ribosomal protein P2	rplP2	P22683	10559/4.90	10270/4.91	3 (3)	3 (3)	58%	1049.5668	2097.1140	2	2097.1098	0.0042	0	54	47	K.YLAAYLLASLSGNANAASVTK.I	
													687.0397	2058.0954	3	2058.0796	0.0158	1	56	45	K.ILQSVGVEVDAARVESVCK.E	
													726.0988	2175.2582	3	2175.1579	0.1003	0	51	45	K.VGSVAAAAAPAAATSAAPAAAAAK.K	
Ni EC25	0106	7107412	AAF36404	DDB0201567	LIM-type zinc finger-containing protein	limD	Q54EY5	22386/4.70	26720/4.98	4 (4)	4 (4)	27%	602.2880	1202.5615	2	1202.6118	-0.0503	0	56	45	K.VQLFPTNCPK.C	Carbamidomethyl (C)
													603.7979	1205.5812	2	1205.6404	-0.0592	0	64	45	K.ILQLGQYSER.D	
													501.9145	1502.7217	3	1502.7517	-0.0301	1	53	47	K.AYFNELKVYNSR.D	
													704.9841	2111.9306	3	2112.0310	-0.1004	1	82	47	K.NLVSGQYSEKEGLIYCPR.C	Carbamidomethyl (C)
Ni EC25	1108	66803106	XP_635396	DDB0216235	rho GDP-dissociation inhibitor	rdiA	Q95UQ1	22383/6.00	21500/5.63	6 (3)	5 (3)	39%	600.3016	1797.8831	3	1797.9989	-0.1158	0	57	55	K.ITLTFNIQHDIVSGLK.Q	
													620.3040	1857.8902	3	1857.9836	-0.0934	0	44	55	K.IQIEGRPDTIYPLDTK.E	
													678.6590	2032.9551	3	2033.0265	-0.0714	0	39	53	K.HMLGSFAPQALAHSVTNPR.H	
													683.9389	2048.7950	3	2049.0214	-0.2265	0	55	55	K.HMLGSFAPQALAHSVTNPR.H	Oxidation (M)
													819.6792	2456.0159	3	2456.1384	-0.1225	0	57	53	K.VVFTDDDNEEHLSVEYAFSIK.S	
													649.2804	2593.0925	4	2593.3071	-0.2146	1	36	54	K.VSTEKHMLGSFAPQALAHSVTNPR.H	Oxidation (M)
Ni EC25	1115	66806101	XP_636772	DDB0191138	actin related protein 2/3 complex, subunit 5	arcE	O96626	15128/5.05	18180/5.27	6 (4)	5 (3)	42%	690.8199	1379.6252	2	1379.7085	-0.0833	0	59	57	R.GLATGENSPIFFK.W	
													816.8947	1631.7749	2	1631.8883	-0.1133	0	45	56	K.DQNATIVLNLLGSFK.D	
													626.0003	1874.9792	3	1875.0102	-0.0310	1	32	56	K.DQNATIVLNLLGSFKDK.D	
													701.7081	2102.1024	3	2102.1735	-0.0712	1	59	57	K.TGAIKDQNATIVLNLLGSFK.D	
													702.7105	2105.1096	3	2105.1256	-0.0159	1	55	53	K.TGAIKDQNATIVLNLLGSFK.D	3 Deamidated (NQ)
													794.0671	2379.1795	3	2379.2798	-0.1003	0	56	53	K.ALNAGKPQPQDALNVALADPPIYTK.T	
Ni EC25	3711	60469746	EAL67734	DDB0231551	glutamate-ammonia ligase	glnA3	Q54WR9	83077/5.80	76430/6.23	8 (5)	8 (5)	13%	462.7477	923.4809	2	923.5804	-0.0994	0	23	43	R.TLIGAKPPK.G	
													520.7356	1039.4567	2	1039.5702	-0.1135	0	48	43	K.YSEILFLR.-	
													561.7514	1121.4882	2	1121.6193	-0.1311	0	58	45	R.AVDIHADLLR.A	
													648.7744	1295.5343	2	1295.6761	-0.1418	0	47	44	R.QEILFEIYNK.S	
													688.7599	1375.5053	2	1375.6367	-0.1314	0	47	43	K.GEPDASSFPSGGIR.S	
													550.2231	1647.6474	3	1647.8045	-0.1571	1	44	43	R.TSPFAFTGNKFEFR.A	
													863.9113	1725.8080	2	1725.9664	-0.1585	0	24	44	R.LGANEAPPAIISIYLGK.E	
													669.3195	2004.9365	3	2005.0884	-0.1518	1	23	43	R.ISPTLGIEQEFFLIDRK.F	

Supplemental table 1 - Details about identified proteins

Treatment	Spot n°	ID by www.ncbi.nlm.nih.gov		ID by www.dictybase.org			UniPROT ID	MW(Da)/pI expected	MW(Da)/pI observed	n° matches	n° sequences	% coverage	mass					peptide				
		gi|	accession number	DDB	protein ID	gene name							observed	Mr(expt)	charge	Mr(calc)	delta	miss	score	ion score (identity)	sequence	modifications
Ni EC25	4106	28828367	AAO51015	DDB0231647	peroxiredoxin	prdx4	Q555L5	23234/6.09	25460/6.56	7 (4)	5 (3)	31%	433.9084	1298.7033	3	1298.7558	-0.0525	1	57	53	R.GSILIDKEGLVR.V	
													650.3643	1298.7141	2	1298.7558	-0.0416	1	62	57	R.GSILIDKEGLVR.V	
													556.2546	1665.7419	3	1665.7635	-0.0216	0	64	55	K.DYGVYIEEDGHTIR.G	
													597.2857	1788.8353	3	1788.9410	-0.1057	1	21	53	R.KPAPAFKGQAVVNGEFK.E	2 Deamidated (NQ)
													721.3781	2161.1126	3	2161.1743	-0.0617	0	56	53	K.EGGLGGINIPLLSDLTHQISK.D	
													764.0539	2289.1398	3	2289.2692	-0.1294	1	21	54	R.KEGGLGGINIPLLSDLTHQISK.D	
													573.2971	2289.1592	4	2289.2692	-0.1101	1	34	55	R.KEGGLGGINIPLLSDLTHQISK.D	
Ni EC25	5108	66823097	XP_644903	DDB0237752	dihydropteridine reductase	qdpr	Q86A17	24625/6.43	25230/6.79	9 (5)	8 (4)	55%	798.9336	1595.8526	2	1595.9247	-0.0720	0	76	57	K.NILVLGGSGALGAEVVK.F	
													957.9704	1913.9263	2	1914.0687	-0.1424	0	60	57	K.LLNQGGLFVLTGASAALNR.T	
													639.3400	1914.9982	3	1915.0527	-0.0545	0	66	57	K.LLNQGGLFVLTGASAALNR.T	Deamidated (NQ)
													673.7113	2018.1121	3	2018.1564	-0.0443	1	22	55	K.NILVLGGSGALGAEVVKFFK.S	
													676.9654	2027.8744	3	2027.9445	-0.0700	0	60	55	K.GMIDMNLYSAFASAHIGAK.L	Oxidation (M)
													713.6565	2137.9477	3	2138.0280	-0.0803	0	14	53	K.LFEWSTNSDSRPTNGSLVK.F	Deamidated (NQ)
													781.6601	2341.9586	3	2342.1399	-0.1813	1	15	53	K.SVKGMIDMNLYSAFASAHIGAK.L	2 Oxidation (M)
													787.9897	2360.9474	3	2361.0471	-0.0997	1	28	55	R.KYMSDANFDDWTPLSEVAEK.L	Oxidation (M)
													956.0974	2865.2704	3	2865.4720	-0.2016	0	56	55	K.DLASENGGLPAGSTSLGILPVTLDTPTNR.K	
		66815899	XP_641966	DDB0206195	regulator of microtubule dynamics protein 1	DDB_G0278793	Q54XR4	25954/7.06	25230/6.79	4 (2)	4 (2)	19%	636.3246	1270.6346	2	1270.7132	-0.0787	0	57	55	K.LIEQALELTNK.A	
													692.8123	1383.6101	2	1383.7034	-0.0934	0	18	55	R.NALFTGDTYAALK.D	
													604.9563	1811.8471	3	1811.9053	-0.0583	1	48	53	R.NALFTGDTYAALKDNAK.A	
													621.6396	1861.8971	3	1862.0010	-0.1039	0	69	56	K.ANELKPNDATTLHLLGR.W	
		66821043	XP_644052	DDB0231647	peroxiredoxin	prdx4	Q555L5	22949/6.09	25230/6.79	3 (2)	3 (2)	22%	650.3380	1298.6615	2	1298.7558	-0.0943	1	55	53	R.GSILIDKEGLVR.V	
													556.2302	1665.6688	3	1665.7635	-0.0946	0	61	57	K.DYGVYIEEDGHTIR.G	
													721.3706	2161.0899	3	2161.1743	-0.0844	0	28	55	K.EGGLGGINIPLLSDLTHQISK.D	

Supplemental table 1 - Details about identified proteins

Treatment	Spot n°	ID by www.ncbi.nlm.nih.gov		ID by www.dictybase.org			UniPROT ID	MW(Da)/pI expected	MW(Da)/pI observed	n° matches	n° sequences	% coverage	mass					peptide				
		gi|	accession number	DDB	protein ID	gene name							observed	Mr(expt)	charge	Mr(calc)	delta	miss	score	ion score (identity)	sequence	modifications
Ni EC25	5412	66803080	XP_635383	DDB0230070	S-adenosylmethionine synthetase	metK	Q54F07	41737/6.14	45050/6.59	12 (6)	9 (4)	26%	486.7301	971.4456	2	971.5400	-0.0944	0	42	53	K.SLVAAGLADR.C	
													514.7674	1027.5203	2	1027.6277	-0.1074	1	47	52	R.ADLKELVIK.A	
													676.2447	1350.4749	2	1350.6092	-0.1343	0	49	51	R.NDPDFTWETVK.E	
													**679.8276**	**1357.6406**	**2**	**1357.7718**	**-0.1312**	**0**	**63**	**52**	**R.DLQLTRPIFQK.T**	
													**697.3024**	**1392.5902**	**2**	**1392.7357**	**-0.1455**	**0**	**67**	**53**	**K.TGMVMILGEITTK.A**	
													705.2993	1408.5841	2	1408.7306	-0.1465	0	49	51	K.TGMVMILGEITTK.A	Oxidation (M)
													**713.3043**	**1424.5941**	**2**	**1424.7255**	**-0.1314**	**0**	**62**	**51**	**K.TGMVMILGEITTK.A**	**2 Oxidation (M)**
													**732.2961**	**1462.5776**	**2**	**1462.7239**	**-0.1463**	**0**	**74**	**53**	**R.FVIGGPMGDSGLTGR.K**	
													**740.2970**	**1478.5794**	**2**	**1478.7188**	**-0.1394**	**0**	**68**	**53**	**R.FVIGGPMGDSGLTGR.K**	**Oxidation (M)**
													747.7598	1493.5050	2	1493.6674	-0.1624	1	30	53	K.IGYDDSSKGFDYK.T	
													804.3302	1606.6459	2	1606.8138	-0.1679	1	37	51	R.FVIGGPMGDSGLTGRK.I	Oxidation (M)
													**650.6102**	**1948.8089**	**3**	**1948.9432**	**-0.1343**	**0**	**55**	**52**	**K.IIIDSYGGWGAHGGGAFSGK.D**	
Ni EC25	5718	66813780	XP_641069	DDB0214886	succinate dehydrogenase	sdhA	Q9U3X4	68472/6.46	62360/6.63	10 (5)	8 (5)	21%	636.8148	1271.6150	2	1271.6721	-0.0571	0	19	55	R.AVANEIENTLAK.D	
													699.3181	1396.6216	2	1396.6908	-0.0692	0	17	55	R.ETAMIFAGVDVTK.E	Oxidation (M)
													**737.3869**	**1472.7593**	**2**	**1472.8351**	**-0.0758**	**0**	**67**	**57**	**R.LGANSLLDIVVFGR.A**	
													**498.5848**	**1492.7324**	**3**	**1492.7998**	**-0.0673**	**0**	**67**	**54**	**R.GVVAINLEDGTIHR.F**	
													747.5000	1492.9854	2	1492.7998	0.1857	0	17	53	R.GVVAINLEDGTIHR.F	
													**569.2766**	**1704.8079**	**3**	**1704.8795**	**-0.0716**	**1**	**59**	**53**	**R.YAPSVADLASRDVVSR.S**	
													**1017.4664**	**2032.9182**	**2**	**2033.0470**	**-0.1288**	**0**	**58**	**57**	**K.EAVPTVLELEQYGVPFSR.M**	
													**740.3390**	**2217.9952**	**3**	**2218.1019**	**-0.1066**	**0**	**75**	**57**	**R.DYAVVDHTYDAIVVGAGGAGLR.A**	
													819.0317	2454.0732	3	2454.2503	-0.1770	0	17	53	K.DTPHKPLPPNAGEESIANIDAIR.F	
													614.7966	2455.1574	4	2455.2343	-0.0769	0	16	55	K.DTPHKPLPPNAGEESIANIDAIR.F	Deamidated (NQ)
Ni EC25	6106	66821505	XP_644221	DDB0167610	similar to ribosomal protein	DDB_G0274407	Q86KA1	19511/9.13	20950/7.32	2 (2)	2 (2)	29%	**688.0028**	**2060.9866**	**3**	**2061.0590**	**-0.0724**	**1**	**54**	**53**	**K.LTTIENGKSSTEVADLVER.I**	
													**996.1797**	**2985.5172**	**3**	**2985.6134**	**-0.0963**	**1**	**57**	**52**	**K.KLSIPDIDLSSLGSGAPAGGAAPAAAAAPAAVAK.T**	
Ni EC25	6414	60463691	EAL61873	DDB0230070	S-adenosyl-methionine synthetase	metK	Q54F07	42079/6.14	42420/7.18	6 (5)	4 (3)	15%	514.8598	1027.7050	2	1027.6277	0.0773	1	27	41	R.ADLKELVIK.A	
													**697.4315**	**1392.8484**	**2**	**1392.7357**	**0.1128**	**0**	**51**	**47**	**K.TGMVMILGEITTK.A**	
													**713.4197**	**1424.8248**	**2**	**1424.7255**	**0.0993**	**0**	**62**	**47**	**K.TGMVMILGEITTK.A**	**2 Oxidation (M)**
													**732.4202**	**1462.8259**	**2**	**1462.7239**	**0.1021**	**0**	**57**	**47**	**R.FVIGGPMGDSGLTGR.K**	
													**740.4217**	**1478.8288**	**2**	**1478.7188**	**0.1100**	**0**	**67**	**45**	**R.FVIGGPMGDSGLTGR.K**	**Oxidation (M)**
													**781.7931**	**2342.3574**	**3**	**2342.1655**	**0.1919**	**0**	**65**	**47**	**K.AVVPAQYLDDNTIYHLNPSGR.F**	

Supplemental table 1 - Details about identified proteins

Treatment	Spot n°	ID by www.ncbi.nlm.nih.gov		ID by www.dictybase.org			UniPROT ID	MW(Da)/pI expected	MW(Da)/pI observed	n° matches	n° sequences	% coverage	mass					peptide				
		gi|	accession number	DDB	protein ID	gene name							observed	Mr(expt)	charge	Mr(calc)	delta	miss	score	ion score (identity)	sequence	modifications
Ni EC25	7112	60475381	EAL73316	DDB0233902	hypothetical protein DDB_0233902	DDB_ G0267726	Q55GC5	19517/6.90	21000/8.20	5 (4)	4 (3)	41%	687.3722	1372.7252	2	1372.6987	0.0265	0	38	41	R.VILVNESNGWNK.A	Deamidated (NQ)
													**699.3801**	**1396.7546**	**2**	**1396.7027**	**0.0519**	**0**	**65**	**47**	**K.YSIQTIDFPWK.S**	
													**739.3951**	**1476.7711**	**2**	**1476.7612**	**0.0099**	**0**	**59**	**47**	**K.ALGIWYDLEEIR.I**	
													**858.9899**	**1715.9746**	**2**	**1715.9359**	**0.0387**	**0**	**54**	**45**	**K.SGGQQYIALQLWKPK.A**	
													**573.0015**	**1715.9751**	**3**	**1715.9359**	**0.0392**	**0**	**63**	**45**	**K.SGGQQYIALQLWKPK.A**	
Ni EC25	7414	10801150	AAG23402	DDB0191174	elongation factor 1 beta	efa1B	Q9GRF8	24220/4.54	39090/8.30	4 (3)	4 (3)	29%	**722.3772**	**1442.7358**	**2**	**1442.7102**	**0.0256**	**1**	**56**	**45**	**K.TAPCATKYPHAAR.W**	
													**693.0136**	**2076.0405**	**3**	**2075.9643**	**0.0762**	**0**	**54**	**45**	**R.WFNTIASYSAAEQGQFEK.V**	
													684.9389	1367.8632	2	1367.7721	0.0911	0	37	43	K.VTETVTIAAPAAPK.A	
													**696.8784**	**1391.7558**	**2**	**1391.6789**	**0.0769**	**0**	**62**	**47**	**R.SIEMDGLVWGASK.L**	
Ni EC25	7609	66805581	XP_636512	DDB0191139	cyclase associated protein	cap	P54654	49611/6.97	54250/7.83	7 (5)	7 (5)	22%	389.2746	776.5347	2	776.4796	0.0551	1	17	32	R.ILKEFK.G	
													**562.8352**	**1123.6559**	**2**	**1123.5873**	**0.0686**	**1**	**49**	**31**	**K.NFTDKSSVVK.A**	
													**564.8236**	**1127.6326**	**2**	**1127.5822**	**0.0503**	**0**	**46**	**31**	**K.EIVIAEPDSR.Q**	
													**762.4610**	**1522.9074**	**2**	**1522.8355**	**0.0719**	**0**	**71**	**30**	**K.LAPEVGNQVEQLVK.A**	
													962.0340	1922.0534	2	1921.9972	0.0562	0	16	31	R.QTVYIFQCVNSLVQIK.G	Carbamidomethyl (C); Gln->pyro-Glu (N-term Q)
													**816.8008**	**2447.3807**	**3**	**2447.2544**	**0.1263**	**0**	**38**	**31**	**K.SATPAPASSAPAAPVAPAVSST PVESK.K**	
													**847.1907**	**2538.5501**	**3**	**2538.4057**	**0.1445**	**0**	**40**	**32**	**K.KPSQETLLELIKPLNNFAAEV GK.I**	
Ni EC50	0124	66812950	XP_640654	DDB0233328	putative polypeptide-associated complex alpha subunit	nacA	Q54U07	17357/5.08	21390/4.81	3 (2)	3 (2)	13%	**487.2490**	**972.4835**	**2**	**972.5855**	**-0.1020**	**0**	**51**	**43**	**K.VVETLIATK.N**	
													**594.2901**	**1186.5657**	**2**	**1186.6710**	**-0.1052**	**0**	**44**	**41**	**K.LGLAPVSDIFR.V**	
													615.8158	1229.6170	2	1229.7231	-0.1060	1	33	41	R.EKVVETLIATK.N	
Ni EC50	0125	66826197	XP_646453	DDB0191107	cytosolic glycoprotein FP21	fpaA	P52285	18706/4.74	21370/4.55	8 (5)	7 (5)	41%	**381.7043**	**761.3941**	**2**	**761.4799**	**-0.0859**	**1**	**47**	**43**	**R.KIFNIK.N**	
													**382.6678**	**763.3211**	**2**	**763.4116**	**-0.0904**	**0**	**51**	**43**	**K.VFEIEK.E**	
													**584.2381**	**1166.4616**	**2**	**1166.5675**	**-0.1059**	**0**	**44**	**41**	**K.EIACMSVTIK.N**	**Carbamidomethyl (C); Oxidation (M)**
													630.2619	1258.5092	2	1258.6306	-0.1214	1	36	41	K.RLDDIPPYDR.D	
													**638.7750**	**1275.5355**	**2**	**1275.6558**	**-0.1203**	**1**	**52**	**43**	**M.SLVKLESSDEK.V**	**N-Acetyl (Protein)**
													**776.8170**	**1551.6195**	**2**	**1551.7668**	**-0.1473**	**1**	**43**	**41**	**K.LESSDEKVFEIEK.E**	
													904.7178	2711.1316	3	2711.3575	-0.2259	0	28	41	K.NMIEDIGESDSPIPLPNVTSTILE K.V	
													910.0512	2727.1318	3	2727.3524	-0.2206	0	27	41	K.NMIEDIGESDSPIPLPNVTSTILE K.V	Oxidation (M)

Supplemental table 1 - Details about identified proteins

Treatment	Spot n°	ID by www.ncbi.nlm.nih.gov		ID by www.dictybase.org			UniPROT ID	MW(Da)/pI expected	MW(Da)/pI observed	n° matches	n° sequences	% coverage	mass					peptide				
		gi|	accession number	DDB	protein ID	gene name							observed	Mr(expt)	charge	Mr(calc)	delta	miss	score	ion score (identity)	sequence	modifications
Ni EC50	0526	4336714	AAD17913	DDB0191177	repC-binding protein A	rcbA	Q54LV1	37621/4.89	56020/4.37	6 (3)	5 (3)	19%	383.1675	1146.4807	3	1146.6046	-0.1239	0	31	41	R.NHPHFNLLR.E	
													574.2492	1146.4839	2	1146.6046	-0.1207	0	17	40	R.NHPHFNLLR.E	
													**694.2874**	**1386.5602**	**2**	**1386.6891**	**-0.1289**	**0**	**41**	**40**	**R.QIQENPNEFIR.L**	
													**748.3710**	**2242.0913**	**3**	**2242.2797**	**-0.1884**	**0**	**48**	**41**	**K.NPSIIPGILQQLAQTNPALVR.Q**	
													803.3544	2407.0413	3	2407.2634	-0.2221	1	13	40	K.NINKEIYVFEVNGDLTVAELK.N	
													**924.4549**	**2770.3429**	**3**	**2770.5704**	**-0.2275**	**1**	**49**	**43**	**R.EAISKNPSIIPGILQQLAQTNPALVR.Q**	
		66810606	XP_639010	DDB0191384	calreticulin	crtA	Q23858	48506/4.63	56020/4.37	3 (2)	3 (2)	8%	493.2375	984.4604	2	984.5756	-0.1152	0	36	41	R.VHVILNYK.G	
													**539.7627**	**1077.5108**	**2**	**1077.6070**	**-0.0962**	**0**	**49**	**41**	**K.DLVLQYTVK.N**	
													**650.7846**	**1299.5546**	**2**	**1299.6823**	**-0.1277**	**0**	**47**	**43**	**K.QSKPVDWVDVK.E**	
Ni EC50	0721	66814268	XP_641313	DDB0215015	glucose-regulated protein 94	grp94	Q9NKX1	87227/4.88	87220/4.88	3 (2)	3 (2)	5%	**699.7779**	**1397.5413**	**2**	**1397.6827**	**-0.1414**	**0**	**52**	**42**	**K.EGQDQIYFITGK.S**	
													772.8446	1543.6746	2	1543.8205	-0.1459	1	37	43	R.ELISNASDALDKIR.F	
													**929.9177**	**1857.8209**	**2**	**1857.9836**	**-0.1628**	**0**	**55**	**43**	**R.FLVGVIDSDDLPLNVSR.E**	
Ni EC50	1125	66812950	XP_640654	DDB0233328	putative nascent polypeptide-associated complex alpha subunit	nacA	Q54U07	17357/5.08	21100/5.08	2 (2)	2 (2)	6%	**594.2902**	**1186.5658**	**2**	**1186.6710**	**-0.1052**	**0**	**66**	**45**	**K.LGLAPVSDIFR.V**	
													**615.8176**	**1229.6287**	**2**	**1229.7231**	**-0.0944**	**1**	**59**	**43**	**R.EKVVETLIATK.N**	
Ni EC50	2334	66825457	XP_646083	DDB0201767	N-acyl-L-amino-acid amidohydrolase	acy1	Q55DP8	46824/5.69	42460/6.00	9 (4)	8 (4)	24%	413.1854	824.3563	2	824.4432	-0.0869	0	19	43	K.FASYIPK.N	
													**497.6821**	**993.3497**	**2**	**993.4515**	**-0.1018**	**0**	**51**	**43**	**K.DESGNIFGR.G**	
													506.7285	1011.4424	2	1011.5600	-0.1176	0	38	41	K.IEGLEPNLK.T	
													**545.2379**	**1088.4612**	**2**	**1088.5654**	**-0.1043**	**1**	**44**	**41**	**K.FVYTEKFR.Q**	
													**750.8797**	**1499.7448**	**2**	**1499.8922**	**-0.1474**	**1**	**53**	**41**	**K.KLGDVTSLNLTVLK.A**	
													515.5408	1543.6004	3	1543.7670	-0.1666	1	25	43	K.AKEYNIPYEVYR.E	
													**772.8215**	**1543.6284**	**2**	**1543.7670**	**-0.1386**	**1**	**46**	**41**	**K.AKEYNIPYEVYR.E**	
													582.8955	1745.6647	3	1745.8220	-0.1573	1	29	43	K.IRTDHPTPDYESSTK.F	
													982.7356	2945.1849	3	2945.4307	-0.2458	0	25	41	K.AGIPIDHSNNFSYNVIPTQAEAGFDIR.I	
		66826503	XP_646606	DDB0232985	26S proteasome non-ATPase regulatory subunit 6	psmD6	Q55C75	44309/5.71	42460/6.00	6 (4)	6 (4)	15%	**421.7247**	**841.4349**	**2**	**841.5273**	**-0.0924**	**0**	**42**	**41**	**K.ILVEQLK.W**	
													545.2664	1088.5182	2	1088.6593	-0.1412	0	39	43	K.LDIVFTLIR.M	
													**557.7712**	**1113.5278**	**2**	**1113.6070**	**-0.0792**	**1**	**52**	**41**	**R.LKTYEAVYK.M**	
													581.7379	1161.4612	2	1161.5666	-0.1053	0	27	41	K.WTEDQGLVSK.L	
													**659.3046**	**1316.5946**	**2**	**1316.7299**	**-0.1353**	**1**	**52**	**43**	**K.IDKVSGVIETTR.S**	
													**798.3067**	**1594.5989**	**2**	**1594.7474**	**-0.1486**	**0**	**47**	**41**	**K.ITDSVENFGESEIR.E**	

Supplemental table 1 - Details about identified proteins

Treatment	Spot n°	ID by www.ncbi.nlm.nih.gov		ID by www.dictybase.org			UniPROT ID	MW(Da)/pI expected	MW(Da)/pI observed	n° matches	n° sequences	% coverage	mass					peptide				
		gi|	accession number	DDB	protein ID	gene name							observed	Mr(expt)	charge	Mr(calc)	delta	miss	score	ion score (identity)	sequence	modifications
Ni EC50	2831	66799895	XP_628873	DDB0192196	hypothetical protein DDBDRAFT_0192196	DDB_ G0293920	Q54B43	97775/5.44	96880/6.02	14 (4)	14 (4)	18%	360.6945	719.3745	2	719.4581	-0.0836	0	18	41	R.LFVLTK.Y	
													492.2393	982.4641	2	982.5712	-0.1071	0	34	41	K.LLINAFHR.I	
													**501.2699**	**1000.5252**	**2**	**1000.6280**	**-0.1028**	**1**	**46**	**41**	**R.KLVLAGSVSK.G**	
													518.2471	1034.4797	2	1034.5760	-0.0963	0	24	40	K.GALSFAQTIK.S	
													560.7906	1119.5666	2	1119.6036	-0.0370	0	31	42	K.AEPIVDHIAR.T	
													**686.7952**	**1371.5759**	**2**	**1371.7146**	**-0.1387**	**0**	**53**	**42**	**R.ESHLYNLVEIR.T**	
													**691.2827**	**1380.5508**	**2**	**1380.6673**	**-0.1165**	**0**	**63**	**42**	**R.VFNLDDFAGIDR.N**	
													**747.3339**	**1492.6532**	**2**	**1492.7885**	**-0.1354**	**0**	**43**	**41**	**K.TTVAISSISTPNFR.A**	
													748.8642	1495.7139	2	1495.8497	-0.1359	0	23	41	R.TLIDIPVPTADITK.I	
													503.8880	1508.6422	3	1508.7947	-0.1525	1	22	41	K.SSSKAEPIVDHIAR.T	
													812.8763	1623.7381	2	1623.8943	-0.1562	0	29	40	R.NSEGAVNLTPVLAALR.H	
													**558.5874**	**1672.7403**	**3**	**1672.9049**	**-0.1646**	**0**	**46**	**41**	**K.EIPLLLADVFHHNR.T**	
													582.2316	1743.6730	3	1743.8652	-0.1922	0	29	40	K.SSSNIAHLNLANNSFR.K	
													660.3101	1977.9086	3	1978.0735	-0.1649	0	19	41	R.KPLVHINLEETQLDTTK.V	
Ni EC50	3423	22711882	AAG34561	DDB0191349	phosphoglycerate kinase	pgkA	Q9GPM4	45966/6.01	49510/6.79	12 (8)	10 (8)	36%	**669.9107**	**1337.8154**	**2**	**1337.8030**	**0.0123**	**1**	**51**	**47**	**K.VLEDQLKRPIK.F**	
													**691.3743**	**1380.7266**	**2**	**1380.7183**	**0.0080**	**0**	**47**	**43**	**R.AHSSMVGINLPQK.A**	
													699.3605	1396.7521	2	1396.7132	0.0380	0	28	41	R.AHSSMVGINLPQK.A	Oxidation (M)
													**739.3381**	**1476.7626**	**2**	**1476.7435**	**0.0190**	**0**	**46**	**43**	**K.TIVWNGPMGVFEK.S**	
													747.3883	1492.7574	2	1492.7384	0.0190	0	25	41	K.TIVWNGPMGVFEK.S	Oxidation (M)
													**761.8976**	**1521.7874**	**2**	**1521.7748**	**0.0126**	**0**	**56**	**47**	**R.IDASIPTLEYCLK.N**	
													**802.3815**	**1602.7561**	**2**	**1602.7161**	**0.0399**	**0**	**63**	**47**	**R.FHIEEEGSGVDAEGK.K**	
													**813.9051**	**1625.8421**	**2**	**1625.8300**	**0.0121**	**1**	**54**	**45**	**K.FIDNKEIGSSLFEK.T**	
													817.9105	1633.8016	2	1633.7848	0.0168	0	37	41	K.LGDVYVNDAFGTAHR.A	
													**849.9802**	**1697.9444**	**2**	**1697.9715**	**-0.0271**	**0**	**49**	**45**	**K.ALESPSKPFLAILGGAK.V**	
													866.4100	1730.8194	2	1730.8110	0.0084	1	41	45	R.FHIEEEGSGVDAEGKK.V	
													**778.7167**	**2333.1371**	**3**	**2333.1691**	**-0.0320**	**1**	**62**	**47**	**K.LHFPVDYVIADKFDNDANIK.T**	

Supplemental table 1 - Details about identified proteins

Treatment	Spot n°	ID by www.ncbi.nlm.nih.gov		ID by www.dictybase.org			UniPROT ID	MW(Da)/pI expected	MW(Da)/pI observed	n° matches	n° sequences	% coverage	mass					peptide				
		gi|	accession number	DDB	protein ID	gene name							observed	Mr(expt)	charge	Mr(calc)	delta	miss	score	ion score (identity)	sequence	modifications
Ni EC50	3721	66815105	XP_641648	DDB0231551	glutamate-ammonia ligase	glnA3	Q54WR9	83077/5.80	82320/6.67	8 (5)	8 (5)	13%	**462.7489**	**923.4801**	**2**	**923.5804**	**-0.1003**	**0**	**52**	**41**	**R.TLIGAKPPK.G**	
													520.7542	1039.4748	2	1039.5702	-0.0954	0	33	43	K.YSEILFLR.-	
													**561.7635**	**1121.5270**	**2**	**1121.6193**	**-0.0923**	**0**	**53**	**44**	**R.AVDIHADLLR.A**	
													**648.7934**	**1295.5840**	**2**	**1295.6761**	**-0.0921**	**0**	**51**	**44**	**R.QEILFEIYNK.S**	
													**688.7782**	**1375.5456**	**2**	**1375.6367**	**-0.0911**	**0**	**53**	**41**	**K.GEPDASSFPSGGIR.S**	
													**550.2343**	**1647.7167**	**3**	**1647.8045**	**-0.0878**	**1**	**47**	**42**	**R.TSPFAFTGNKFEFR.A**	
													863.9265	1725.8511	2	1725.9664	-0.1153	0	34	41	R.LGANEAPPAIISIYLGK.E	
													669.3301	2004.9872	3	2005.0884	-0.1012	1	33	41	R.ISPTLGIEQEFFLIDRK.F	
		4584573	CAB40787	DDB0185041	prolyl oligopeptidase	dpoA	Q86AS5	87987/5.76	82320/6.67	8 (3)	8 (3)	12%	516.2167	1030.4188	2	1030.5447	-0.1259	0	23	41	K.YSPLNNVPK.D	
													517.7108	1033.4071	2	1033.5192	-0.1121	0	28	41	R.NFQEAIANK.S	
													**527.7774**	**1053.5402**	**2**	**1053.6546**	**-0.1144**	**1**	**51**	**43**	**K.KVDTPLLIR.V**	
													**602.7397**	**1203.4648**	**2**	**1203.5771**	**-0.1123**	**0**	**57**	**45**	**K.AGSDWEEIAVK.K**	
													**621.2465**	**1240.4784**	**2**	**1240.6088**	**-0.1303**	**1**	**42**	**41**	**R.RDDSVFDIFK.S**	
													592.9246	1775.7521	3	1775.9305	-0.1784	1	31	45	K.EIIPEKDYVLESVSR.S	
													922.3581	1842.7017	2	1842.8788	-0.1770	0	23	45	K.GFENAIEFLNPNTYSK.D	
													643.6102	1927.8088	3	1928.0043	-0.1955	1	36	41	K.NDELLLFKEPHIEGFK.S	
Ni EC50	3826	66825911	XP_646310	DDB0191259	major vault protein	mvpA	P34118	94072/6.05	94290/6.89	12 (6)	11 (6)	15%	**569.7939**	**1137.5732**	**2**	**1137.7121**	**-0.1390**	**0**	**66**	**45**	**K.VTALQVVAPLK.A**	
													**601.7918**	**1201.5690**	**2**	**1201.6918**	**-0.1228**	**0**	**42**	**41**	**K.SVQLAIEITTK.S**	
													651.3224	1300.6302	2	1300.7350	-0.1048	1	17	41	K.AIVESIGRDTLK.S	
													**711.7592**	**1421.5038**	**2**	**1421.6787**	**-0.1749**	**0**	**49**	**41**	**R.QSVFGLDESGEVR.K**	
													739.9020	1477.7895	2	1477.9344	-0.1450	1	22	40	K.VTALQVVAPLKALR.L	
													756.2849	1510.5553	2	1510.7126	-0.1574	0	32	40	K.IFQVPDFVGDSCK.A	Carbamidomethyl (C)
													756.7744	1511.5342	2	1511.6967	-0.1624	0	31	41	K.IFQVPDFVGDSCK.A	Carbamidomethyl (C); Deamidated (NQ)
													**769.7984**	**1537.5823**	**2**	**1537.7453**	**-0.1630**	**0**	**44**	**41**	**R.FSQEPFPLYPGEK.I**	
													**530.9199**	**1589.7378**	**3**	**1589.9028**	**-0.1651**	**1**	**46**	**41**	**K.VVEIVNAYVLTDKK.A**	
													800.3346	1598.6547	2	1598.8160	-0.1613	0	21	40	K.LVSGPEPMIMIPQR.N	2 Oxidation (M)
													**817.3163**	**1632.6180**	**2**	**1632.7995**	**-0.1814**	**0**	**62**	**45**	**R.ESIPLDENEGIYVR.D**	
													833.3078	1664.6010	2	1664.7893	-0.1883	0	31	41	R.NENGSLVLDEYGQVK.L	Deamidated (NQ)

Supplemental table 1 - Details about identified proteins

Treatment	Spot n°	ID by www.ncbi.nlm.nih.gov		ID by www.dictybase.org			UniPROT ID	MW(Da)/pI expected	MW(Da)/pI observed	n° matches	n° sequences	% coverage	mass					peptide				
		gi|	accession number	DDB	protein ID	gene name							observed	Mr(expt)	charge	Mr(calc)	delta	miss	score	ion score (identity)	sequence	modifications
Ni EC50	4529	66827977	XP_647343	DDB0191115	actin binding protein	corA	P27133	49697/6.81	56150/7.21	9 (4)	9 (4)	25%	425.7074	849.4003	2	849.4960	-0.0956	0	30	45	K.VITVGFSK.T	
													495.7342	989.4539	2	989.5480	-0.0941	1	33	45	R.GLCFLPKR.C	
													**547.2630**	**1092.5115**	**2**	**1092.6179**	**-0.1063**	**1**	**53**	**47**	**K.DKVITVGFSK.T**	
													**639.8123**	**1277.6101**	**2**	**1277.7230**	**-0.1130**	**1**	**52**	**47**	**R.VAYLESEIVKK.D**	
													**696.8153**	**1391.6160**	**2**	**1391.7449**	**-0.1289**	**0**	**51**	**45**	**K.VTPFTVEPISFR.V**	
													**727.7614**	**1453.5083**	**2**	**1453.6586**	**-0.1502**	**0**	**59**	**48**	**K.SAWDSNYVAANTR.Y**	
													604.9451	1811.8134	3	1811.9781	-0.1647	0	33	43	K.ASAVEFKPVVQVQEGPK.N	
													760.3012	2277.8818	3	2278.0833	-0.2015	0	14	43	R.YYELVDESPYIHFLSEFK.S	
													855.7163	2564.1271	3	2564.3485	-0.2215	1	23	45	R.KVGTISFNPVADNVAVTSSGDF LVK.T	
Ni EC50	4732	66805423	XP_636444	DDB0231600	AAA ATPase domain-containing protein	DDB_ G0289047	Q54I27	88859/8.51	84480/7.24	2 (2)	2 (2)	3%	**685.8560**	**1369.6974**	**2**	**1369.8181**	**-0.1207**	**0**	**60**	**43**	**R.VLTLDLASIVAGAK.F**	
													**772.3473**	**1542.6801**	**2**	**1542.8366**	**-0.1565**	**0**	**54**	**45**	**R.ITDSALVAAVVNSQR.Y**	
Ni EC50	6132	66808689	XP_638067	DDB0238212	peroxiredoxin	prdx5	Q54N76	18350/6.74	21630/8.22	2 (2)	2 (2)	14%	**606.2692**	**1210.5238**	**2**	**1210.6268**	**-0.1029**	**0**	**54**	**45**	**R.YAMILDSGVVK.H**	**Oxidation (M)**
													**721.2967**	**1440.5788**	**2**	**1440.7105**	**-0.1317**	**0**	**69**	**45**	**K.ALPPVDGVCAMAPK.V**	**Carbamidomethyl (C); Oxidation (M)**
Ni EC50	6218	66820416	XP_643826	DDB0167407	putative epimerase	DDB_ G0275053	Q86I47	33977/6.97	28190/8.02	8 (4)	8 (4)	33%	**553.8499**	**1105.6852**	**2**	**1105.6019**	**0.0833**	**0**	**48**	**41**	**K.LTNLEEFLK.K**	
													412.2803	1233.8190	3	1233.6969	0.1221	1	36	41	K.LTNLEEFLKK.T	
													535.0136	1602.0190	3	1601.8314	0.1876	1	31	41	K.ISGGRYNVPHFDLK.H	
													**614.0527**	**1839.1364**	**3**	**1838.9890**	**0.1474**	**0**	**61**	**45**	**K.ELEYGLNIAHAALAAGVK.H**	
													**740.7721**	**2219.2944**	**3**	**2219.1144**	**0.1800**	**0**	**64**	**45**	**R.TVNEATQEVTYVLGMPLNPK. M**	**Oxidation (M)**
													566.0851	2260.3112	4	2260.1232	0.1880	1	25	40	K.IIPFSGDCLHGEKIAEIMSK.V	Carbamidomethyl (C); Oxidation (M)
													**792.8106**	**2375.4099**	**3**	**2375.2155**	**0.1944**	**1**	**45**	**41**	**K.RTVNEATQEVTYVLGMPLNP K.M**	**Oxidation (M)**
													1217.1378	2432.2611	2	2432.1035	0.1576	0	12	4	K.ENPSFVTSFVYAPFYMQNFK.T	Deamidated (NQ); Oxidation (M)
Ni EC50	6223	66809487	XP_638466	DDB0237882	phosphoribosyl pyrophosphate synthetase	prsA	Q54PA9	34443/7.68	30880/8.29	3 (3)	3 (3)	13%	**692.8592**	**1383.7039**	**2**	**1383.7722**	**-0.0682**	**0**	**62**	**45**	**K.LVANLIETAGIDR.V**	
													**724.3359**	**1446.6573**	**2**	**1446.7388**	**-0.0815**	**0**	**54**	**45**	**K.ANEVSGMILVGDVK.D**	**Oxidation (M)**
													**836.8576**	**1671.7007**	**2**	**1671.7774**	**-0.0767**	**0**	**46**	**41**	**K.FSNGETSVMISESIR.D**	**Oxidation (M)**

Supplemental table 1 - Details about identified proteins

Treatment	Spot n°	ID by www.ncbi.nlm.nih.gov		ID by www.dictybase.org			UniPROT ID	MW(Da)/pI expected	MW(Da)/pI observed	n° matches	n° sequences	% coverage	mass					peptide				
		gi|	accession number	DDB	protein ID	gene name							observed	Mr(expt)	charge	Mr(calc)	delta	miss	score	ion score (identity)	sequence	modifications
Chp EC25	0605	66825227	XP_645968	DDB0304584	ZPR1-type zinc finger-containing protein	zpr1	Q55E13	53516/4.53	58980/4.51	27 (12)	17 (10)	51%	511.2930	1020.5714	2	1020.5491	0.0223	0	32	41	K.EELLGFVSK.L	
													**613.8344**	**1225.6542**	**2**	**1225.6417**	**0.0125**	**0**	**75**	**45**	**K.MVLTDIPYFK.S**	
													**621.8355**	**1241.6564**	**2**	**1241.6366**	**0.0198**	**0**	**59**	**45**	**K.MVLTDIPYFK.S**	**Oxidation (M)**
													436.8979	1307.6719	3	1307.6510	0.0209	1	17	41	K.EELEKNPFFR.G	
													654.8448	1307.6750	2	1307.6510	0.0240	1	37	41	K.EELEKNPFFR.G	
													**667.9154**	**1333.8162**	**2**	**1333.7857**	**0.0305**	**0**	**46**	**45**	**K.FTTVEGLITIIK.E**	
													703.3826	1404.7507	2	1404.7323	0.0184	0	25	40	K.LSNLLMVDEPFK.I	
													**711.3855**	**1420.7565**	**2**	**1420.7272**	**0.0293**	**0**	**54**	**45**	**K.LSNLLMVDEPFK.I**	**Oxidation (M)**
													**809.9264**	**1617.8382**	**2**	**1617.8210**	**0.0172**	**0**	**49**	**45**	**R.VTTTSVSIPSGDLADR.E**	
													850.4362	1698.8579	2	1698.8465	0.0114	0	22	45	R.YQEITSQLDSFIAGK.K	
													851.4261	1700.8376	2	1700.8145	0.0231	0	35	43	R.YQEITSQLDSFIAGK.K	2 Deamidated (NQ)
													**897.4076**	**1792.8006**	**2**	**1792.7834**	**0.0172**	**0**	**70**	**45**	**K.SILLMAFSCDECGYK.T**	**2 Carbamidomethyl (C)**
													**905.4115**	**1808.8085**	**2**	**1808.7783**	**0.0302**	**0**	**82**	**45**	**K.SILLMAFSCDECGYK.T**	**2 Carbamidomethyl (C); Oxidation (M)**
													619.9925	1856.9558	3	1856.9115	0.0443	0	24	41	K.EAITNLTNASEIHTESK.E	
													620.3193	1857.9362	3	1857.8956	0.0406	0	20	41	K.EAITNLTNASEIHTESK.E	Deamidated (NQ)
													**929.9768**	**1857.9390**	**2**	**1857.8956**	**0.0434**	**0**	**82**	**45**	**K.EAITNLTNASEIHTESK.E**	**Deamidated (NQ)**
													633.9649	1898.8729	3	1898.8266	0.0463	0	23	45	K.EIMVFAFHCPECGFR.S	2 Carbamidomethyl (C)
													639.2912	1914.8518	3	1914.8215	0.0303	0	40	41	K.EIMVFAFHCPECGFR.S	2 Carbamidomethyl (C); Oxidation (M)
													965.4988	1928.9831	2	1928.9439	0.0392	0	29	41	R.TAEQNASLGLGINEEQQK.Q	
													966.4865	1930.9584	2	1930.9119	0.0465	0	22	43	R.TAEQNASLGLGINEEQQK.Q	2 Deamidated (NQ)
													655.0463	1962.1170	3	1962.0925	0.0245	1	19	41	K.FTTVEGLITIIKEELEK.N	
													**1014.0097**	**2026.0048**	**2**	**2026.0007**	**0.0041**	**0**	**60**	**43**	**K.IIVDDPSGNSFIENPNAPK.A**	
													**793.7615**	**2378.2627**	**3**	**2378.2329**	**0.0298**	**0**	**76**	**43**	**K.SDTANVIIPELEIEVTHGSLGGK.F**	
													715.8854	2859.5124	4	2859.4501	0.0623	1	37	40	K.EAITNLTNASEIHTESKEELLGFVSK.L	
													954.1786	2859.5141	3	2859.4501	0.0640	1	23	40	K.EAITNLTNASEIHTESKEELLGFVSK.L	
													**1023.4550**	**3067.3433**	**3**	**3067.3135**	**0.0298**	**0**	**91**	**45**	**K.NISHENEVTEIESYCVNCEAENGITR.I**	**2 Carbamidomethyl (C); Deamidated (NQ)**
													**1112.2306**	**3333.6698**	**3**	**3333.6292**	**0.0406**	**0**	**77**	**45**	**K.KPFTIELDDPISNSYIQNLFAPDDDPQLK.I**	**Deamidated (NQ)**
Chp EC25	3711	60469746	EAL67734	DDB0231551	glutamate-ammonia ligase	glnA3	Q54WR9	83077/5.80	76430/6.23	6 (5)	6 (5)	9%	**561.8511**	**1121.6929**	**2**	**1121.6193**	**0.0736**	**0**	**56**	**43**	**R.AVDIHADLLR.A**	
													**648.8497**	**1295.6861**	**2**	**1295.6761**	**0.0100**	**0**	**65**	**47**	**R.QEILFEIYNK.S**	
													**688.8712**	**1375.7374**	**2**	**1375.6367**	**0.1007**	**0**	**59**	**47**	**K.GEPDASSFPSGGIR.S**	
													550.2943	1647.8517	3	1647.8045	0.0472	1	42	45	R.TSPFAFTGNKFEFR.A	
													**864.0201**	**1726.0411**	**2**	**1725.9664**	**0.0747**	**0**	**54**	**41**	**R.LGANEAPPAIISIYLGK.E**	

Supplemental table 1 - Details about identified proteins

Treatment	Spot n°	ID by www.ncbi.nlm.nih.gov		ID by www.dictybase.org			UniPROT ID	MW(Da)/pI expected	MW(Da)/pI observed	n° matches	n° sequences	% coverage	mass					peptide				
		gi|	accession number	DDB	protein ID	gene name							observed	Mr(expt)	charge	Mr(calc)	delta	miss	score	ion score (identity)	sequence	modifications
													**669.3694**	**2005.0985**	**3**	**2005.0884**	**0.0101**	**1**	**46**	**45**	**R.ISPTLGIEQEFFLIDRK.F**	
Chp EC25	4610	66818493	XP_642906	DDB0231504	aldehyde dehydrogenase	DDB_ G0276821	P83401	55155/5.78	53860/6.41	2 (2)	2 (2)	5%	**653.7964**	**1305.5783**	**2**	**1305.7041**	**-0.1258**	**0**	**57**	**45**	**R.AVLFASVGTTGQR.C**	
													**674.8157**	**1347.6169**	**2**	**1347.7609**	**-0.1440**	**0**	**78**	**49**	**K.GASTTSLITLAVSK.I**	
Chp EC25	4613	66826743	XP_646726	DDB0232964	26S proteasome ATPase 1 subunit	psmC1	Q55BV5	49161/5.90	59820/6.39	13 (8)	13 (8)	31%	**428.2099**	**854.4052**	**2**	**854.4208**	**-0.0155**	**0**	**52**	**41**	**R.IMSFVDK.S**	**Oxidation (M)**
													**479.7765**	**957.5384**	**2**	**957.5283**	**0.0100**	**0**	**55**	**43**	**K.IFEIHTAK.M**	
													**486.7975**	**971.5804**	**2**	**971.5651**	**0.0154**	**0**	**63**	**45**	**R.VVGSELIQK.Y**	
													536.3026	1070.5907	2	1070.6012	-0.0105	0	40	45	K.IEFPLPDIK.T	
													577.2927	1152.5708	2	1152.5485	0.0224	1	20	41	R.KTAGAPEGLYM.-	Oxidation (M)
													**595.8338**	**1189.6530**	**2**	**1189.6342**	**0.0188**	**0**	**55**	**41**	**K.GVILYGEPGTGK.T**	
													**600.3659**	**1198.7172**	**2**	**1198.6961**	**0.0211**	**1**	**45**	**40**	**R.KIEFPLPDIK.T**	
													**639.8528**	**1277.6911**	**2**	**1277.6728**	**0.0183**	**0**	**58**	**41**	**K.AVANQTSATFLR.V**	
													652.3639	1302.7133	2	1302.6965	0.0167	0	25	41	K.AICTESGLLALR.E	Carbamidomethyl (C)
													**659.8882**	**1317.7618**	**2**	**1317.7656**	**-0.0038**	**0**	**62**	**42**	**K.LYLGATVLLNNK.T**	
													678.8497	1355.6849	2	1355.6874	-0.0025	0	21	42	K.YQPPPPPTQFGK.K	
													484.2924	1449.8554	3	1449.8303	0.0250	0	16	40	R.IETLDPALIRPGR.I	
													**839.4763**	**2515.4072**	**3**	**2515.3573**	**0.0499**	**0**	**48**	**43**	**K.EAIELPLTHPELYEEIGIKPPK .G**	
Chp EC25	6310	66799989	XP_628920	DDB0231294	isocitrate dehydrogenase (NAD+)	idhB	Q54B68	38663/8.48	37240/7.61	16 (8)	14 (7)	44%	428.2565	854.4985	2	854.4974	0.0011	0	23	41	K.QIPGITAR.H	
													446.2133	890.4120	2	890.4174	-0.0054	0	26	41	R.YAFEYAK.A	
													**539.2787**	**1076.5428**	**2**	**1076.5349**	**0.0079**	**0**	**50**	**41**	**K.EGTLTSDIGGK.S**	
													**560.2995**	**1118.5844**	**2**	**1118.5832**	**0.0012**	**0**	**65**	**45**	**K.HLGLNEHATK.V**	
													683.8610	1365.7074	2	1365.6816	0.0258	0	28	45	K.QFTGAVIDYIEK.N	Gln->pyro-Glu (N-term Q)
													**692.3619**	**1382.7092**	**2**	**1382.7081**	**0.0011**	**0**	**48**	**42**	**K.QFTGAVIDYIEK.N**	
													693.3800	1384.7454	2	1384.7173	0.0282	0	20	41	K.ALDLYAHVVPCK.Q	Carbamidomethyl (C)
													**476.2625**	**1425.7658**	**3**	**1425.7616**	**0.0042**	**0**	**54**	**45**	**R.HDDVLVDFVVIR.E**	
													**713.9141**	**1425.8137**	**2**	**1425.7616**	**0.0522**	**0**	**59**	**45**	**R.HDDVLVDFVVIR.E**	
													744.9210	1487.8274	2	1487.8195	0.0080	1	22	41	K.AVIKEGTLTSDIGGK.S	
													**754.4057**	**1506.7969**	**2**	**1506.7678**	**0.0291**	**0**	**56**	**40**	**K.GPLYTEILSGSQSR.N**	
													**795.9400**	**1589.8654**	**2**	**1589.8520**	**0.0133**	**0**	**47**	**40**	**K.ANPTGLLLASVMMLK.H**	**2 Oxidation (M)**
													612.0151	1833.0236	3	1832.9739	0.0496	1	35	42	K.DKANPTGLLLASVMMLK.H	2 Oxidation (M)
													640.3706	1918.0900	3	1918.0523	0.0377	1	23	42	K.VALKGPLYTEILSGSQSR.N	
													826.1040	2475.2902	3	2475.2492	0.0410	0	17	43	R.ENTQGEYSGLEQVLTPGVVQSL K.I	
													**864.1405**	**2589.3996**	**3**	**2589.3359**	**0.0637**	**0**	**51**	**45**	**K.TVTVIPGDGIGPEITSSVMGV FQAAK.V**	**Oxidation (M)**

Supplemental table 1 - Details about identified proteins

Treatment	Spot n°	ID by www.ncbi.nlm.nih.gov		ID by www.dictybase.org			UniPROT ID	MW(Da)/pI expected	MW(Da)/pI observed	n° matches	n° sequences	% coverage	mass					peptide				
		gi|	accession number	DDB	protein ID	gene name							observed	Mr(expt)	charge	Mr(calc)	delta	miss	score	ion score (identity)	sequence	modifications
Chp EC25	7609	66805581	XP_636512	DDB0191139	cyclase associated protein	cap	P54654	49611/6.97	54250/7.83	15 (9)	14 (9)	41%	**562.8330**	**1123.6515**	**2**	**1123.5873**	**0.0642**	**1**	**48**	**45**	**K.NFTDKSSVVK.A**	
													**604.3354**	**1206.6562**	**2**	**1206.5979**	**0.0582**	**0**	**64**	**45**	**K.DSLETEIVSSK.S**	
													616.3750	1230.7354	2	1230.6608	0.0745	0	19	41	K.GPGLGAVFGELSK.G	
													680.4244	1358.8342	2	1358.7558	0.0784	1	25	41	K.KGPGLGAVFGELSK.G	
													**762.4584**	**1522.9022**	**2**	**1522.8355**	**0.0667**	**0**	**76**	**43**	**K.LAPEVGNQVEQLVK.A**	
													**551.3375**	**1650.9906**	**3**	**1650.9305**	**0.0601**	**1**	**47**	**45**	**K.KLAPEVGNQVEQLVK.A**	
													**830.4641**	**1658.9137**	**2**	**1658.8839**	**0.0298**	**1**	**63**	**45**	**K.AIDAEKALINTASQSK.K**	
													970.5619	1939.1093	2	1939.0237	0.0856	0	22	43	R.QTVYIFQCVNSLVQIK.G	Carbamidomethyl (C)
													660.7228	1979.1467	3	1979.0397	0.1070	1	13	43	R.VPSIAIDKTSGCQIYLSK.D	Carbamidomethyl (C)
													**696.0742**	**2085.2007**	**3**	**2085.0895**	**0.1112**	**0**	**49**	**45**	**K.EFQNLVDQHITPFVALSK.K**	
													782.4296	2344.2670	3	2344.1468	0.1202	1	37	45	K.TSGCQIYLSKDSLETEIVSSK.S	Carbamidomethyl (C)
													**1199.1096**	**2396.2046**	**2**	**2396.1303**	**0.0743**	**0**	**53**	**45**	**K.SIASGVASSSSSSSPSSGAAGPSSASVK.E**	
													**816.8008**	**2447.3807**	**3**	**2447.2544**	**0.1263**	**0**	**57**	**41**	**K.SATPAPASSAPAAPVAPAVSSTPVESK.K**	
													**847.1798**	**2538.5174**	**3**	**2538.4057**	**0.1117**	**0**	**63**	**41**	**K.KPSQETLLELIKPLNNFAAEVGK.I**	
													870.8012	2609.3818	3	2609.2762	0.1056	1	22	45	K.GVNQDQVDWVSNYVNFLKDLEK.Y	
Chp EC50	1617	66809891	XP_638669	DDB0233767	Protein phosphatase 2C-like domain-containing protein	DDB_G0284243	Q54PX6	44393/4.98	65360/5.00	6 (3)	6 (3)	13%	**464.6662**	**927.3179**	**2**	**927.4338**	**-0.1159**	**0**	**51**	**45**	**R.DFGVSFEK.N**	
													**471.7271**	**941.4396**	**2**	**941.5658**	**-0.1262**	**0**	**53**	**45**	**R.VNGILAVTR.S**	
													606.7266	1211.4387	2	1211.5935	-0.1548	0	23	41	K.YLYVANAGDAR.A	
													618.7220	1235.4294	2	1235.5717	-0.1423	0	15	40	R.IDAAGGFVCNGR.V	Carbamidomethyl(C)
													**635.2375**	**1268.4605**	**2**	**1268.6149**	**-0.1545**	**1**	**63**	**45**	**R.DFGVSFEKNAR.Y**	
													638.2555	1274.4965	2	1274.6507	-0.1542	0	41	43	K.SPEGDILELFR.N	
Chp EC50	3326	60475385	EAL73320	DDB0233903	Putative SAM dependent methyltransferase	DDB_G0267734	Q55GC0	33419/5.76	32460/5.97	2 (2)	2 (2)	9%	**592.3420**	**1182.6695**	**2**	**1182.6608**	**0.0087**	**1**	**71**	**45**	**K.AGNKDVLPEIK.E**	
													**772.4224**	**1542.8302**	**2**	**1542.8001**	**0.0301**	**0**	**65**	**45**	**R.DLAIDIGAGSGQATVR.L**	

Supplemental table 1 - Details about identified proteins

Treatment	Spot n°	ID by www.ncbi.nlm.nih.gov		ID by www.dictybase.org			UniPROT ID	MW(Da)/pI expected	MW(Da)/pI observed	n° matches	n° sequences	% coverage	mass					peptide				
		gi|	accession number	DDB	protein ID	gene name							observed	Mr(expt)	charge	Mr(calc)	delta	miss	score	ion score (identity)	sequence	modifications
Chp EC50	3424	66803080	XP_635383	DDB0230070	S-adenosylmethionine synthetase	metK	Q54F07	41737/6.14	49340/6.11	12 (9)	9 (6)	26%	486.7301	971.4456	2	971.5400	-0.0944	0	42	45	K.SLVAAGLADR.C	
													**514.7674**	**1027.5203**	**2**	**1027.6277**	**-0.1074**	**1**	**47**	**45**	**R.ADLKELVIK.A**	
													**676.2447**	**1350.4749**	**2**	**1350.6092**	**-0.1343**	**0**	**49**	**45**	**R.NDPDFTWETVK.E**	
													**679.8276**	**1357.6406**	**2**	**1357.7718**	**-0.1312**	**0**	**43**	**41**	**R.DLQLTRPIFQK.T**	
													**697.3024**	**1392.5902**	**2**	**1392.7357**	**-0.1455**	**0**	**67**	**47**	**K.TGMVMILGEITTK.A**	
													**705.2993**	**1408.5841**	**2**	**1408.7306**	**-0.1465**	**0**	**49**	**45**	**K.TGMVMILGEITTK.A**	**Oxidation (M)**
													**713.3043**	**1424.5941**	**2**	**1424.7255**	**-0.1314**	**0**	**62**	**45**	**K.TGMVMILGEITTK.A**	**2 Oxidation (M)**
													**732.2961**	**1462.5776**	**2**	**1462.7239**	**-0.1463**	**0**	**74**	**47**	**R.FVIGGPMGDSGLTGR.K**	
													**740.2970**	**1478.5794**	**2**	**1478.7188**	**-0.1394**	**0**	**68**	**45**	**R.FVIGGPMGDSGLTGR.K**	**Oxidation (M)**
													747.7598	1493.5050	2	1493.6674	-0.1624	1	30	41	K.IGYDDSSKGFDYK.T	
													804.3302	1606.6459	2	1606.8138	-0.1679	1	37	41	R.FVIGGPMGDSGLTGRK.I	Oxidation (M)
													**650.6102**	**1948.8089**	**3**	**1948.9432**	**-0.1343**	**0**	**55**	**47**	**K.IIIDSYGGWGAHGGGAFSGK.D**	
Chp EC50	3536	66818493	XP_642906	DDB0231504	Aldehyde dehydrogenase	DDB_G0276821	P83401	55155/5.78	60980/5.96	2 (2)	2 (2)	5%	**653.7964**	**1305.5783**	**2**	**1305.7041**	**-0.1258**	**0**	**67**	**45**	**R.AVLFASVGTTGQR.C**	
													**674.8157**	**1347.6169**	**2**	**1347.7609**	**-0.1440**	**0**	**78**	**45**	**K.GASTTSLITLAVSK.I**	
Chp EC50	3725	66801325	XP_629588	DDB0237783	Tetratricopeptide-like helical domain-containing protein (TPR) stress-induced-phosphoprotein 1	sti1	Q54DA8	63170/6.16	80380/6.08	3 (3)	3 (3)	6%	**692.9541**	**1383.8937**	**2**	**1383.7795**	**0.1142**	**0**	**47**	**41**	**K.ILSDPIMNQILK.D**	
													**742.4518**	**1482.8891**	**2**	**1482.7678**	**0.1213**	**0**	**95**	**41**	**R.IEANNPELLDLSR.K**	
													**856.4687**	**1710.9229**	**2**	**1710.8213**	**0.1016**	**0**	**58**	**45**	**R.EYQQALEVYDQGLR.I**	
Chp EC50	4432	66821393	XP_644181	DDB0185221	Beta-alanine synthase	pyd3	Q964D8	44088/6.32	44280/6.78	15 (7)	12 (5)	36%	393.6579	785.3012	2	785.3959	-0.0947	0	26	41	R.YELYAK.F	
													**540.2296**	**1078.4447**	**2**	**1078.5593**	**-0.1146**	**0**	**46**	**41**	**K.IAINICYGR.H**	**Carbamidomethyl (C)**
													**631.7666**	**1261.5186**	**2**	**1261.6190**	**-0.1004**	**0**	**44**	**43**	**K.QFESVQATLEK.Y**	**Gln->pyro-Glu (N-termQ)**
													639.2759	1276.5373	2	1276.6550	-0.1178	0	37	41	K.YIPAEELSEVK.R	
													**640.2685**	**1278.5224**	**2**	**1278.6455**	**-0.1232**	**0**	**71**	**45**	**K.QFESVQATLEK.Y**	
													438.1689	1311.4847	3	1311.6030	-0.1183	1	17	41	K.DKWNFQMTAR.Y	Oxidation (M)
													672.8045	1343.5945	2	1343.5533	0.0412	0	18	41	K.QFESVQATLEK.Y	2 Deamidated (NQ); Phospho (ST); Gln->pyro-Glu (N-termQ)
													**717.3246**	**1432.6346**	**2**	**1432.7561**	**-0.1215**	**1**	**50**	**45**	**K.YIPAEELSEVKR.I**	
													768.8312	1535.6479	2	1535.7831	-0.1352	1	18	42	M.SKQFESVQATLEK.Y	Acetyl (K)
													542.5669	1624.6789	3	1624.8316	-0.1528	1	22	41	R.KYNMVIISPMLER.D	2 Oxidation (M)
													**836.3322**	**1670.6499**	**2**	**1670.7834**	**-0.1335**	**0**	**52**	**42**	**R.NAAMTNNYFVGSINR.V**	
													**844.3277**	**1686.6408**	**2**	**1686.7783**	**-0.1375**	**0**	**105**	**45**	**R.NAAMTNNYFVGSINR.V**	**Oxidation (M)**
													**1065.9681**	**2129.9216**	**2**	**2130.0626**	**-0.1410**	**0**	**43**	**41**	**R.VSDGLNISEVDLNLCQQVK.D**	**Carbamidomethyl (C)**
													719.6220	2155.8442	3	2156.0425	-0.1983	1	18	41	R.EKYPWVEFAESASTGQSIK.F	
													920.0959	2757.2659	3	2757.4436	-0.1776	0	23	41	R.LGIIQNSIGAETTAPIQDQYLAIEAK.I	Deamidated (NQ)

Supplemental table 1 - Details about identified proteins

Treatment	Spot n°	ID by www.ncbi.nlm.nih.gov		ID by www.dictybase.org			UniPROT ID	MW(Da)/pI expected	MW(Da)/pI observed	n° matches	n° sequences	% coverage	mass					peptide				
		gi|	accession number	DDB	protein ID	gene name							observed	Mr(expt)	charge	Mr(calc)	delta	miss	score	ion score (identity)	sequence	modifications
Chp EC50	**4642**	66822027	XP_644368	DDB0266926	Transketolase	tkt-2	Q556J0	71912/6.39	74280/6.92	21 (10)	14 (8)	31%	**572.2671**	**1142.5197**	**2**	**1142.6295**	**-0.1098**	**0**	**60**	**45**	**R.ADGPTLLSLTR.Q**	
													648.7862	1295.5579	2	1295.6874	-0.1295	0	21	41	R.QNLPFLPGTAQK.K	Gln->pyro-Glu (N-termQ)
													**681.3010**	**1360.5875**	**2**	**1360.7351**	**-0.1476**	**0**	**54**	**41**	**R.GGYIVVSETAPLR.M**	
													702.8239	1403.6333	2	1403.7773	-0.1440	1	29	41	K.YVGLDGKVIGIDR.F	
													**727.2683**	**1452.5221**	**2**	**1452.6741**	**-0.1520**	**0**	**49**	**41**	**R.VVSMPCTELFDR.Q**	**Carbamidomethyl (C)**
													**735.2723**	**1468.5300**	**2**	**1468.6691**	**-0.1391**	**0**	**61**	**45**	**R.VVSMPCTELFDR.Q**	**Carbamidomethyl (C); Oxidation (M)**
													751.8019	1501.5891	2	1501.7275	-0.1384	0	29	42	R.IAMEAGVTSFWYK.Y	
													**759.7895**	**1517.5645**	**2**	**1517.7224**	**-0.1579**	**0**	**62**	**45**	**R.IAMEAGVTSFWYK.Y**	**Oxidation (M)**
													**783.8354**	**1565.6563**	**2**	**1565.8454**	**-0.1891**	**0**	**43**	**41**	**K.QIPEFPTTPIIATR.K**	**Gln->pyro-Glu (N-termQ)**
													792.3628	1582.7110	2	1582.8719	-0.1609	0	40	41	K.QIPEFPTTPIIATR.K	
													831.3459	1660.6773	2	1660.7978	-0.1205	1	19	40	K.QLGMTSENLVNISKQ.-	Deamidated (NQ); Oxidation (M); Gln->pyro-Glu (N-termQ)
													839.3320	1676.6494	2	1676.8403	-0.1909	1	20	40	K.QLGMTSENLVNISKQ.-	Oxidation(M)
													839.8313	1677.6481	2	1677.8243	-0.1762	1	17	41	K.QLGMTSENLVNISKQ.-	Deamidated (NQ); Oxidation (M)
													632.2686	1893.7841	3	1893.9441	-0.1600	1	15	41	K.VIGIDRFGMSAPGNAVMK.Q	2 Oxidation (M)
													**639.9581**	**1916.8524**	**3**	**1917.0320**	**-0.1796**	**1**	**48**	**45**	**R.EGTLRGGYIVVSETAPLR.M**	
													**652.9417**	**1955.8033**	**3**	**1955.9808**	**-0.1775**	**0**	**58**	**45**	**R.MILIATGSEVQHCVEAAK.L**	**Carbamidomethyl (C)**
													986.9082	1971.8018	2	1971.9758	-0.1740	0	15	40	R.MILIATGSEVQHCVEAAK.L	Carbamidomethyl (C); Oxidation (M)
													**658.2803**	**1971.8191**	**3**	**1971.9758**	**-0.1567**	**0**	**48**	**45**	**R.MILIATGSEVQHCVEAAK.L**	**Carbamidomethyl (C); Oxidation (M)**
													**671.3141**	**2010.9204**	**3**	**2011.0891**	**-0.1687**	**0**	**41**	**41**	**K.VSGATFLVFSDYLRPAIR.L**	
													731.3256	2190.9550	3	2191.1306	-0.1756	1	31	40	K.LAQLLESAHEKHEAIDIMK.Q	Oxidation (M)
													803.4000	3209.5709	4	3209.6833	-0.1124	1	35	41	K.AASGHLGLPLGSAEIGAALFGNS LIYNPKDTR.W	

Supplemental table 1 - Details about identified proteins

Treatment	Spot n°	ID by www.ncbi.nlm.nih.gov		ID by www.dictybase.org			UniPROT ID	MW(Da)/pI expected	MW(Da)/pI observed	n° matches	n° sequences	% coverage	mass					peptide				
		gi|	accession number	DDB	protein ID	gene name							observed	Mr(expt)	charge	Mr(calc)	delta	miss	score	ion score (identity)	sequence	modifications
Chp EC50	4655	66813780	XP_641069	DDB0214886	Succinate dehydrogenase (ubiquinone)	sdhA	Q9U3X4	68472/6.46	67690/6.73	11 (6)	9 (5)	20%	451.7293	901.4440	2	901.4756	-0.0316	0	39	41	K.EGVELIDK.C	
													575.2937	1148.5728	2	1148.5826	-0.0097	0	24	41	R.YAPSVADLASR.D	
													**575.2959**	**1148.5773**	**2**	**1148.5826**	**-0.0052**	**0**	**55**	**45**	**R.YAPSVADLASR.D**	
													**636.8571**	**1271.6997**	**2**	**1271.6721**	**0.0276**	**0**	**63**	**45**	**R.AVANEIENTLAK.D**	
													674.3688	1346.7230	2	1346.6830	0.0400	0	14	41	K.HTLSYLDVNTGK.V	
													**737.4274**	**1472.8402**	**2**	**1472.8350**	**0.0052**	**0**	**91**	**45**	**R.LGANSLLDIVVFGR.A**	
													**747.4162**	**1492.8178**	**2**	**1492.7997**	**0.0180**	**0**	**61**	**45**	**R.GVVAINLEDGTIHR.F**	
													**498.6147**	**1492.8222**	**3**	**1492.7997**	**0.0224**	**0**	**52**	**43**	**R.GVVAINLEDGTIHR.F**	
													804.9705	1607.9264	2	1607.8783	0.0481	0	29	41	R.TGHALLHTLYGQAVK.H	
													606.9696	1817.8869	3	1817.8478	0.0391	0	37	41	K.GLYAAGESACVSVHGANR.L	Carbamidomethyl (C)
													**819.1061**	**2454.2965**	**3**	**2454.2502**	**0.0462**	**0**	**48**	**45**	**K.DTPHKPLPPNAGEESIANIDAIR.F**	
		66807283	XP_637364	DDB0218901	Hypothetical protein DDBDRAFT_0218901	DDB_G0287201	Q54KR6	70872/5.93	67690/6.73	9 (4)	7 (4)	13%	**493.8028**	**985.5910**	**2**	**985.5807**	**0.0103**	**0**	**53**	**45**	**K.NLGLLEVTK.S**	
													**698.8961**	**1395.7777**	**2**	**1395.7609**	**0.0168**	**0**	**56**	**41**	**K.VLSTHEVIEIEK.V**	
													477.9194	1430.7363	3	1430.8020	-0.0658	0	13	41	R.IDKPIEITVFEK.E	
													728.8612	1455.7078	2	1455.6816	0.0261	0	43	45	K.SLPFFSLCENSR.G	Carbamidomethyl (C)
													**728.8662**	**1455.7179**	**2**	**1455.6816**	**0.0363**	**0**	**46**	**41**	**K.SLPFFSLCENSR.G**	**Carbamidomethyl (C)**
													744.3000	1486.5854	2	1486.7820	-0.1965	0	37	41	K.FTGLPFTHETIPK.K	
													496.6105	1486.8098	3	1486.7820	0.0278	0	27	42	K.FTGLPFTHETIPK.K	
													563.6600	1687.9581	3	1687.9396	0.0185	1	16	41	R.IDKPIEITVFEKEK.I	
													**794.1188**	**2379.3346**	**3**	**2379.2797**	**0.0549**	**0**	**46**	**41**	**K.YTELGGSVVHPLNENIIGLINK.L**	

Supplemental table 1 - Details about identified proteins

Treatment	Spot n°	ID by www.ncbi.nlm.nih.gov		ID by www.dictybase.org			UniPROT ID	MW(Da)/pI expected	MW(Da)/pI observed	n° matches	n° sequences	% coverage	mass					peptide				
		gi|	accession number	DDB	protein ID	gene name							observed	Mr(expt)	charge	Mr(calc)	delta	miss	score	ion score (identity)	sequence	modifications
Chp EC50	4730	66815641	XP_641837	DDB0229908	Putative iron regulatory protein	aco1	Q54X73	97929/6.03	87050/6.79	28 (12)	21 (11)	40%	**433.2646**	**864.5147**	**2**	**864.4705**	**0.0442**	**0**	**47**	**45**	**K.SFETILR.F**	
													**547.8084**	**1093.6023**	**2**	**1093.5590**	**0.0432**	**0**	**46**	**45**	**K.CVIAISFER.I**	**Carbamidomethyl (C)**
													**373.5760**	**1117.7062**	**3**	**1117.6495**	**0.0567**	**1**	**54**	**45**	**R.IEKLPYSIR.I**	
													655.8769	1309.7393	2	1309.6990	0.0403	0	38	43	K.EDLVVAGVLSGNR.N	Glu->pyro-Glu (N-termE)
													**664.8815**	**1327.7485**	**2**	**1327.7096**	**0.0389**	**0**	**58**	**47**	**K.EDLVVAGVLSGNR.N**	
													667.3550	1332.6955	2	1332.6561	0.0394	0	25	41	K.EQFNIELPTDK.S	
													450.9219	1349.7439	3	1349.6768	0.0671	0	15	40	K.STYIHNPPFFK.T	
													**709.3970**	**1416.7795**	**2**	**1416.7289**	**0.0506**	**0**	**52**	**45**	**K.VPTGLLYPWDEK.S**	
													**770.4361**	**1538.8577**	**2**	**1538.8093**	**0.0484**	**0**	**64**	**45**	**K.NAVEHGLEVAPYIK.T**	
													513.9652	1538.8738	3	1538.8093	0.0645	0	35	41	K.NAVEHGLEVAPYIK.T	
													793.3997	1584.7848	2	1584.7243	0.0605	0	15	40	K.QDFNSCLSSPVGFK.G	Carbamidomethyl (C)
													610.0047	1826.9923	3	1826.9275	0.0648	1	19	40	R.TVDALEQNQKIEFHR.N	
													**966.9986**	**1931.9826**	**2**	**1931.9265**	**0.0561**	**0**	**44**	**43**	**K.FVEFYGEGVSTLSVQDR.A**	
													1008.0749	2014.1352	2	2014.0921	0.0430	0	40	41	R.VLLQDFTGVPAVVDLAAMR.D	
													**672.3986**	**2014.1738**	**3**	**2014.0921**	**0.0817**	**0**	**56**	**46**	**R.VLLQDFTGVPAVVDLAAMR.D**	
													1016.0778	2030.1411	2	2030.0871	0.0541	0	35	40	R.VLLQDFTGVPAVVDLAAMR.D	Oxidation (M)
													**677.7299**	**2030.1677**	**3**	**2030.0871**	**0.0807**	**0**	**61**	**45**	**R.VLLQDFTGVPAVVDLAAMR.D**	**Oxidation (M)**
													725.7382	2174.1927	3	2174.1048	0.0879	1	21	40	K.SWNELKVPTGLLYPWDEK.S	
													**771.0616**	**2310.1628**	**3**	**2310.0876**	**0.0752**	**0**	**48**	**45**	**K.YQSEGHQLIVLAGSDYGSGSSR.D**	
													**772.1280**	**2313.3621**	**3**	**2313.2693**	**0.0928**	**0**	**54**	**45**	**K.INPLVPVDLVIDHSVQVDVSR.T**	
													675.1347	2696.5099	4	2696.3922	0.1177	1	43	45	K.DVENILNWENTANKVEIPFKPAR.V	
													908.1455	2721.4148	3	2721.3571	0.0577	0	25	41	K.LAPAVGPQTTYVPTGELMFISDAAEK.Y	Oxidation (M)
													914.5128	2740.5165	3	2740.4582	0.0583	0	22	40	R.SNLVGMGIIPLQFQPGQNASTLGLTGK.E	
													919.8458	2756.5155	3	2756.4531	0.0624	0	29	40	R.SNLVGMGIIPLQFQPGQNASTLGLTGK.E	Oxidation (M)
													**924.8403**	**2771.4990**	**3**	**2771.4052**	**0.0938**	**0**	**64**	**45**	**K.SQSHPIFTTTMELDLSTVVPSLSGPK.R**	
													930.1718	2787.4934	3	2787.4001	0.0933	0	29	40	K.SQSHPIFTTTMELDLSTVVPSLSGPK.R	Oxidation (M)
													1042.9032	3125.6876	3	3125.6087	0.0789	0	32	41	K.WGAQAFDGLFIAPPGSGIVHQVNLEYIAR.E	
													817.6986	3266.7653	4	3266.6466	0.1187	0	15	41	K.RPAITNAYCLLNLGDSITTDHISPAGNINR.K	Carbamidomethyl (C)
Chp EC50	6523	66825303	XP_646006	DDB0191502	Annexin VII	nxnA	P24639	49395/7.75	55270/8.04	4 (3)	3 (3)	11%	**749.8410**	**1497.6675**	**2**	**1497.8191**	**-0.1516**	**0**	**53**	**45**	**R.SLPHIAAVASEYIK.H**	
													912.3581	1822.7017	2	1822.8949	-0.1931	1	21	41	K.DLIQDIKSETSGNFEK.C	
													**608.5823**	**1822.7250**	**3**	**1822.8949**	**-0.1698**	**1**	**54**	**43**	**K.DLIQDIKSETSGNFEK.C**	
													**635.8985**	**1904.6737**	**3**	**1904.8687**	**-0.1950**	**1**	**48**	**43**	**K.NSLAHDIQADCSGDFKK.L**	**Carbamidomethyl (C)**

Supplemental table 1 - Details about identified proteins

Treatment	Spot n°	ID by www.ncbi.nlm.nih.gov		ID by www.dictybase.org			UniPROT ID	MW(Da)/pI expected	MW(Da)/pI observed	n° matches	n° sequences	% coverage	mass					peptide				
		gi|	accession number	DDB	protein ID	gene name							observed	Mr(expt)	charge	Mr(calc)	delta	miss	score	ion score (identity)	sequence	modifications
Chp EC50	7323	66811048	XP_639231	DDB0231355	Phosphopyruvate hydratase	enoA	Q54RK5	46961/5.67	41590/8.59	36 (14)	28 (15)	70%	**368.7250**	**735.4354**	**2**	**735.3915**	**0.0439**	**0**	**43**	**41**	**K.LGDLYR.E**	
													374.2473	746.4801	2	746.4326	0.0475	0	16	41	R.NLPLYK.Y	
													396.7436	791.4726	2	791.4290	0.0436	0	38	41	K.YNQLVR.I	
													**397.2336**	**792.4527**	**2**	**792.4130**	**0.0397**	**0**	**46**	**45**	**K.YNQLVR.I**	**Deamidated (NQ)**
													**401.2629**	**800.5113**	**2**	**800.4643**	**0.0469**	**0**	**59**	**45**	**K.EGLELLK.L**	
													**404.7533**	**807.4920**	**2**	**807.4491**	**0.0429**	**0**	**41**	**41**	**K.AGYTGLVK.I**	
													**451.7827**	**901.5508**	**2**	**901.5055**	**0.0453**	**0**	**41**	**41**	**K.ACNALLLK.V**	**Carbamidomethyl (C)**
													457.7265	913.4385	2	913.3930	0.0455	0	39	41	K.DFNEAYR.M	
													480.2905	958.5665	2	958.5195	0.0469	1	32	40	K.AREILNSR.G	Deamidated (NQ)
													**491.2859**	**980.5572**	**2**	**980.5178**	**0.0394**	**0**	**52**	**47**	**K.YISEIAGTK.M**	
													515.8311	1029.6476	2	1029.6004	0.0471	1	20	40	K.KACNALLLK.V	Carbamidomethyl (C)
													552.8542	1103.6938	2	1103.6451	0.0487	1	20	40	R.LAKYNQLVR.I	
													597.3026	1192.5907	2	1192.5546	0.0360	0	18	40	R.MGSEVYHNLK.N	Oxidation (M)
													597.8396	1193.6647	2	1193.5387	0.1261	0	17	41	R.MGSEVYHNLK.N	Deamidated (NQ); Oxidation (M)
													**622.3275**	**1242.6404**	**2**	**1242.5928**	**0.0476**	**0**	**57**	**41**	**K.NASWGVMVSHR.S**	
													622.7889	1243.5633	2	1243.5213	0.0420	0	29	42	K.IGMDCAASEFK.V	Carbamidomethyl (C); Oxidation (M)
													**630.7966**	**1259.5786**	**2**	**1259.5717**	**0.0069**	**0**	**65**	**47**	**K.NASWGVMVSHR.S**	**Deamidated (NQ); Oxidation (M)**
													**644.8752**	**1287.7359**	**2**	**1287.6935**	**0.0424**	**1**	**45**	**43**	**R.AGAADRNLPLYK.Y**	
													**651.8769**	**1301.7393**	**2**	**1301.6939**	**0.0454**	**0**	**66**	**45**	**K.VNQIGSVTESIR.A**	
													697.4453	1392.8760	2	1392.8340	0.0420	0	22	41	K.NILEVIQPAVIGK.S	
													697.9412	1393.8679	2	1393.8180	0.0498	0	14	40	K.NILEVIQPAVIGK.S	Deamidated (NQ)
													732.8922	1463.7698	2	1463.7144	0.0554	0	16	40	R.GNPTVEVDLYTEK.D	
													**774.4366**	**1546.8586**	**2**	**1546.8251**	**0.0335**	**0**	**47**	**43**	**K.LAMQEFMILPVGAK.D**	
													782.9533	1563.8921	2	1563.8040	0.0880	0	18	40	K.LAMQEFMILPVGAK.D	Deamidated (NQ); Oxidation (M)
													790.4393	1578.8641	2	1578.8150	0.0492	0	18	40	K.LAMQEFMILPVGAK.D	2 Oxidation (M)
													569.3834	1705.1284	3	1705.0502	0.0783	1	22	40	K.AIKNILEVIQPAVIGK.S	
													**895.9913**	**1789.9681**	**2**	**1789.9210**	**0.0471**	**0**	**49**	**45**	**R.AAVPSGASTGIYEAVELR.D**	
													598.3424	1792.0055	3	1791.9268	0.0787	0	32	41	R.LPVPAFNVINGGSHAGNK.L	Deamidated (NQ)
													**636.6837**	**1907.0292**	**3**	**1906.9384**	**0.0908**	**1**	**63**	**45**	**K.NNDGSAVISGEKLGDLYR.E**	
													**659.6878**	**1976.0417**	**3**	**1975.9388**	**0.1029**	**0**	**51**	**43**	**R.INEELGENHNYAGLTFR.K**	
													694.0651	2079.1736	3	2079.0684	0.1052	1	17	41	K.MRLPVPAFNVINGGSHAGNK.L	Deamidated (NQ)
													699.3929	2095.1568	3	2095.0633	0.0935	1	26	40	K.MRLPVPAFNVINGGSHAGNK.L	Deamidated (NQ); Oxidation (M)

													1134.6304	2267.2463	2	2267.2009	0.0454	0	13	40	K.LTASVDIQIVGDDLLVTNPER.I	
													759.7305	2276.1696	3	2276.0709	0.0986	0	17	41	R.YGQDAINVGDEGGFAPPIQSNK.E	
													771.4168	2311.2286	3	2311.1332	0.0954	1	42	42	R.GNPTVEVDLYTEKDGVVSSFR.A	
													1036.5187	3106.5344	3	3106.4335	0.1009	1	7	40	R.EFIKEYPIISIEDPFDQDDWESYTK.L	
Chp EC50	8432	66817212	XP_642484	DDB0231241	60S ribosomal protein L4	rpl4	Q54Z69	40221/11.58	40190/9.16	7 (6)	7 (6)	35%	627.8361	1253.6576	2	1253.6728	-0.0152	0	54	42	R.NIPGVEVANVSR.L	
													657.8615	1313.7084	2	1313.6939	0.0145	0	67	42	R.LVNSDEIQAAVR.A	
													824.9019	1647.7893	2	1647.7781	0.0112	0	75	41	K.SAFEQLDSTFGTFAK.S	
													930.5027	1858.9909	2	1859.0339	-0.0430	0	73	44	R.YAVVSALAASAVPALVMAR.G	
													1129.5262	2257.0378	2	2257.0036	0.0342	0	62	41	R.QAYGAAPYAGEQTSAESWGTGR.A	
													654.6408	2614.5339	4	2614.4595	0.0744	0	49	43	K.LPNVLTTPIRPDLVNFVHTNLNK.N	
													711.4062	2841.5955	4	2841.6229	-0.0274	1	41	44	K.VKLPNVLTTPIRPDLVNFVHTNLNK.N	
Mix EC25	0510	66806459	XP_636952	DDB0187751	Hypothetical protein DDBDRAFT_0187751	DDB_ G0288041	Q54JH9	336221/5.39	65810/4.81	5 (4)	5 (4)	3%	556.2793	1110.5257	2	1110.5413	-0.0156	1	45	41	K.KMLCSSQGLK.Y	Deamidated (NQ); Oxidation (M)
													623.7254	1245.4859	2	1245.5601	-0.0742	0	66	47	R.NGYTFSCWIR.T	
													979.0832	1956.1502	2	1956.1660	-0.0158	1	58	45	K.SINGIDKLFQLLLILEK.K	
													769.0325	2304.0759	3	2304.1110	-0.0351	1	29	41	K.FAFSECGSWNHVVLTHSKQK.I	
													792.7501	2375.2302	3	2375.2525	-0.0223	1	61	45	K.ITLYVNGKFIQNYSTLNYPK.S	
Mix EC25	1114	66818919	XP_643119	DDB0229442	Pyruvate dehydrogenase E1 beta subunit	pdhB	Q86HX0	39043/5.69	31550/5.42	3 (3)	3 (3)	16%	782.9008	1563.7870	2	1563.7636	0.0234	0	60	45	R.IVSNCMEAAEILAK.E	Carbamidomethyl (C); Oxidation (M)
													596.9767	1787.9082	3	1787.8537	0.0545	1	52	47	R.DAINSALDEELARDEK.V	
													952.1539	2853.4399	3	2853.3657	0.0742	0	59	45	R.GPNGPPTAVGAQHSQCFAAWYGSVPGLK.V	Carbamidomethyl (C)
Mix EC25	1119	60464861	EAL62977	DDB0237965	Putative delta-24-sterol methyltransferase	smt1	Q54I98	39819/6.16	38150/5.20	12 (8)	9 (7)	37%	545.7840	1089.5535	2	1089.6434	-0.0899	0	61	45	K.FTQLLEIVK.L	
													546.2775	1090.5405	2	1090.6274	-0.0869	0	38	41	K.FTQLLEIVK.L	Deamidated (NQ)
													700.7983	1399.5820	2	1399.6983	-0.1163	0	60	45	K.FNPEDPVEVNIK.K	
													479.8728	1436.5965	3	1436.7048	-0.1083	1	57	45	R.HKYESFEASIAR.H	
													646.5804	1936.7195	3	1936.8634	-0.1439	0	41	43	R.HEMYMAHQLGLFPGMK.V	3 Oxidation (M)
													1011.9108	2021.8071	2	2021.9919	-0.1848	0	67	45	R.FSGANVVGLNNNEYQIQR.G	
													675.9742	2024.9008	3	2025.0394	-0.1386	0	47	45	R.IVKPGGLFGGYEWIMTNK.F	Oxidation (M)
													1144.0534	2286.0922	2	2286.2471	-0.1548	0	28	40	K.QIELGNGLPDLVKPAEIINAAK.A	Deamidated (NQ); Gln->pyro-Glu (N-termQ)
													768.3878	2302.1417	3	2302.2896	-0.1479	0	58	47	K.QIELGNGLPDLVKPAEIINAAK.A	
													768.7235	2303.1488	3	2303.2736	-0.1248	0	50	45	K.QIELGNGLPDLVKPAEIINAAK.A	Deamidated (NQ)
													811.4068	2431.1986	3	2431.3686	-0.1699	1	48	45	K.KQIELGNGLPDLVKPAEIINAAK.A	Deamidated (NQ)
													917.0855	2748.2346	3	2748.3871	-0.1525	0	39	42	K.LAPAGSYNTNVWLQNAATFLVQGGEK.Q	
Mix EC25	2722	66818048	XP_642717	DDB0185029	Clathrin heavy chain	chcA	P25870	193477/5.45	96480/5.70	2 (2)	2 (2)	1%	555.8417	1109.6688	2	1109.6597	0.0091	0	74	47	K.LLLPWLEAR.V	
													657.3579	1312.7012	2	1312.6775	0.0237	0	60	45	K.VLYTNISNFSR.L	

Supplemental table 1 - Details about identified proteins

Treatment	Spot n°	ID by www.ncbi.nlm.nih.gov		ID by www.dictybase.org			UniPROT ID	MW(Da)/pI expected	MW(Da)/pI observed	n° matches	n° sequences	% coverage	mass					peptide				
		gi|	accession number	DDB	protein ID	gene name							observed	Mr(expt)	charge	Mr(calc)	delta	miss	score	ion score (identity)	sequence	modifications
Mix EC25	2803	134047850	P08799	DDB0191444	Myosin II heavy chain	mhcA	P08799	244124/5.47	99350/5.70	6 (5)	6 (5)	5%	542.2899	1623.8660	3	1623.8508	0.0152	1	27	43	R.IIQQNLRAYIDFK.S	3 Deamidated (NQ)
													**576.9783**	**1727.9339**	**3**	**1727.9305**	**0.0034**	**2**	**59**	**45**	**K.LELEAEQKAKQALEK.K**	**Deamidated (NQ)**
													**674.8245**	**1347.6480**	**2**	**1347.6379**	**0.0101**	**1**	**61**	**47**	**K.RVNDGQSDTISR.L**	**Deamidated (NQ)**
													**697.3540**	**2089.0601**	**3**	**2089.0174**	**0.0427**	**1**	**46**	**45**	**K.TAESALESLKDEIDAANNAK.A**	
													**848.7500**	**2543.2448**	**3**	**2543.1445**	**0.1003**	**2**	**53**	**45**	**K.YEEELEEMKRVNDGQSDTISR.L**	**Oxidation (M)**
													**959.5402**	**1917.0783**	**2**	**1917.0207**	**0.0576**	**0**	**57**	**45**	**K.TALNAASTVFGVNPSVLEK.A**	
Mix EC25	3110	38637654	DAA01127	DDB0215363	Aldehyde reductase	alrA	Q6IMN8	33855/5.95	29940/6.39	15 (9)	14 (9)	55%	**863.9285**	**1725.9057**	**2**	**1725.9565**	**-0.0508**	**1**	**51**	**45**	**K.LSSGHKIPLVGFGTWK.A**	
													**559.2486**	**1116.5296**	**2**	**1116.6331**	**-0.1035**	**0**	**56**	**45**	**K.IPLVGFGTWK.A**	
													**959.0392**	**1916.0586**	**2**	**1916.0770**	**-0.0184**	**1**	**63**	**47**	**K.IPLVGFGTWKAETTLVGK.A**	
													716.8569	1431.7316	2	1431.6816	0.0500	0	27	41	R.HIDCAAVYLNEK.E	
													765.3931	2293.1704	3	2293.1048	0.0656	1	39	43	R.HIDCAAVYLNEKEVGEAFTK.K	
													986.5225	1971.0705	2	1971.0312	0.0393	2	41	43	K.FTTEATVKREDVFITSK.L	
													**674.3374**	**2019.9823**	**3**	**2020.0530**	**-0.0707**	**2**	**58**	**47**	**K.REDVFITSKLWNTFHK.K**	
													**997.0221**	**1992.0442**	**2**	**1992.0468**	**-0.0026**	**2**	**64**	**47**	**R.EDVFITSKLWNTFHKK.E**	
													**938.9679**	**1875.9358**	**2**	**1875.9287**	**0.0071**	**1**	**45**	**45**	**R.ETWEEMEKLVDAGLVK.S**	
													733.3836	2197.1507	3	2197.1200	0.0307	0	42	45	K.SIGLSNFNVQGLMEVLSYAR.I	
													738.7093	2213.1279	3	2213.1149	0.0130	0	25	40	K.SIGLSNFNVQGLMEVLSYAR.I	Oxidation (M)
													**753.7359**	**2258.2792**	**3**	**2258.2422**	**0.0370**	**0**	**57**	**45**	**R.IKPAANQVELHPFLSQPELK.K**	
													796.4263	2386.2998	3	2386.3372	-0.0374	1	41	47	R.IKPAANQVELHPFLSQPELKK.F	
													**715.8422**	**1429.7568**	**2**	**1429.8081**	**-0.0513**	**1**	**64**	**47**	**K.WAIQKNFSVIPK.S**	
													**826.3505**	**1650.7381**	**2**	**1650.7864**	**-0.0483**	**1**	**66**	**46**	**R.TCDPAKFWGVPLFN.-**	

Supplemental table 1 - Details about identified proteins

Treatment	Spot n°	ID by www.ncbi.nlm.nih.gov		ID by www.dictybase.org			UniPROT ID	MW(Da)/pI expected	MW(Da)/pI observed	n° matches	n° sequences	% coverage	mass					peptide				
		gi|	accession number	DDB	protein ID	gene name							observed	Mr(expt)	charge	Mr(calc)	delta	miss	score	ion score (identity)	sequence	modifications
Mix EC25	3414	66818493	XP_642906	DDB0231504	Aldehyde dehydrogenase	DDB_ G0276821	P83401	55155/5.78	60720/6.34	15 (9)	15 (9)	38%	**453.7508**	**905.4870**	**2**	**905.4892**	**-0.0022**	**0**	**51**	**45**	**K.LISLEMGK.I**	**Oxidation (M)**
													**456.2619**	**910.5093**	**2**	**910.5025**	**0.0069**	**0**	**46**	**45**	**K.WALTPAPR.R**	
													465.7537	929.4928	2	929.4970	-0.0043	0	31	43	K.WGGAGEIIK.C	
													472.2840	942.5534	2	942.5386	0.0149	1	27	40	R.EKIEPLSK.L	
													597.8039	1193.5932	2	1193.5815	0.0116	0	24	40	K.EFTEGLEEIK.K	
													**653.8682**	**1305.7218**	**2**	**1305.7040**	**0.0178**	**0**	**47**	**45**	**R.AVLFASVGTTGQR.C**	
													**661.8575**	**1321.7005**	**2**	**1321.6765**	**0.0240**	**1**	**58**	**45**	**K.EFTEGLEEIKK.Q**	
													**674.8000**	**1347.5854**	**2**	**1347.7609**	**-0.1755**	**0**	**83**	**47**	**K.GASTTSLITLAVSK.I**	
													**735.9037**	**1469.7928**	**2**	**1469.7514**	**0.0414**	**0**	**55**	**47**	**R.FGLISFTGSTEVGR.R**	
													741.9390	1481.8634	2	1481.8203	0.0430	0	15	40	K.TELFVPILYIMK.F	Oxidation (M)
													578.9789	1733.9148	3	1733.8988	0.0160	0	38	40	R.LFVHESLYDTILER.L	
													**1030.0197**	**2058.0249**	**2**	**2057.9364**	**0.0886**	**0**	**69**	**47**	**K.GEVQEFIDVCDYATGLSR.S**	**Carbamidomethyl (C)**
													720.0905	2157.2497	3	2157.1793	0.0704	0	41	43	K.IGNPLEEGVLVGPLHTQSAVK.E	
													**840.8042**	**2519.3909**	**3**	**2519.3271**	**0.0638**	**0**	**47**	**43**	**K.LDISGGNFVEPTVVAIEHDAPI VK.T**	
													**846.4895**	**2536.4468**	**3**	**2536.3747**	**0.0720**	**0**	**45**	**43**	**K.TILELGGNNAIVVAEDADIEL VLR.A**	
Mix EC25	3416	22711882	AAG34561	DDB0191349	Phosphoglycerate kinase	pgkA	Q9GPM4	45681/6.01	56650/6.53	9 (6)	9 (6)	31%	669.9366	1337.8592	2	1337.8030	0.0562	1	38	43	K.VLEDQLKRPIK.F	
													**691.3804**	**1380.7404**	**2**	**1380.7183**	**0.0221**	**0**	**52**	**45**	**R.AHSSMVGINLPQK.A**	
													**739.4193**	**1476.8438**	**2**	**1476.7435**	**0.1003**	**0**	**57**	**45**	**K.TIVWNGPMGVFEK.S**	
													**761.9612**	**1521.8524**	**2**	**1521.7748**	**0.0776**	**0**	**63**	**47**	**R.IDASIPTLEYCLK.N**	
													**802.4385**	**1602.8174**	**2**	**1602.7161**	**0.1013**	**0**	**66**	**45**	**R.FHIEEEGSGVDAEGK.K**	
													813.9641	1625.9242	2	1625.8300	0.0942	1	26	41	K.FIDNKEIGSSLFEK.T	
													**817.9525**	**1633.8705**	**2**	**1633.7848**	**0.0857**	**0**	**54**	**47**	**K.LGDVYVNDAFGTAHR.A**	
													850.0125	1698.0041	2	1697.9715	0.0326	0	45	47	K.ALESPSKPFLAILGGAK.V	
													**778.7567**	**2333.2247**	**3**	**2333.1691**	**0.0556**	**1**	**62**	**47**	**K.LHFPVDYVIADKFDNDANIK. T**	

Supplemental table 1 - Details about identified proteins

Treatment	Spot n°	ID by www.ncbi.nlm.nih.gov		ID by www.dictybase.org			UniPROT ID	MW(Da)/pI expected	MW(Da)/pI observed	n° matches	n° sequences	% coverage	mass					peptide				
		gi|	accession number	DDB	protein ID	gene name							observed	Mr(expt)	charge	Mr(calc)	delta	miss	score	ion score (identity)	sequence	modifications
Mix EC25	4613	66808101	XP_637773	DDB0234127	Glucosamine-6-phosphate isomerase	nagB1	Q54M58	82645/6.14	85390/6.67	20 (9)	18 (7)	28%	421.7713	841.5280	2	841.4810	0.0470	0	38	42	K.WSPLLAR.K	
													429.7782	857.5419	2	857.4970	0.0448	0	64	47	K.AILSAIDR.L	
													550.3665	1098.7184	2	1098.6761	0.0423	1	44	45	K.AILSAIDRLK.T	
													554.8281	1107.6417	2	1107.5998	0.0419	0	49	45	R.IILMAFSEGK.A	
													562.8297	1123.6448	2	1123.5947	0.0501	0	47	45	R.IILMAFSEGK.A	Oxidation (M)
													574.2959	1146.5772	2	1146.5346	0.0426	0	41	41	R.FANFASEFSK.L	
													625.8431	1249.6717	2	1249.6302	0.0415	0	59	47	K.LFSLGNESVER.S	
													669.3668	1336.7190	2	1336.6623	0.0567	0	38	40	R.IGFNESGSLANTK.T	
													448.9309	1343.7708	3	1343.7157	0.0551	1	32	40	K.TRLVDLEQNTR.I	
													498.6129	1492.8169	3	1492.7634	0.0535	1	35	40	K.RIGFNESGSLANTK.T	
													512.6297	1534.8671	3	1534.7991	0.0681	1	31	41	R.SNTIEKNVNEFIK.T	
													584.0025	1748.9856	3	1748.9138	0.0719	0	66	45	R.IHFLDLPFYETGAVK.K	
													600.6746	1799.0018	3	1798.9175	0.0844	1	26	41	R.YMQENLFELIDIKK.E	Oxidation (M)
													931.0079	1860.0012	2	1859.9629	0.0383	0	49	43	K.TTEGEITPAIPSTIFQR.H	
													623.3822	1867.1249	3	1867.0601	0.0648	1	49	45	K.IEQVGGIDLMLIPIGKR.I	Oxidation (M)
													936.4863	1870.9581	2	1870.9101	0.0480	0	20	41	K.NVITFNVDEYYPIER.N	
													678.0036	2030.9889	3	2030.9255	0.0634	1	16	41	K.INSETGLFECNEEFKSK.E	Carbamidomethyl (C)
													768.0826	2301.2260	3	2301.1464	0.0796	0	37	41	R.VIVFSPHPDDDVISMGGTFIR.L	
													773.4118	2317.2135	3	2317.1413	0.0722	0	54	47	R.VIVFSPHPDDDVISMGGTFIR.L	Oxidation (M)
													645.6165	2578.4370	4	2578.3220	0.1150	1	17	40	R.YAGVAPDRIHFLDLPFYETGAVK.K	
Mix EC25	4615	166240434	XP_640392	DDB0235163	Putative transport protein	sec23	Q54T59	84646/6.07	83890/6.63	4 (3)	4 (3)	10%	723.8612	1445.7199	2	1445.7297	-0.0098	1	63	47	K.VGSPETMNLQSKR.F	
													912.9215	1823.8025	2	1823.8360	-0.0335	0	54	47	K.TSCVGENEIGIGGTSSWK.V	
													753.3508	2257.0246	3	2257.0831	-0.0585	0	37	43	R.ATGMAFSVANALLSTVASNMGGR.I	2 Oxidation (M)
													655.7254	2619.1397	4	2619.2138	-0.0741	0	58	45	K.LTGGYMVLADSFDHPMFTQSFQK.I	

Supplemental table 1 - Details about identified proteins

Treatment	Spot n°	ID by www.ncbi.nlm.nih.gov		ID by www.dictybase.org			UniPROT ID	MW(Da)/pI expected	MW(Da)/pI observed	n° matches	n° sequences	% coverage	mass					peptide				
		gi|	accession number	DDB	protein ID	gene name							observed	Mr(expt)	charge	Mr(calc)	delta	miss	score	ion score (identity)	sequence	modifications
Mix EC25	5208	60462281	EAL60507	DDB0231294	Isocitrate dehydrogenase (NAD+)	idhB	Q54B68	38891/8.48	38830/7.67	16 (8)	14 (7)	44%	428.2565	854.4985	2	854.4974	0.0011	0	33	41	K.QIPGITAR.H	
													**446.2133**	**890.4120**	**2**	**890.4174**	**-0.0054**	**0**	**46**	**43**	**R.YAFEYAK.A**	
													**539.2787**	**1076.5428**	**2**	**1076.5349**	**0.0079**	**0**	**50**	**45**	**K.EGTLTSDIGGK.S**	
													560.2995	1118.5844	2	1118.5832	0.0012	0	25	41	K.HLGLNEHATK.V	
													683.8610	1365.7074	2	1365.6816	0.0258	0	28	41	K.QFTGAVIDYIEK.N	Gln->pyro-Glu (N-termQ)
													**692.3767**	**1382.7389**	**2**	**1382.7081**	**0.0308**	**0**	**51**	**45**	**K.QFTGAVIDYIEK.N**	
													693.3800	1384.7454	2	1384.7173	0.0282	0	30	41	K.ALDLYAHVVPCK.Q	Carbamidomethyl (C)
													**476.2625**	**1425.7658**	**3**	**1425.7616**	**0.0042**	**0**	**54**	**47**	**R.HDDVLVDFVVIR.E**	
													**713.8991**	**1425.7836**	**2**	**1425.7616**	**0.0221**	**0**	**59**	**47**	**R.HDDVLVDFVVIR.E**	
													**744.9210**	**1487.8274**	**2**	**1487.8195**	**0.0080**	**1**	**52**	**47**	**K.AVIKEGTLTSDIGGK.S**	
													754.4057	1506.7969	2	1506.7678	0.0291	0	36	40	K.GPLYTEILSGSQSR.N	
													795.9400	1589.8654	2	1589.8520	0.0133	0	27	40	K.ANPTGLLLASVMMLK.H	2 Oxidation (M)
													612.0151	1833.0236	3	1832.9739	0.0496	1	25	40	K.DKANPTGLLLASVMMLK.H	2 Oxidation (M)
													**640.3706**	**1918.0900**	**3**	**1918.0523**	**0.0377**	**1**	**53**	**45**	**K.VALKGPLYTEILSGSQSR.N**	
													**826.1040**	**2475.2902**	**3**	**2475.2492**	**0.0410**	**0**	**47**	**45**	**R.ENTQGEYSGLEQVLTPGVVQSLK.I**	
													864.1626	2589.4659	3	2589.3359	0.1300	0	39	41	K.TVTVIPGDGIGPEITSSVMGVFQAAK.V	Oxidation (M)
Mix EC25	5307	60463691	EAL61873	DDB0230070	S-adenosyl-methionine synthetase	metK	Q54F07	42079/6.14	46310/7.10	8 (5)	6 (4)	19%	**486.7229**	**971.4313**	**2**	**971.5399**	**-0.1086**	**0**	**57**	**45**	**K.SLVAAGLADR.C**	
													**676.2448**	**1350.4751**	**2**	**1350.6092**	**-0.1341**	**0**	**46**	**45**	**R.NDPDFTWETVK.E**	
													679.8260	1357.6375	2	1357.7718	-0.1343	0	39	41	R.DLQLTRPIFQK.T	
													**697.3111**	**1392.6077**	**2**	**1392.7356**	**-0.1279**	**0**	**55**	**47**	**K.TGMVMILGEITTK.A**	
													**713.2997**	**1424.5849**	**2**	**1424.7254**	**-0.1405**	**0**	**58**	**47**	**K.TGMVMILGEITTK.A**	**2 Oxidation (M)**
													**740.2956**	**1478.5767**	**2**	**1478.7187**	**-0.1420**	**0**	**49**	**47**	**R.FVIGGPMGDSGLTGR.K**	**Oxidation (M)**
													498.8474	1493.5203	3	1493.6674	-0.1471	1	29	40	K.IGYDDSSKGFDYK.T	
													747.7755	1493.5365	2	1493.6674	-0.1309	1	22	40	K.IGYDDSSKGFDYK.T	
Mix EC25	5415	66813238	XP_640798	DDB0230052	3-phosphoglycerate dehydrogenase	serA	Q54UH8	44720/6.61	54160/7.30	2 (2)	2 (2)	8%	**858.9617**	**1715.9089**	**2**	**1715.9491**	**-0.0402**	**0**	**67**	**45**	**R.SVAELIICEIITLSR.K**	Carbamidomethyl (C)
													**1060.5388**	**2119.0631**	**2**	**2119.0909**	**-0.0278**	**0**	**61**	**47**	**R.DINNILSEFNVSAQVLSTR.K**	
Mix EC25	6016	60468343	EAL66350	DDB0215335	Actin binding protein	hatA	P13231	13505/6.89	15860/8.06	5 (3)	3 (2)	25%	**772.3401**	**1542.6657**	**2**	**1542.7361**	**-0.0704**	**0**	**52**	**45**	**R.NSAIWASGTMGHGVR.G**	
													**520.5611**	**1558.6614**	**3**	**1558.7311**	**-0.0697**	**0**	**63**	**45**	**R.NSAIWASGTMGHGVR.G**	**Oxidation (M)**
													780.3459	1558.6772	2	1558.7311	-0.0538	0	21	41	R.NSAIWASGTMGHGVR.G	Oxidation (M)
													**878.3686**	**1754.7226**	**2**	**1754.8145**	**-0.0919**	**0**	**44**	**41**	**R.LSMQEDGNLVIYDSR.N**	**Oxidation (M)**
													953.9711	1905.9276	2	1906.0234	-0.0957	0	13	40	K.LIMQNDGNLVLYIGSLK.S	Oxidation (M)

Supplemental table 1 - Details about identified proteins

Treatment	Spot n°	ID by www.ncbi.nlm.nih.gov		ID by www.dictybase.org			UniPROT ID	MW(Da)/pI expected	MW(Da)/pI observed	n° matches	n° sequences	% coverage	mass					peptide				
		gi|	accession number	DDB	protein ID	gene name							observed	Mr(expt)	charge	Mr(calc)	delta	miss	score	ion score (identity)	sequence	modifications
Mix EC25	6210	66820452	XP_643839	DDB0185122	G beta like protein	gpbB	P46800	36208/7.64	32900/7.91	4 (3)	4 (3)	15%	**557.8021**	**1113.5896**	**2**	**1113.6798**	**-0.0902**	**0**	**58**	**45**	**K.IIIWDLLTK.Q**	
													**612.7880**	**1223.5615**	**2**	**1223.7125**	**-0.1510**	**0**	**56**	**47**	**K.QVLAEIVPEVK.E**	
													817.8252	1633.6358	2	1633.8134	-0.1776	1	27	41	R.DATIKVWNTLGECK.F	Carbamidomethyl (C)
													**659.6268**	**1975.8585**	**3**	**1976.0004**	**-0.1419**	**1**	**63**	**45**	**R.FSPNTPTIVSGSWDNKVK.I**	
Mix EC25	6607	66805581	XP_636512	DDB0191139	Cyclase associated protein	cap	P54654	49611/6.97	73600/7.70	9 (7)	9 (7)	42%	**562.8284**	**1123.6460**	**2**	**1123.5873**	**0.0587**	**1**	**54**	**47**	**K.NFTDKSSVVK.A**	
													**604.3306**	**1206.6612**	**2**	**1206.5979**	**0.0633**	**0**	**57**	**47**	**K.DSLETEIVSSK.S**	
													**680.4076**	**1358.8121**	**2**	**1358.7558**	**0.0563**	**1**	**49**	**45**	**K.KGPGLGAVFGELSK.G**	
													**830.4659**	**1658.9318**	**2**	**1658.8839**	**0.0479**	**1**	**62**	**45**	**K.AIDAEKALINTASQSK.K**	
													660.7084	1979.1252	3	1979.0397	0.0855	1	37	41	R.VPSIAIDKTSGCQIYLSK.D	Carbamidomethyl (C)
													696.0456	2085.1320	3	2085.0895	0.0425	0	42	45	K.EFQNLVDQHITPFVALSK.K	
													**782.3905**	**2344.1600**	**3**	**2344.1468**	**0.0132**	**1**	**49**	**43**	**K.TSGCQIYLSKDSLETEIVSSK.S**	**Carbamidomethyl (C)**
													**816.7704**	**2447.3084**	**3**	**2447.2544**	**0.0540**	**0**	**63**	**43**	**K.SATPAPASSAPAAPVAPAVSSTPVESK.K**	
													**870.7896**	**2609.3542**	**3**	**2609.2762**	**0.0780**	**1**	**61**	**47**	**K.GVNQDQVDWVSNYVNFLKDLEK.Y**	
Mix EC25	6608	66816585	XP_642302	DDB0235360	ATP citrate synthase	acly	Q54YA0	67278/6.94	75850/7.69	4 (3)	4 (3)	14%	**844.4332**	**1686.8519**	**2**	**1686.9127**	**-0.0609**	**0**	**57**	**47**	**K.VIIGPATVGGIQAGCFK.I**	**Carbamidomethyl(C)**
													**628.3510**	**1882.0312**	**3**	**1882.0888**	**-0.0575**	**1**	**79**	**47**	**R.AGKDLVSSLVSGLLTIGPR.F**	
													**1194.0733**	**2386.1321**	**2**	**2386.1692**	**-0.0372**	**0**	**48**	**45**	**R.EAGAVVPTSFEDFSNVIAATYAK.L**	
													1114.2280	3339.6622	3	3339.7674	-0.1052	1	33	43	R.SAYQSSLLALREPSIQTVVIIAEGVPENEAR.S	

Supplemental table 1 - Details about identified proteins

Treatment	Spot n°	ID by www.ncbi.nlm.nih.gov		ID by www.dictybase.org			UniPROT ID	MW(Da)/pI expected	MW(Da)/pI observed	n° matches	n° sequences	% coverage	mass					peptide				
		gi|	accession number	DDB	protein ID	gene name							observed	Mr(expt)	charge	Mr(calc)	delta	miss	score	ion score (identity)	sequence	modifications
													**372.2567**	**742.4988**	**2**	**742.4953**	**0.0035**	**0**	**54**	**47**	**R.ISIIGLK.D**	
													407.7711	813.5277	2	813.5324	-0.0047	0	33	43	K.QLTLIVK.S	
													**429.7750**	**857.5354**	**2**	**857.5222**	**0.0132**	**0**	**58**	**45**	**R.VADILTVK.G**	
													468.7641	935.5137	2	935.5076	0.0061	0	23	41	K.AGSALNYIK.S	
													511.7642	1021.5139	2	1021.5080	0.0059	0	16	41	K.LNYEGIDAK.L	
													**536.7778**	**1071.5411**	**2**	**1071.5349**	**0.0063**	**0**	**51**	**45**	**K.TNNWPAELK.V**	
													**568.7884**	**1135.5623**	**2**	**1135.5621**	**0.0002**	**0**	**59**	**45**	**R.SNIANAANSFK.H**	
													624.8337	1247.6528	2	1247.6510	0.0018	0	30	40	K.FTITPGSEQIR.A	
													630.3150	1258.6155	2	1258.6081	0.0073	0	18	40	K.EVYDFLSTSAK.K	
													**427.5653**	**1279.6740**	**3**	**1279.6772**	**-0.0032**	**1**	**54**	**45**	**K.AGSALNYIKSEK.A**	
													**641.8465**	**1281.6784**	**2**	**1281.6565**	**0.0219**	**0**	**68**	**47**	**K.LTPPTGDELPSR.G**	
													438.2541	1311.7405	3	1311.7299	0.0106	1	36	45	R.GITYLKLHPDR.V	
													442.2779	1323.8119	3	1323.8126	-0.0006	1	45	47	R.ISIIGLKDLAPGK.Q	
													**458.2476**	**1371.7210**	**3**	**1371.7034**	**0.0176**	**0**	**59**	**47**	**K.IIYGHLEDPSTK.V**	
													462.9414	1385.8025	3	1385.7878	0.0147	1	19	40	K.ISGDDRISIIGLK.D	
													465.9617	1394.8633	3	1394.8497	0.0136	1	22	40	K.DLAPGKQLTLIVK.S	
													**483.2695**	**1446.7868**	**3**	**1446.7831**	**0.0037**	**1**	**58**	**47**	**K.AKFTITPGSEQIR.A**	
													**528.6384**	**1582.8933**	**3**	**1582.8871**	**0.0061**	**1**	**56**	**45**	**R.LQLLQPFAPWDKK.D**	
**Mix EC25**	**6612**	60470010	EAL67991	DDB0230168	Aconitase, mitochondrial	aco2	Q54XS2	83889/8.19	83600/7.93	37 (18)	32 (16)	42%	**529.9665**	**1586.8777**	**3**	**1586.8628**	**0.0149**	**1**	**61**	**45**	**R.SASVAQQALDKGITAK.A**	
													534.6444	1600.9112	3	1600.8858	0.0254	1	23	40	K.DLVDMQVLIKVQGK.C	Oxidation (M)
													539.5891	1615.7456	3	1615.7487	-0.0031	0	28	40	K.CTTDHISMAGPWLK.Y	Carbamidomethyl (C)
													544.9247	1631.7524	3	1631.7436	0.0088	0	33	40	K.CTTDHISMAGPWLK.Y	Carbamidomethyl (C); Oxidation (M)
													**549.6020**	**1645.7841**	**3**	**1645.7671**	**0.0171**	**0**	**61**	**47**	**K.ANHTMNAGQIEWFK.A**	
													554.9293	1661.7659	3	1661.7620	0.0039	0	35	40	K.ANHTMNAGQIEWFK.A	Oxidation (M)
													578.6338	1732.8796	3	1732.8784	0.0012	1	21	40	K.FAESVKTNNWPAELK.V	
													**586.3204**	**1755.9395**	**3**	**1755.9155**	**0.0239**	**1**	**60**	**47**	**K.IIYGHLEDPSTKVER.G**	
													**439.9969**	**1755.9584**	**4**	**1755.9155**	**0.0429**	**1**	**62**	**47**	**K.IIYGHLEDPSTKVER.G**	
													889.9461	1777.8776	2	1777.8887	-0.0110	0	25	39	K.QGILPLTFANPSDYDK.I	
													943.4673	1884.9200	2	1884.9339	-0.0139	0	28	39	K.VGGVVLANACGPCIGQWK.R	2 Carbamidomethyl (C)
													946.4134	1890.8121	2	1890.8088	0.0034	0	26	39	K.VGLIGSCTNSSYEDMSR.S	Carbamidomethyl (C); Oxidation (M)
													**969.4456**	**1936.8767**	**2**	**1936.8915**	**-0.0148**	**0**	**49**	**45**	**R.GVNWIVVGDENYGEGSSR.E**	
													**681.3589**	**2041.0548**	**3**	**2041.0350**	**0.0198**	**1**	**58**	**45**	**K.VGGVVLANACGPCIGQWKR.E**	**2 Carbamidomethyl (C)**

											1027.5486	2053.0826	2	2053.0956	-0.0130	0	35	41	K.ANAVLNQFTGEIGPVPTVAR.D	
											685.7026	2054.0861	3	2054.0797	0.0064	0	23	40	K.ANAVLNQFTGEIGPVPTVAR.D	Deamidated (NQ)
											**738.0320**	**2211.0741**	**3**	**2211.0589**	**0.0151**	**0**	**46**	**41**	**R.GHLDNISNNMLIGAINSENGK.A**	**Deamidated (NQ)**
											743.3635	2227.0688	3	2227.0539	0.0150	0	20	40	R.GHLDNISNNMLIGAINSENGK.A	Deamidated (NQ); Oxidation (M)
											**808.0706**	**2421.1901**	**3**	**2421.1812**	**0.0089**	**1**	**62**	**47**	**K.QGILPLTFANPSDYDKISGDDR.I**	
