# Supplementary material for: Effects of Nickel, Chlorpyrifos and Their Mixture on the Dictyostelium discoideum Proteome
Source: Int J Mol Sci. 2012 Nov 23;13(12):15679–705. doi: 10.3390/ijms131215679 (PMC3546656; doi:10.3390/ijms131215679)
Supplement: Supplementary file 5 [file ijms-13-15679-s005.pdf]

PDQuest vs 7.3.1 Software - Matching Summary

Treatment	Information	Control gels							Treated gels						
		1	2	3	4	5	6	7	1	2	3	4	5	6	7
Nickel EC25	n° spot x gel	496	492	499	495	491	497	482	492	493	495	491	487	496	487
	n° matched spots vs. member gel (%)	*98%*	*98%*	*97%*	*98%*	*98%*	*98%*	*95%*	*97%*	*97%*	*98%*	*98%*	*99%*	*98%*	*98%*
	n° matched spots vs. master gel (%)	*96%*	*94%*	*95%*	*93%*	*93%*	*95%*	*95%*	*97%*	*97%*	*95%*	*93%*	*93%*	*85%*	*95%*
	n° spots matched to every member	481													
Nickel EC50	n° spot x gel	483	473	468	461	481	475	471	488	493	467	454	478	484	472
	n° matched spots vs. member gel (%)	*97%*	*100%*	*99%*	*97%*	*97%*	*98%*	*97%*	*95%*	*94%*	*99%*	*100%*	*96%*	*97%*	*97%*
	n° matched spots vs. master gel (%)	*99%*	*100%*	*98%*	*95%*	*97%*	*97%*	*96%*	*98%*	*98%*	*98%*	*89%*	*96%*	*95%*	*97%*
	n° spots matched to every member	450													
Chlorpyrifos EC25	n° spot x gel	496	492	499	495	491	497	482	491	501	486	493	487	485	491
	n° matched spots vs. member gel (%)	*98%*	*98%*	*97%*	*98%*	*98%*	*98%*	*95%*	*98%*	*87%*	*98%*	*98%*	*97%*	*96%*	*97%*
	n° matched spots vs. master gel (%)	*96%*	*94%*	*95%*	*93%*	*93%*	*95%*	*95%*	*93%*	*95%*	*96%*	*94%*	*87%*	*100%*	*96%*
	n° spots matched to every member	481													
Chlorpyrifos EC50	n° spot x gel	572	575	576	573	564	573	568	571	574	575	570	544	572	573
	n° matched spots vs. member gel (%)	*98%*	*100%*	*100%*	*99%*	*100%*	*98%*	*97%*	*100%*	*99%*	*99%*	*100%*	*99%*	*97%*	*98%*
	n° matched spots vs. master gel (%)	*96%*	*99%*	*99%*	*99%*	*98%*	*98%*	*96%*	*94%*	*98%*	*99%*	*100%*	*99%*	*97%*	*96%*
	n° spots matched to every member	526													
Mixture	n° spot x gel	466	481	473	469	468	472	475	489	469	465	467	473	468	471
	n° matched spots vs. member gel (%)	*98%*	*96%*	*98%*	*98%*	*98%*	*98%*	*97%*	*99%*	*98%*	*98%*	*98%*	*100%*	*98%*	*98%*
	n° matched spots vs. master gel (%)	*96%*	*97%*	*97%*	*96%*	*96%*	*97%*	*97%*	*98%*	*96%*	*95%*	*97%*	*97%*	*96%*	*96%*
	n° spots matched to every member	458													
